# Non-innocent Role of the Halide Ligand in the Copper-Catalyzed Olefin Aziridination Reaction

**DOI:** 10.1021/acscatal.2c05069

**Published:** 2022-12-23

**Authors:** Manuel R. Rodríguez, Anabel M Rodríguez, Sara López-Resano, Miquel A. Pericàs, M. Mar Díaz-Requejo, Feliu Maseras, Pedro J. Pérez

**Affiliations:** † Laboratorio de Catálisis Homogénea, Unidad Asociada al CSIC, CIQSO-Centro de Investigación en Química Sostenible and Departamento de Química, Universidad de Huelva, 21007 Huelva, Spain; ‡ Institute of Chemical Research of Catalonia, ICIQ, The Barcelona Institute of Science and Technology, Av. Països Catalans, 16, 43007 Tarragona, Spain

**Keywords:** olefin aziridination, nitrene transfer, noninnocent ligands, mechanistic studies, copper catalysis

## Abstract

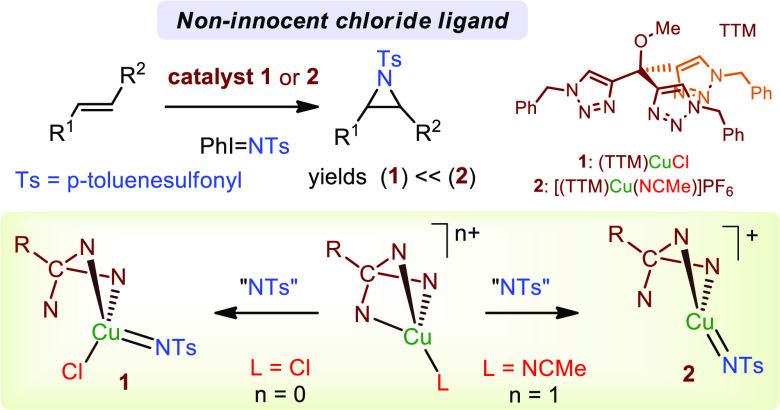

In the context of copper-catalyzed nitrene transfer to olefins, many systems operate upon mixing a CuX salt (X = halide, OTf) and a polydentate N-based ligand, assuming that the X ligand is displaced from the coordination sphere toward a counterion position. Herein, we demonstrated that such general assumption should be in doubt since studies carried out with the well-defined copper(I) complexes (TTM)CuCl and [(TTM)Cu(NCMe)]PF_6_ (TTM = tris(triazolyl)methane ligand) demonstrate a dual behavior from a catalytic and mechanistic point of view that exclusively depends on the presence or absence of the chloride ligand bonded to the metal center. When coordinated, the turnover-limiting step corresponds to the formation of the carbon–nitrene bond, whereas in its absence, the highest barrier corresponds to the formation of the copper–nitrene intermediate.

## Introduction

**Scheme 1:**
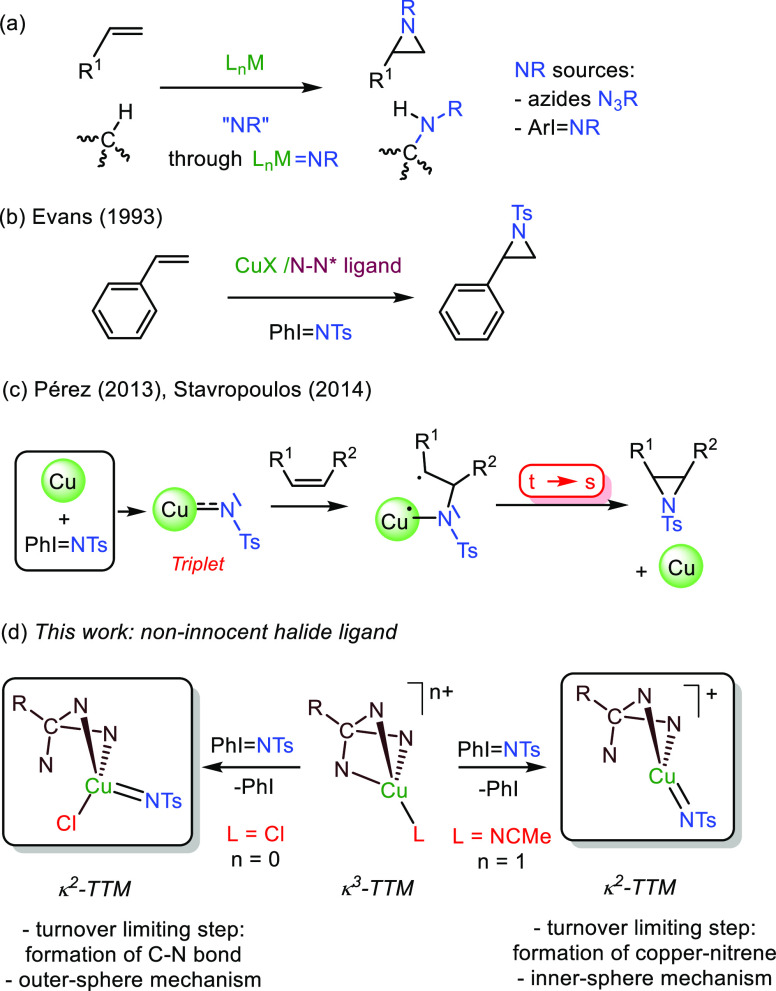
Copper-Catalyzed Olefin Aziridination Reaction

The metal-catalyzed nitrene (NR) transfer reaction constitutes a useful methodology for the formation of carbon–nitrogen bonds from saturated or unsaturated hydrocarbons.^1^ The addition of the NR group to a C=C bond leads to the formation of aziridines, whereas when inserted into a C–H bond, the corresponding amine is formed (Scheme 1a). Albeit discovered in the late 1960s,^2^ it was not until the early 1990s when seminal work by Evans demonstrated^3^ the potential of copper catalysts for the efficient aziridination of alkenes (Scheme 1b), including the asymmetric version.^4^ This transformation occurs through the intermediacy of metal–nitrene species, electrophilic in nature, which have been scarcely detected or isolated.^5^

The catalysts employed for the olefin aziridination reaction can be generally classified into two types.^1^ On the one hand, an in situ generation can be promoted by mixing a copper salt CuX or CuX_2_ (X = Cl, Br, OTf, etc.) with N-donor ligands, with predominant use of bisoxazolines or bipyridines. On the other hand, well-defined catalysts, isolated and characterized, can be employed, as it is, for example, the case of Tp*^x^*CuL (Tp*^x^* = hydrotrispyrazolylborate ligand; L = NCMe, THF), which we have employed in depth for nitrene transfer reactions.^6^ In a previous work, we demonstrated that the mechanism of the olefin aziridination reaction with Tp*^x^*CuL involved both singlet and triplet Cu–nitrene pathways (Scheme 1c),^7^ a new vision in contrast to the almost general belief that each catalytic system for olefin aziridination has a preferred but unique pathway, based on singlet or triplet species, but not both. Our proposal was shortly after reinforced by an excellent work by Stavropoulos, Cronin, Cundari, and co-workers,^8^ employing LCuPF_6_ (L = TMG3trphen, tris[(tetramethylguanidino)-phenyl]amine ligand). For both systems having a tri- or tetradentate ligand, with the LCu core being neutral or cationic, the reaction proceeds through the triplet pathway from a Cu-NR species which later crossed with the singlet pathway, providing explanation to several experiments such as the use of radical inhibitors, competition experiments toward Hammett plots, or the use of olefins with a fixed stereochemistry.

As mentioned above, there are many systems for which a CuX salt and free ligand are employed to generate the catalytic species. The presence of this anionic X ligand constitutes a variable that has not yet been considered in depth from a mechanistic point of view for this transformation, despite the well-known effect of the halide ligands in catalysis.^9^ Since the results based on Tp*^x^*Cu(L) or (TMG3trphen)CuPF_6_ cannot be extrapolated to those generated from CuX, we have now focused on this issue upon using two copper complexes (TTM)CuCl and [(TTM)Cu(NCMe)]PF_6_ bearing a neutral tridentate ligand of type tris(triazolyl)methane (TTM) as catalysts for the olefin aziridination reaction. Herein, we present experimental data that demonstrate a different behavior for both catalysts, which is related to the different location of the X ligand (Cl^–^ or PF_6_^–^) during catalysis. DFT studies have provided an explanation for such findings: the chloride ion remains coordinated to the copper center during catalysis, affecting the reaction pathway in such a way that distinct turnover-limiting steps have been identified (Scheme 1d).

## Results and Discussion

### Catalytic Activity toward Styrene Aziridination of Copper Catalysts

**Scheme 2:**
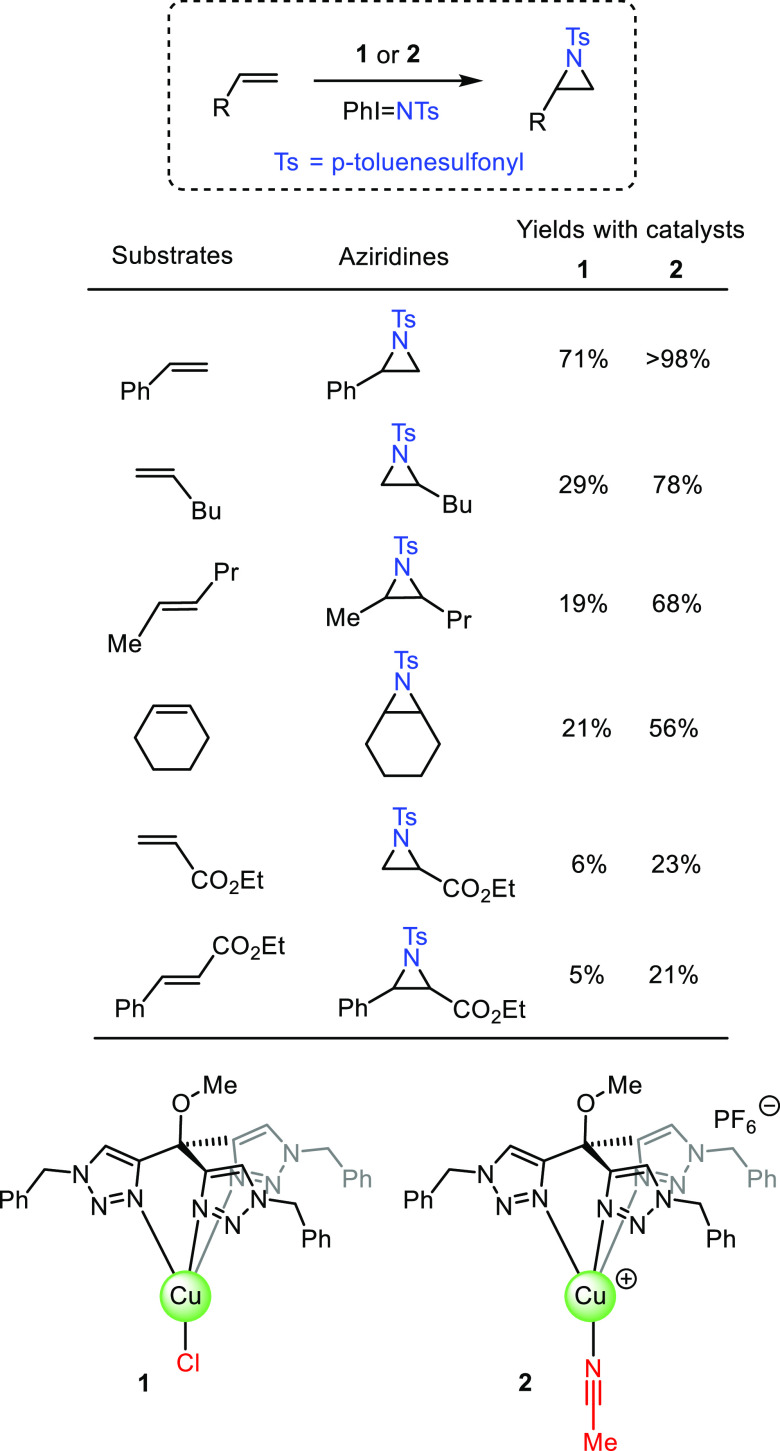
Olefin Aziridination with Catalysts **1** and **2**

Complexes (TTM)CuCl^10^ (**1**) and [(TTM)Cu(NCMe)]PF_6_^11^ (**2**) (TTM = 4,4′,4″-(methoxymethanetriyl)tris(1-benzyl-1H-1,2,3-triazole)) were first employed in the probe reaction of styrene and PhI = NTs as nitrene precursor (Scheme 2, see the Experimental Section for details). The yields into the desired aziridine varied from 71% for **1** to quantitative with **2**, as a first distinction between these catalysts. This difference was observed with an array of olefins such as 1-hexene, 2-hexene, cyclohexene, ethyl acrylate, and ethyl cinnamate (Scheme 2). In all cases, the yields obtained with catalyst **2** were higher than those when employing **1**. Since the (TTM)Cu moiety is present in both cases, the difference must be related to the difference in either charge or the coordination of the chloride. It is worth mentioning that it is frequently assumed that in systems generated from CuX/N–N ligands, the halide is displaced toward an outer-sphere position.^12^ Therefore, based on those literature precedents, proposing chloride extrusion would end into the same (TTM)Cu^+^ cores for both experiments in Scheme 2, from which the observance of different reaction outcomes would be difficult to explain.

There are several experiments that have been traditionally employed as tools to support mechanistic proposals for the metal-catalyzed olefin aziridination reaction. Among them, the following three are crucial to establish the involvement of radical intermediates and/or electrophilic metal–nitrene species: (i) competition experiments with *p*-substituted styrenes and subsequent evaluation of Hammett plots; (ii) the use of olefins with defined stereochemistry and the maintenance or disappearance of the original geometry in the final aziridines; and (iii) the addition of radical inhibitors. We have performed these experiments employing complexes **1** and **2** as catalyst and compared the reaction outcome for both cases.

### Competition Experiments with Styrenes

**Figure 1:**
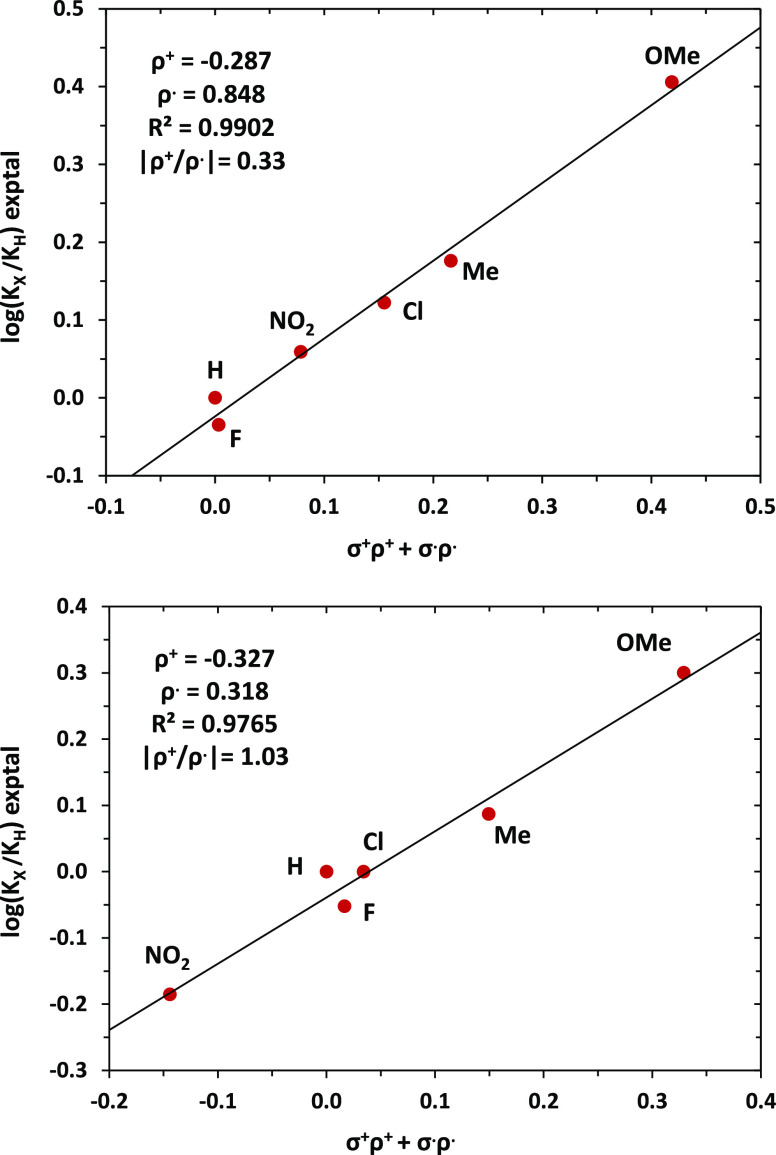
Plot of experimental vs calculated (from multiple regression, see the SI) values of log(*K*_x_/*K*_H_) obtained with neutral catalyst **1** (top) and ionic catalyst **2** (bottom).

Equimolar amounts of styrene and a *p*-substituted styrene bearing either electron-donating or -withdrawing groups were reacted with PhI = NTs and catalytic amounts of **1** or **2**. The ratio of products obtained (Table 1) shows similar behavior for both cases. Electron-rich styrenes are more reactive than styrene, whereas no defined pattern is observed for those with an electron-withdrawing group attached. Because of this, no correlation of the experimental data with the simple Hammett equation log(*K*_X_/*K*_H_) = ρσ can be found. It is only when a dual-parameter Hammett equation log(*K*_X_/*K*_H_) = ρ^+^σ^+^ + ρ^•^σ^•^ is employed that the experimental results can be fitted through a multiple regression, similarly to previous work from our laboratory.^7,13,14^ For the radical contribution, the three scales previously reported by Ji,^15^ Jackson,^16^ and Fisher^17^ have been tested, the best results for both sets being obtained with the former. Figure 1 shows the experimental vs calculated data for log(*K*_X_/*K*_H_) with both catalysts, employing the ρ^+^ and ρ^•^ values derived from the fitting. The polar contributions are similar in both catalysts (ρ^+^ = −0.287 ± 0.018 for **1**; −0.327 ± 0.029 for **2**), although the relative |ρ^+^/ρ^•^| ratio strongly differs: 0.33 for **1** and 1.02 for **2**. From this set of experiments, the participation of radical species with these catalysts seems to be validated, but a substantial difference in the effect of the radicals must exist when moving from **1** (neutral) to **2** (ionic) as catalyst.

**Table 1:**
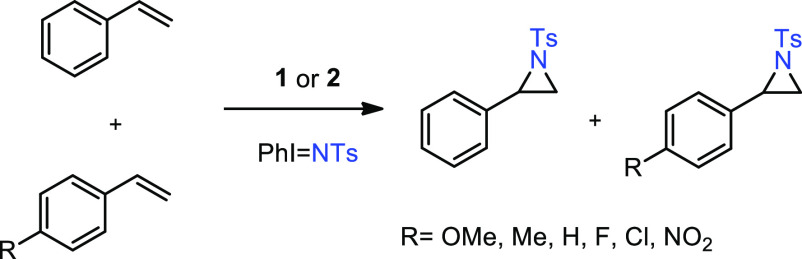
Competitive Aziridination of Styrenes^a^

catalyst	OMe	Me	H	F	Cl	NO_2_
**1**	2.55	1.5	1.00	0.92	1.33	1.15
**2**	1.99	1.22	1.00	0.89	1.00	0.65

a Values correspond to ratio of products relative to that of styrene (*K*_X_/*K*_H_). See the Experimental Section for details.

### Evaluation of *E*/*Z* Olefins

We have employed the olefins (*Z*)-2-pentene and (*E*)-β-methylstyrene as starting materials in the aziridination reactions with catalysts **1** and **2**. As shown in eqs 1 and 2, the aziridines obtained in the four experiments retained the relative geometry from the initial olefin. In previous work from our groups,^7,18^ we observed that copper-based catalysts could induce the change in stereochemistry due to the formation of radical intermediates where a C–C rotation could take place before aziridine ring closing. It seems that this is not the case with these TTM-containing catalysts, a fact that needs to be considered in the mechanistic picture.
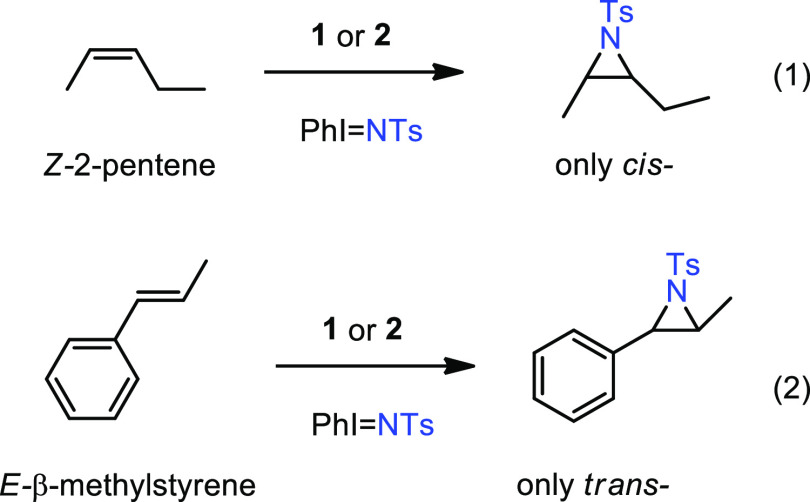
1

### Addition of Radical Inhibitors

**Scheme 3:**
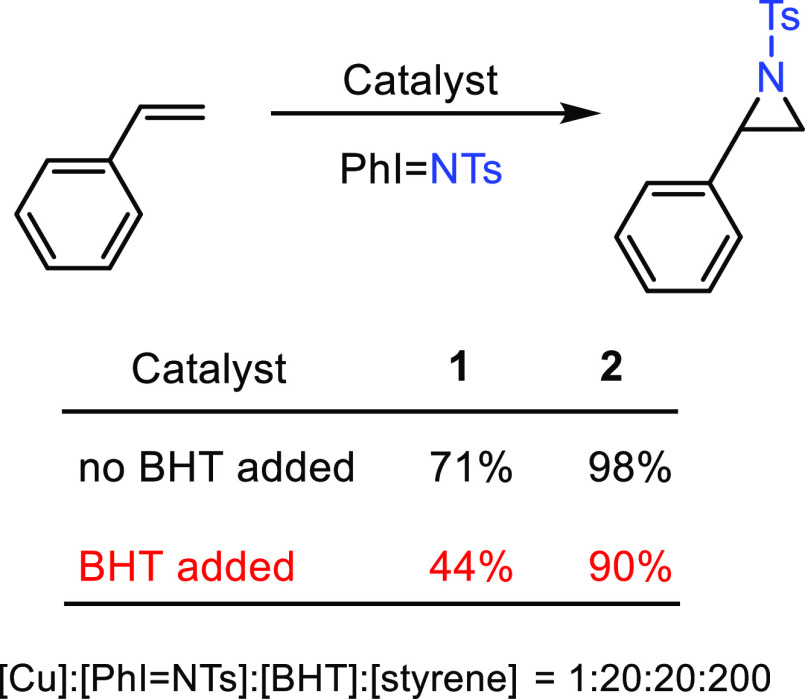
Effect of the Presence of BHT as the Radical Inhibitor

A third set of experiments consisted in the addition of a radical scavenger such as *t*-butylhydroxytoluene (BHT) to the styrene aziridination reaction (Scheme 3). When equimolar amounts of BHT referred to PhI = NTs were incorporated into the reaction mixtures, a certain decrease in aziridine yields was observed, which depended on the catalyst employed. For **1**, the yield dropped from 71 to 44%, whereas in the case of **2**, only a difference of 8% yield (from 98 to 90%) was measured. Again, the involvement of radicals can be associated with both catalysts, but to a different extent.

### Comments from Experimental Data

Collected information from experiments can be summarized as follows: (a) radical species are formed with both catalysts, as assessed from Hammett studies and addition of radical inhibitors; (b) despite that proposal, no modification of the initial geometry of the olefin is observed in the aziridine products; and (c) reaction yields are distinct for each catalyst, the nitrene transfer being necessarily different when comparing **1** and **2** performances.

Albeit (a) and (b) could seem contradictory, our previous work demonstrated that this can be explained in terms of the triplet-to-singlet seam-crossing in the reaction pathway.^7^ The triplet pathway involving radicals may account for (a), whereas the intersection with the singlet pathway before C–C rotation takes place would explain (b). However, catalysts **1** and **2** generate a distinct answer for the radical-influenced probes. This behavior has not yet been explained, and it is highly relevant for those many catalysts in situ generated from copper halides and a ligand.

### DFT Studies

**Figure 2:**
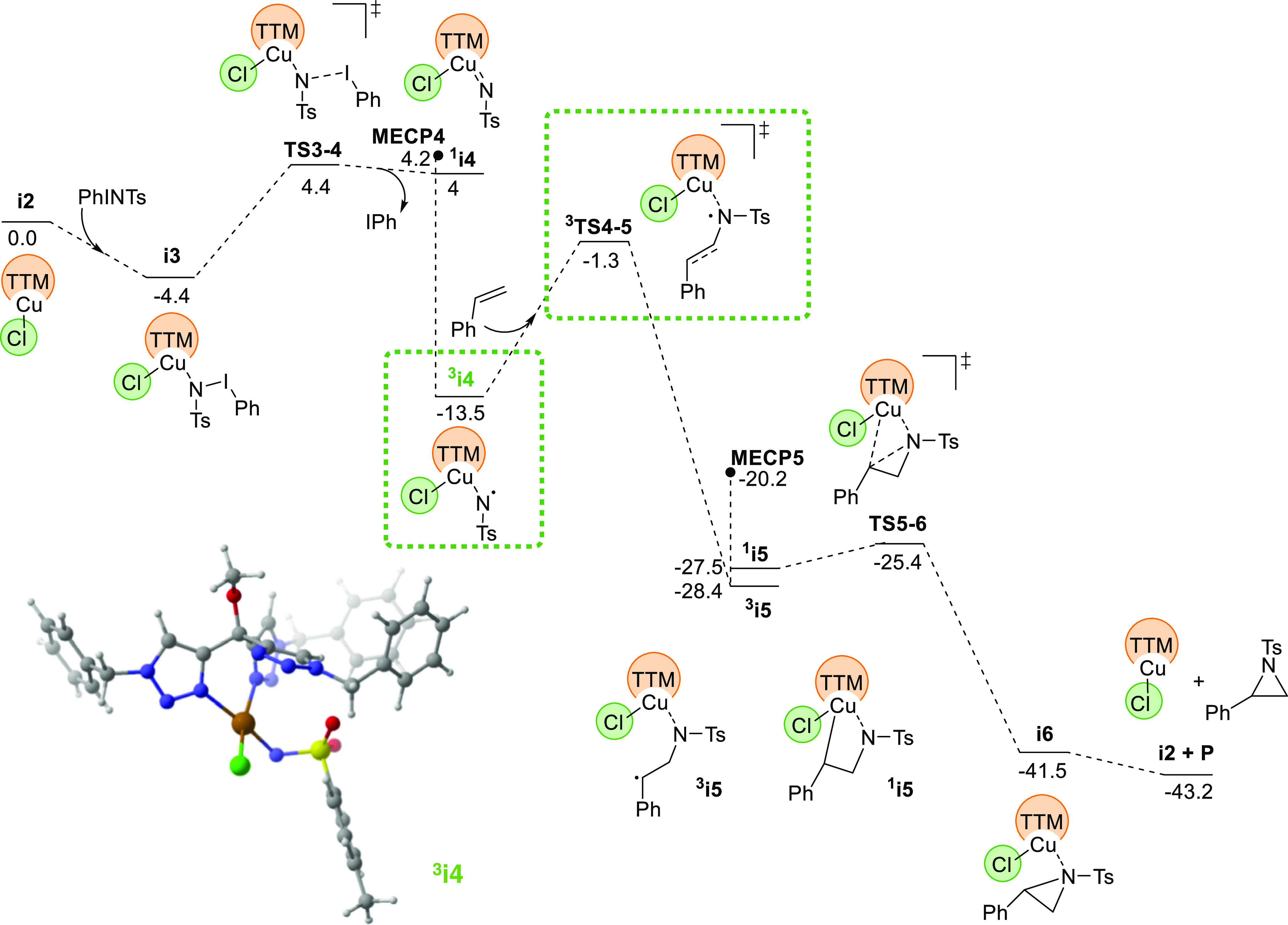
Computed free energy profile for the aziridination of styrene catalyzed by complex **1**. Key species for energy span in green boxes. 3D structure of intermediate ^**3**^**i4** in the lower left corner.

To evaluate the differences between **1** and **2**, DFT studies have been carried out with the B3LYP-D3 functional in solvent. A dataset of all computational results is available in the ioChem-BD repository and can be accessed via https://doi.org/10.19061/iochem-bd-1-255.^19^ The resulting free energy profile is presented in Figure 2. At the beginning, the process follows the general scheme reported previously by us and others for the reaction between metallonitrenes and olefins.^7,8^ The dissociation of iodobenzene from PhI = NTs in the metal coordination sphere leads to intermediate ^**3**^**i4**, a metallonitrene complex in the triplet state, with one unpaired electron in nitrogen and the other in copper. The Mulliken spin densities on key atoms are 0.47 for Cu, 0.16 for Cl, and 1.08 for N. A list of Mulliken spin densities for this and other species is supplied in the Supporting Information. Styrene reacts with this metallonitrene to form a first C–N bond, followed by a spin crossover to return to a singlet state, and the aziridine cycle is closed to produce intermediate **i6**, where the product is coordinated to the catalyst. Exchange of the aziridine with another molecule of PhI = NTs restarts the catalytic cycle. This proposal is consistent with the presence of radicals observed in the radical inhibition experiments. It is also consistent with the heavy involvement of Hammett parameters σ^•^ in the fitting of the experimental results. The identity of the Hammett parameters that should be involved must be examined in the species associated with the turnover determining energetic span step,^20^ which in this case are ^**3**^**i4** and ^**3**^**TS4-5**, highlighted with green boxes in Figure 2. In this step, with a barrier of 12.2 kcal/mol, styrene reacts with the radical nitrene center attached to the metal; thus, the radical involvement is obvious.

**Figure 3:**
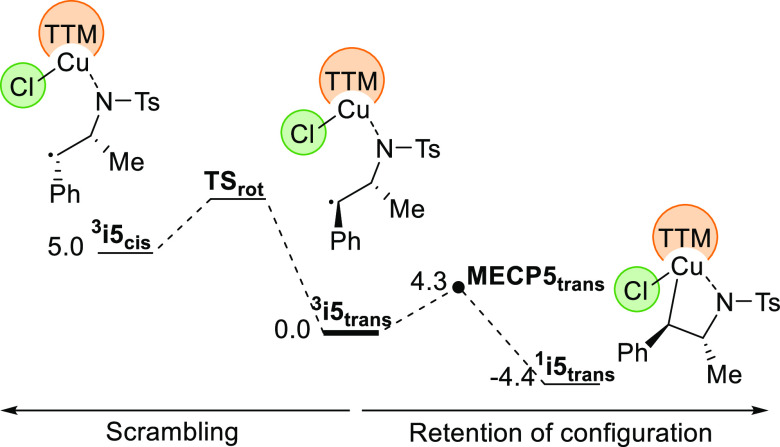
Computed free energy profile for the retention of configuration for (*E*)-β-methylstyrene.

Additional calculations reported in Figure 3 are also consistent with the retention of configuration observed in the case of (*E*)-β-methylstyrene. In that case, the barrier from ^**3**^**i5** to **MECP5** is only 4.3 kcal/mol, which is lower than the energy of the corresponding intermediate leading to the *Z* alternative, which is 5.0 kcal/mol. The corresponding transition state for the *E*–*Z* conversion must be even higher, thus becoming not competitive. This means that the system will evolve to singlet before scrambling the configuration through rotation of the C–C bond.

**Figure 4:**
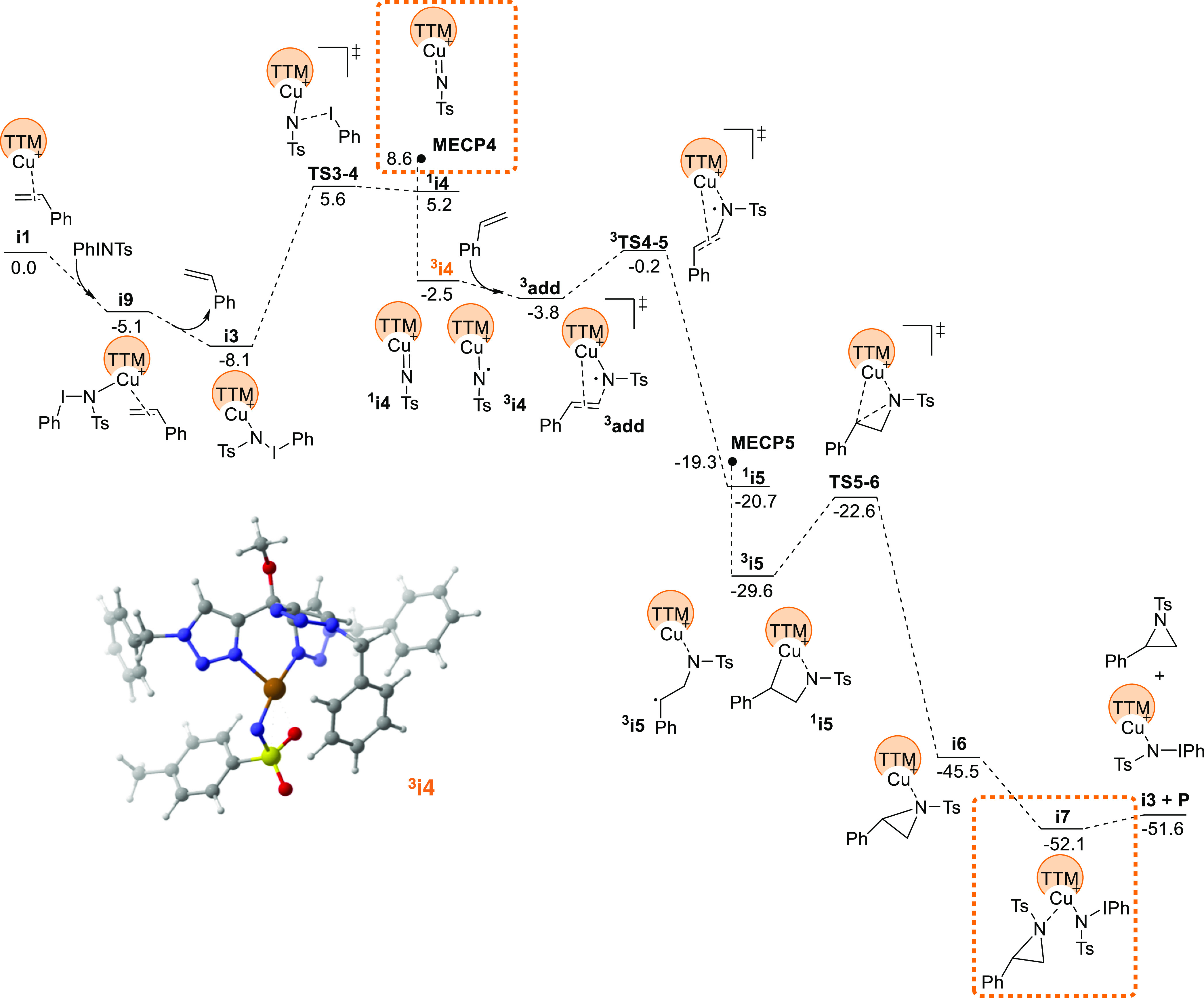
Computed free energy profile for the aziridination of styrene catalyzed by complex **2**. Key species for energetic span in orange boxes. 3D structure of intermediate ^**3**^**i4** in the lower left corner.

We studied next the reaction between the cationic catalyst **2** and styrene. The free energy profile is presented in Figure 4. The qualitative form of the profile is similar to that of catalyst **1**, with the same species involved. There is however a key quantitative difference: the energetic span is no longer defined by the formation of the C–N bond, which now has a barrier of only 3.6 kcal/mol (from ^**3**^**add** to ^**3**^**TS4-5**). The energy span, 17.2 kcal/mol, is instead defined here by the conversion from **i7** to **MECP4**, highlighted in orange boxes in Figure 4. The 17.2 kcal/mol comes from adding the 0.5 kcal/mol required from the departure of product **P** from **i7**, and the 16.7 kcal/mol required from **i3** to **MECP4**. This step includes the cleavage of the N–I bond and formation of the copper nitrene intermediate. This fits well with the increased correlation with the polar form σ^+^ of the Hammett scale. There is a dependence on the styrene because it is present in intermediate **i7** and removed when reaching the high energy point, and the radical dependence is smaller than for catalyst **1** because here the key species are mostly in a singlet spin state. This is moreover in agreement with the minimum effect of radical inhibitors on this system.

Calculations also explain the qualitative difference between the behavior of catalysts **1** and **2**. The TTM ligand is more prone to fluxional processes than the tris(pyrazolyl)borate ligands that are common in this type of chemistry. Because of this, it has access to a κ^2^ coordination, where only two of the three potentially coordinating nitrogen centers remain attached to the copper atom. In the case of catalyst **1**, this empty coordination site is occupied by the chloride ligand, which remains attached to the metal throughout the cycle; see Figure 2. In the cationic catalyst **2**, the counteranion is not coordinating, and in some steps, there is one empty space in the copper coordination sphere; see Figure 4. The extra coordination site in intermediate ^**3**^**i4** allows for easy coordination of the styrene ligand, and this brings down the barrier for the C–N activation in system **2**. Therefore, it is the flexibility of the TTM ligand that leads to an active role for the usually innocent anionic ligand.

### General Mechanistic Picture

**Scheme 4:**
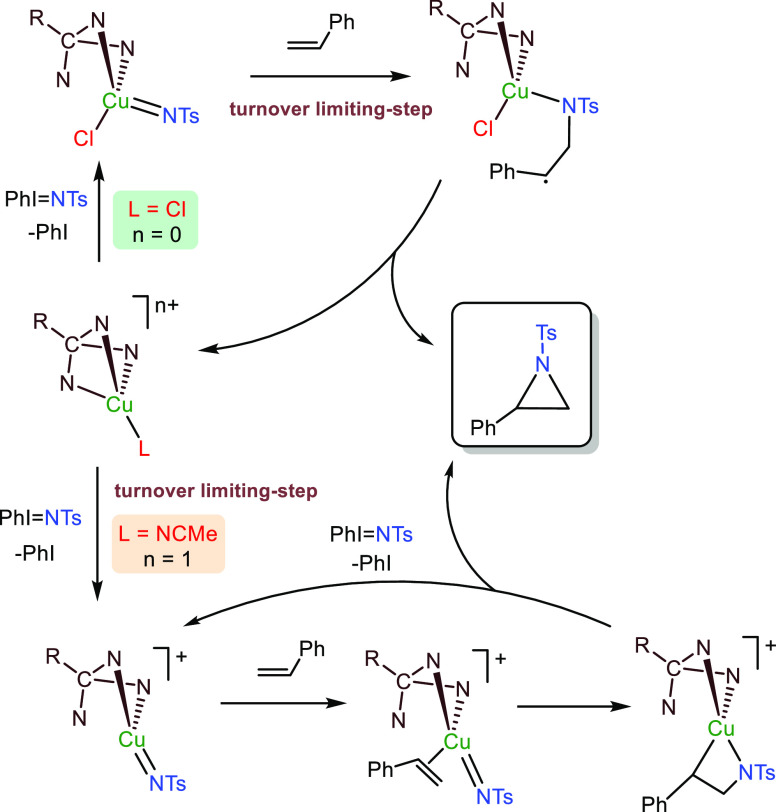
General Mechanistic Picture

From available experimental data and DFT calculations, we have learned that the presence of the chloride ligand in the coordination sphere of copper strongly affects the nitrene transfer reaction to olefins. As shown in Scheme 4, when the halide is coordinated the nitrene ligand reacts with free, noncoordinated styrene following an outer-sphere mechanism. In this case, the formation of the carbon–nitrogen bond displays the highest barrier of the cycle, being the turnover-limiting step. On the other hand, the use of a noncoordinating fragment such as PF_6_ provides a distinct pathway in which the turnover-limiting step is the formation of the nitrene. Styrene coordinates to copper before interaction with the nitrene, this step now being considerably lower in energy compared to that above.

## Conclusions

We have found that the presence or absence of a coordinated chloride in the coordination sphere of copper greatly affects the catalytic behavior toward the olefin aziridination reaction. Experimental data show that yields are different in both cases, as well as the behavior in Hammett-type experiments or toward radical inhibitors. DFT studies indicate that the turnover-limiting steps are different in both cases: the highest barrier in each cycle corresponds to C–N bond formation (with coordinated chloride) or copper–nitrene formation (with no chloride coordinated). These findings must be considered in the context of the commonly employed strategy of generating catalyst upon mixing CuX salts and nitrogenated ligands, where it is assumed that the chloride plays no role during catalysis. This work opens a window for future catalyst design and the differences that might be induced just by leaving or removing the halide ligand from the metal center.

## Experimental Section

### Syntheses of Complexes **1** and **2**

Tris(triazolyl)methane ligand (0.15 mmol, 78 mg), the copper salt (eq 1), and MeCN (3 mL) were added to a Schlenk flask. The mixture was stirred for 12 h at room temperature. MeCN was removed at reduced pressure to obtain a colorless oil. The residue was dissolved in DCM (4 mL), and the DCM was again removed to obtain complexes **1** and **2** as white solids, which were dried under vacuum.^10,11^

### General Olefin Aziridination Experiment

The [TTMCu]X (X = PF_6_, Cl) complex (0.01 mmol) was dissolved in deoxygenated DCM (6 mL) before olefin (1 mmol) was added. PhI = NTs (74.4 mg, 0.2 mmol) was then incorporated in one portion. After 12 h, volatiles were removed under reduced pressure and the crude was analyzed by ^1^H NMR spectroscopy.

### *p*-Substituted Styrenes Competition Experiments

The [TTMCu]X (X = PF_6_, Cl) complex (0.01 mmol) was dissolved in deoxygenated DCM (6 mL). Styrene (1 mmol) and the *p*-substituted styrene (1 mmol) were added. PhI = NTs (74.4 mg, 0.2 mmol) was added in one portion. After 12 h, volatiles were removed under reduced pressure and the crude was analyzed by ^1^H NMR spectroscopy.

### Aziridination of (*E*) and (*Z*) Olefins

The [TTMCu]X (X = PF_6_, Cl) complex (0.01 mmol) was dissolved in deoxygenated DCM (6 mL). The olefin was added (2 mmol), followed by the addition of PhI = NTs (74.4 mg, 0.2 mmol) in one portion. After 12 h, volatiles were removed under reduced pressure and the crude was analyzed by ^1^H NMR spectroscopy.

### Aziridination of Styrene in the Presence of BHT

The [TTMCu]X (X = PF_6_, Cl) complex (0.01 mmol) was dissolved in deoxygenated DCM (6 mL). Styrene (2 mmol) and BHT (0.2 mmol) were added, followed by the addition of PhI = NTs (74.4 mg, 0.2 mmol) in one portion. After 12 h, volatiles were removed under reduced pressure and the crude was analyzed by ^1^H NMR spectroscopy.

### Computational Details

The presented computational mechanistic study was carried out in Gaussian 09^21^ by optimization of minima and transition states without any symmetry restrictions using B3LYP-D3 functional^22^ including Grimme’s empirical dispersion correction.^23^ The 6-31G(d)^24^ basis set was used for all atoms except Cu, for which LANL2DZ.^25^ Unless otherwise stated, the reported energies were obtained from single-point calculations with a larger basis set, LANL2TZ plus pseudopotential for copper,^26^ and 6-311++G(d,p)^27^ for the remaining atoms. All energies reported correspond to triplet or closed singlet spin states. Open-shell singlet states were evaluated for selected intermediates, reported in the Supporting Information, and found not to affect significantly the overall picture. Frequency calculations were carried out at the same level of the geometry optimization to obtain the free energies and ensure the nature of each stationary point. Solvent effects were considered using the SMD^28^ solvation model and default options for dichloromethane (ε = 8.93). For MECP localizations, we used the code provided by Prof. Jeremy Harvey.^29^ The geometries of all species relevant for this study are included in a dataset collection of computational results available in the ioChem-BD repository.^19^

## Supporting Information Available

All procedures and characterization data; additional computational data; and Cartesian coordinates of the optimized structures (PDF)
