# Supplementary material for: Non-innocent Role of the Halide Ligand in the Copper-Catalyzed Olefin Aziridination Reaction
Source: ACS Catal. 2022 Dec 23;13(1):706–13. doi: 10.1021/acscatal.2c05069 (PMC10552652; doi:10.1021/acscatal.2c05069)
Supplement: Supplementary file 1 — cs2c05069_si_001.pdf [file cs2c05069_si_001.pdf]

SUPPORTING INFORMATION

Non-Innocent Role of the Halide Ligand in the Copper-Catalyzed Olefin Aziridination Reaction

Manuel R. Rodríguez,[a] Anabel M. Rodríguez,[a] Sara López-Resano,[b] Miquel A. Pericàs,[b,*] M. Mar Díaz-Requejo,[a,*] Feliu Maseras,[b,*] Pedro J. Pérez.[a,*]

[a]Laboratorio de Catálisis Homogénea, Unidad Asociada al CSIC, CIQSO-Centro de Investigación en Química Sostenible and Departamento de Química, Universidad de Huelva, 21007 Huelva, Spain.

[b]Institute of Chemical Research of Catalonia, ICIQ, The Barcelona Institute of Science and Technology, Av. Països Catalans, 16, 43007 Tarragona, Spain.

perez@dqcm.uhu.es; mmdiaz@dqcm.uhu.es; fmaseras@iciq.es; mapericas@gmail.com

TABLE OF CONTENTS

S1- General Information. S2

S2- Synthesis and Characterization of [TTMCu]Cl (1). S3

S3- Aziridination Reactions. S5

S3.1- Procedure for the p-substituted styrenes competition experiments. S5

S3.2- ^{1}H NMR spectra for the p-substituted styrenes competition experiments S5

S3.3- Procedure for the aziridination of (E) and (Z) olefins. S11

S3.4- ^{1}H NMR spectra for the aziridination of (E) and (Z) olefins. S11

S3.5- Procedure for the aziridination of styrene in the presence of BHT. S14

S3.6- ^{1}H NMR spectra for the aziridination of styrene in the presence of BHT. S14

S3.7- Procedure for the aziridination of other olefins. S16

S4- Hammett Plots. S17

S5- Computational Data. S23

S5.1- Availability in repository of computational results S23

S5.2- Geometry and coordination of the ligand S23

S5.3- Structures, energies and relevant bond lengths of species 3, TS0 and ^{1}4 S24

S5.4- Energy profile from i4 to i5 with calculated multiplicities S25

S5.5- Spin densities of relevant intermediates S26

S5.6- Possible coordination states of the cationic complex S27

S5.7- Cartesian coordinates of the optimized structure S28

S6- References. S76

S1- General Information.

All air- and moisture-sensitive manipulations were carried out with standard Schlenk techniques under nitrogen atmosphere or in a glovebox (MBRAUN UNILAB). Solvents were purchased from commercial sources, dried by distillation under nitrogen atmosphere using the suitable drying agent and deoxygenated immediately before their use. Reagents were acquired from Aldrich and used without any further purification. The tris(triazolyl)methane ligand (4,4',4''-(methoxymethanetriyl)tris(1-benzyl-1H-1,2,3-triazole)),[1] complex **2** ([TTMCu]PF_6),[2] and the nitrene precursor (PhI=NTs)[3] were synthesized by literature procedures. Complex **1** ([TTMCu]Cl) was synthesized as described in section 2. NMR spectra were recorded on the Agilent 400MR spectrometer as solutions at 298 K and referenced to residual solvent peaks: $CDCl_3$ (7.26 ppm ^{1}H, 77.16 ppm ^{13}C), CD_3CN (1.94 ppm ^{1}H, 1.32 ppm ^{13}C). High Resolution Mass Spectroscopy (HRMS) experiments were carried out at the *Centre of Research, Technology and Innovation of the University of Seville* (*CITIUS*).

S2- Synthesis and Characterization of [TTMCu]Cl (1).

The tris(triazolyl)methane ligand (0.15 mmol, 78 mg), CuCl (0.15 mmol, 14.9 mg) and MeCN (3 mL) were added to a Schlenk flask. The mixture was allowed to stir for 12 hours at room temperature. MeCN was removed at reduced pressure to obtain a colorless oil. The residue was dissolved in DCM (4 mL), and the DCM was again removed to obtain complex **1** as a white-greenish solid, which was dried under vacuum.

Complex 1. (88 mg, 95%). **^{1}H NMR** (400 MHz, CD_3CN): δ = 7.84 (br s, 3H), 7.45-7.35 (m, 15H), 5.53 (br s, 6H), 3.06 (br s, 3H). **^{13}C{^{1}H} NMR** (100 MHz, CD_3CN): δ = 148.8, 136.3, 129.9, 129.5, 129.0, 125.8, 73.5, 54.9, 53.1. **HRMS (HESI):** *m/z* 580.1612 [M-Cl]$^+$, calculated for $C_{29}H_{27}CuN_9O^+$: 580.1629.

^{1}H NMR spectrum of complex **1** (400 MHz, CD_3CN).

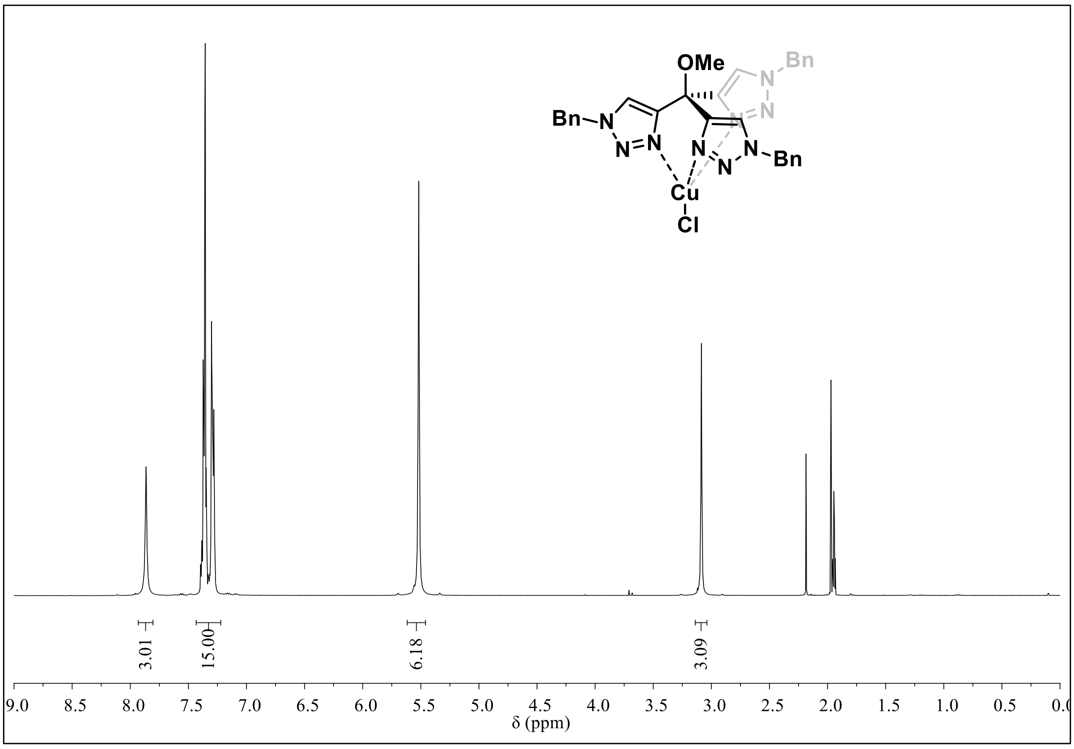

$^{13}C\{^{1}H\}$ NMR spectrum of complex **1** (100 MHz, CD_3CN).

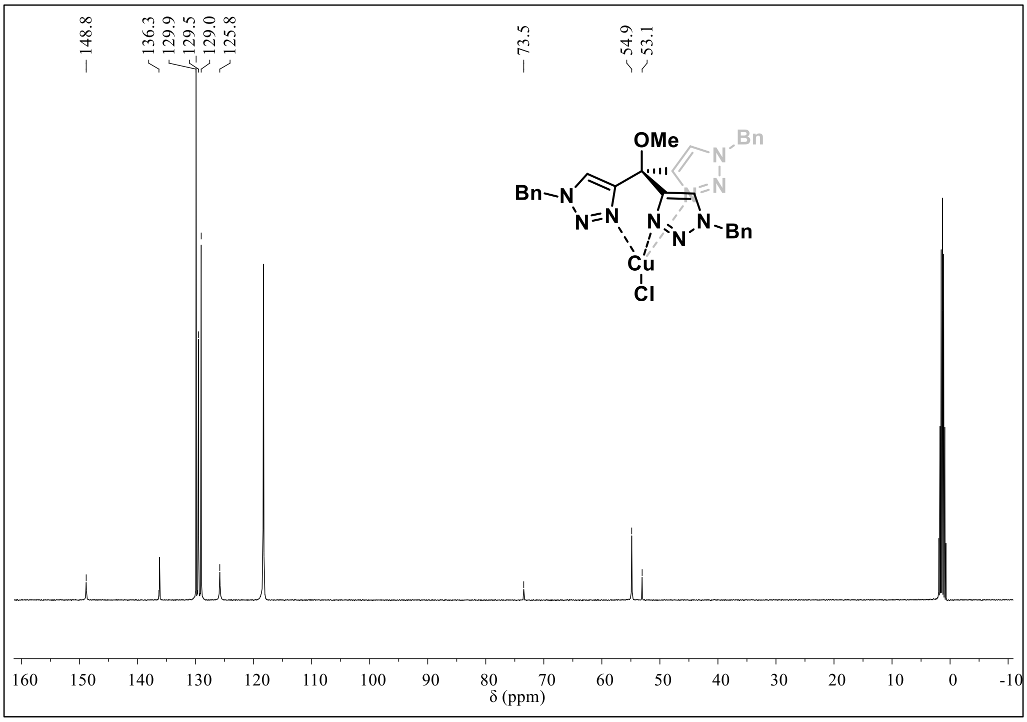

S3- Aziridination Reactions.

S3.1- Procedure for the *p*-substituted styrenes competition experiments.

The [TTMCu]X (X = PF_6 or Cl) complex (0.01 mmol) was dissolved in deoxygenated DCM (6 mL). Styrene (1 mmol, 115 µL) and the *p*-substituted styrene (1 mmol) were added. PhI=NTs (74.4 mg, 0.2 mmol) was added in one portion. After 12 hours, volatiles were removed under reduced pressure and the crude was analyzed by ^{1}H NMR spectroscopy.

S3.2- ^{1}H NMR spectra for the *p*-substituted styrenes competition experiments.

- Employing the complex [TTMCu]Cl (**1**) as catalyst:

^{1}H NMR spectrum for the crude reaction mixture of styrene vs 4-methoxystyrene ($CDCl_3$, 400 MHz).

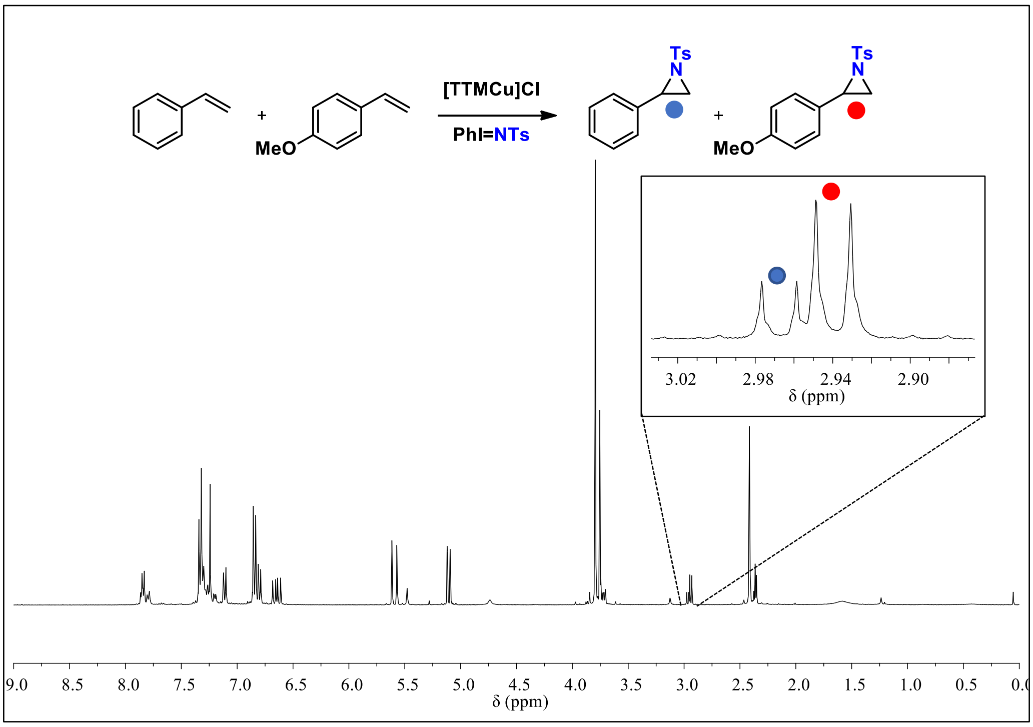

^{1}H NMR spectrum for the crude reaction mixture of styrene vs 4-methylstyrene ($CDCl_3$, 400 MHz).

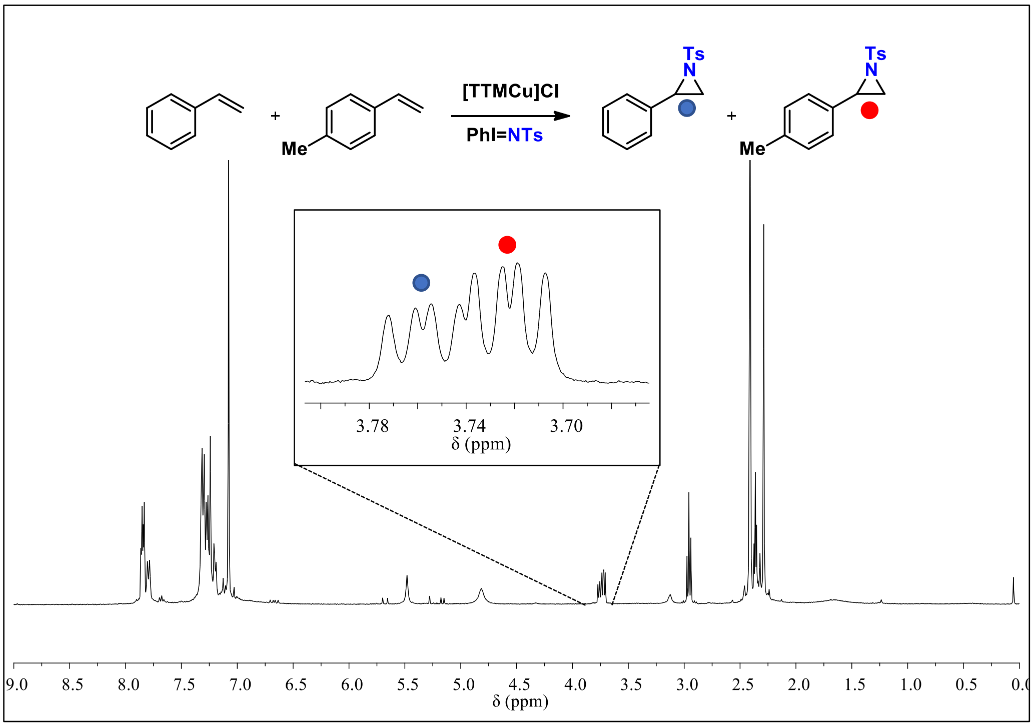

^{1}H NMR spectrum for the crude reaction mixture of styrene vs 4-fluorostyrene ($CDCl_3$, 400 MHz).

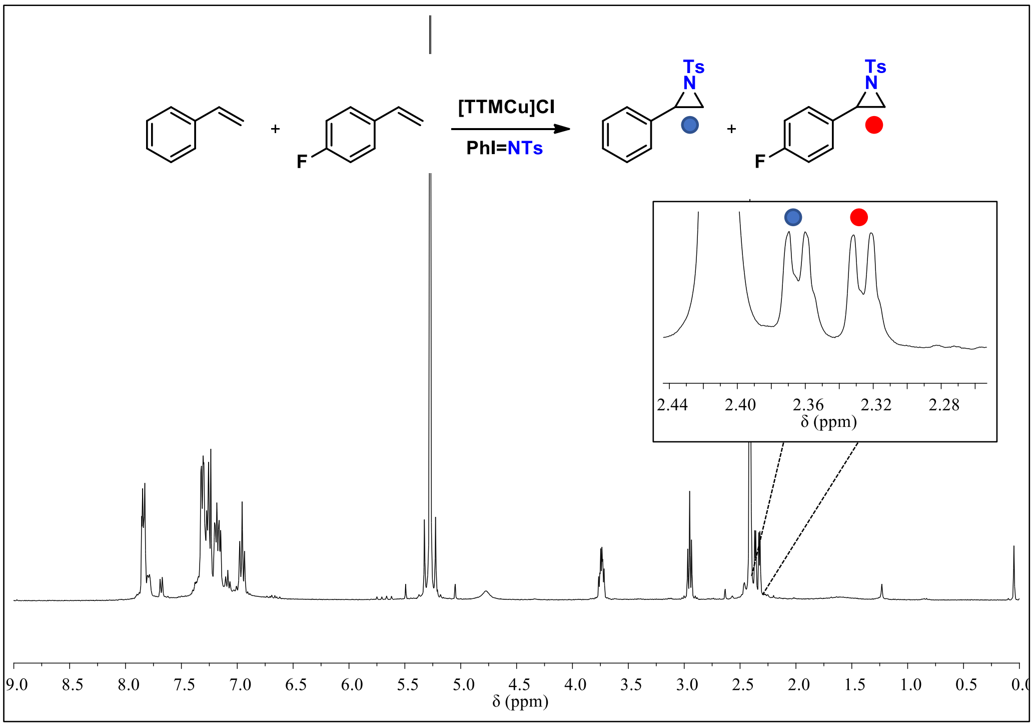

^1H NMR spectrum for the crude reaction mixture of styrene vs 4-chlorostyrene ($CDCl_3$, 400 MHz).

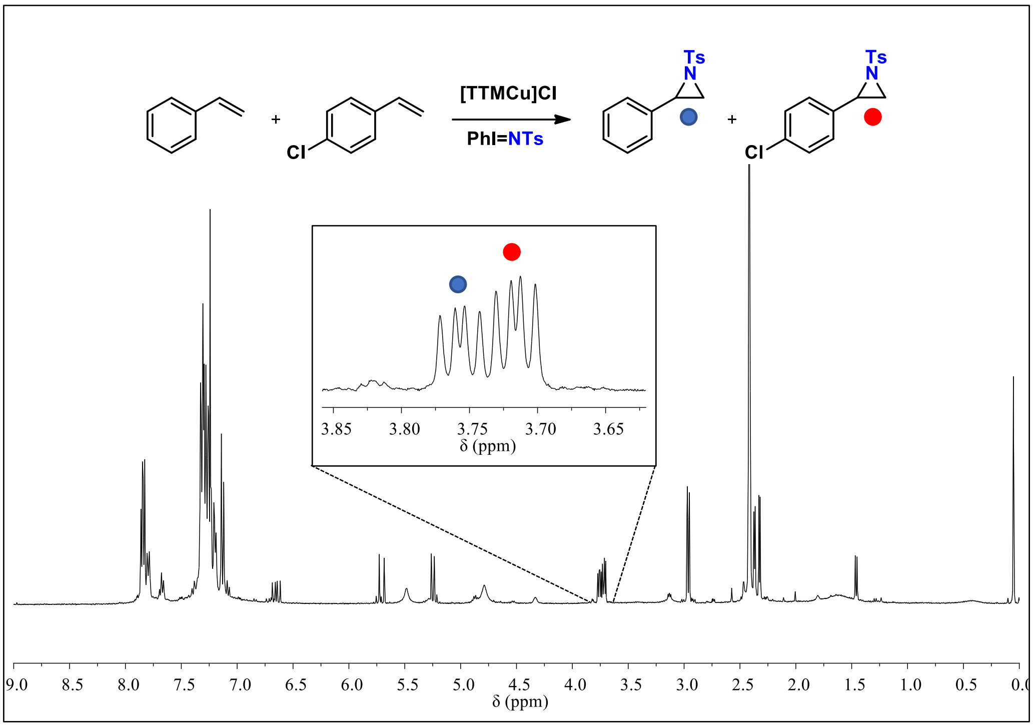

^1H NMR spectrum for the crude reaction mixture of styrene vs 4-nitrostyrene ($CDCl_3$, 400 MHz).

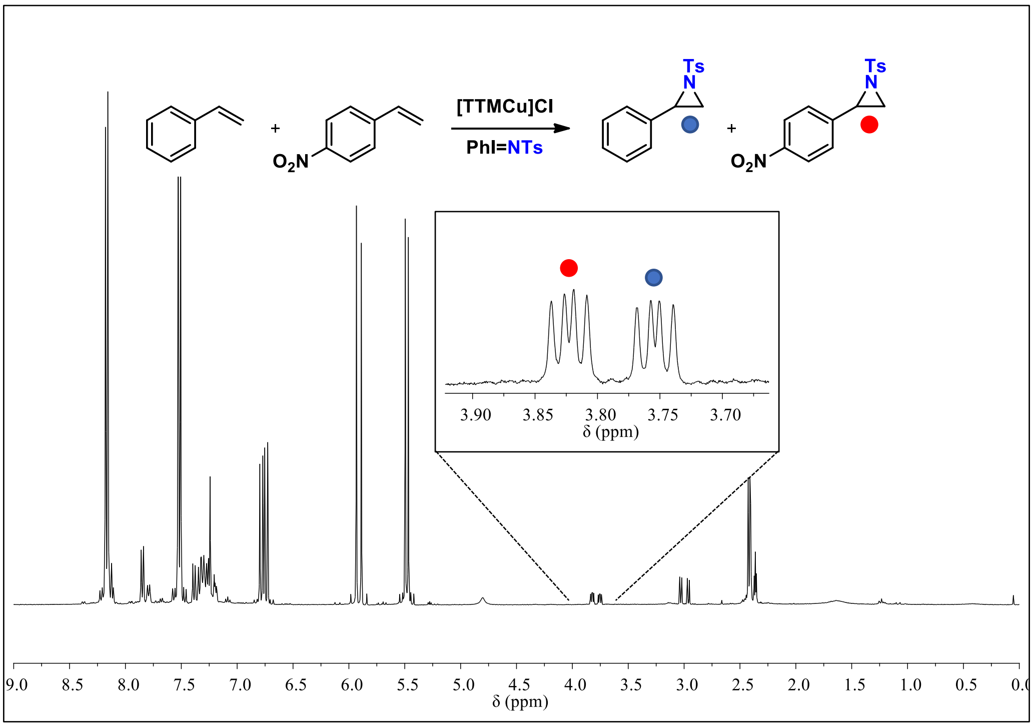

- Employing the complex [TTMCu]PF_6 (**2**) as catalyst:

^{1}H NMR spectrum for the crude reaction mixture of styrene vs 4-methoxystyrene ($CDCl_3$, 400 MHz).

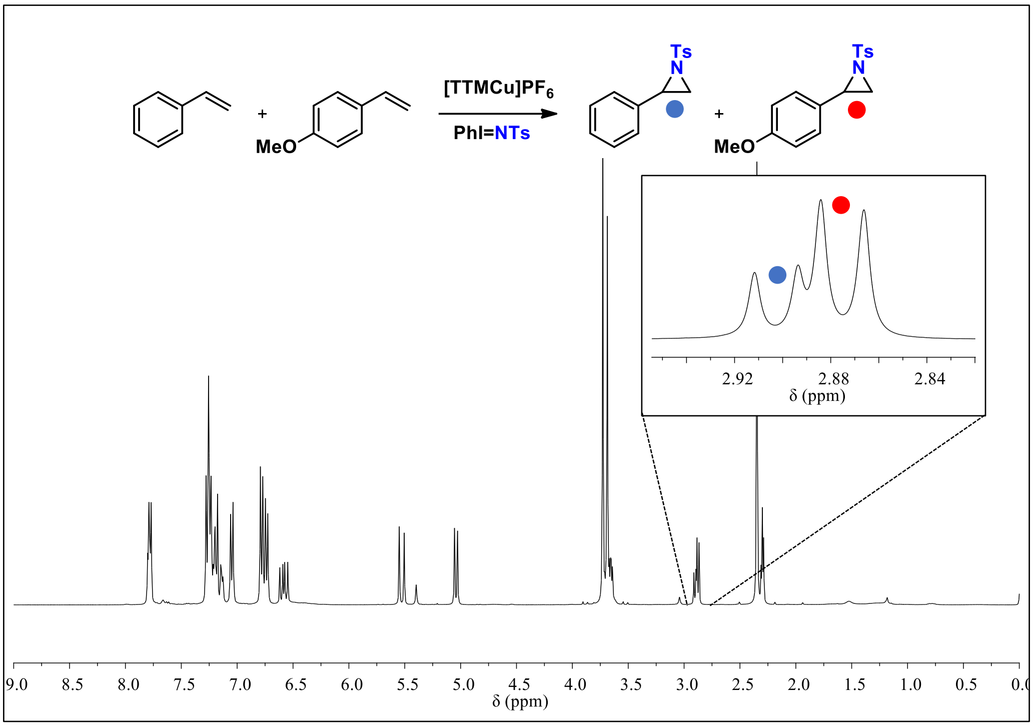

^{1}H NMR spectrum for the crude reaction mixture of styrene vs 4-methylstyrene ($CDCl_3$, 400 MHz).

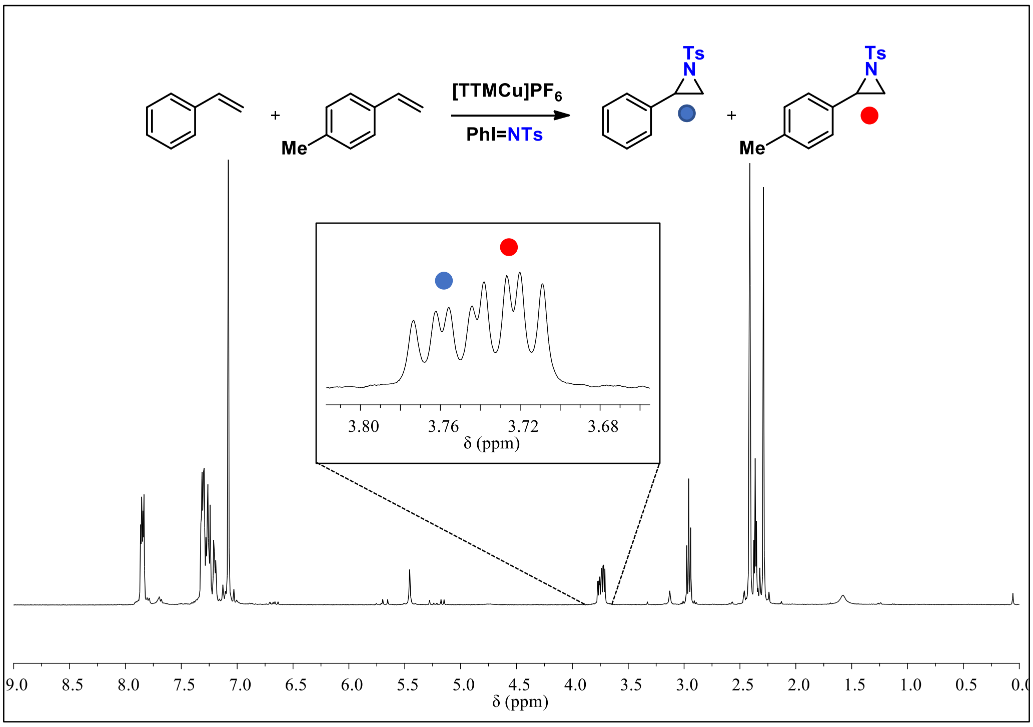

^1H NMR spectrum for the crude reaction mixture of styrene vs 4-fluorostyrene ($CDCl_3$, 400 MHz).

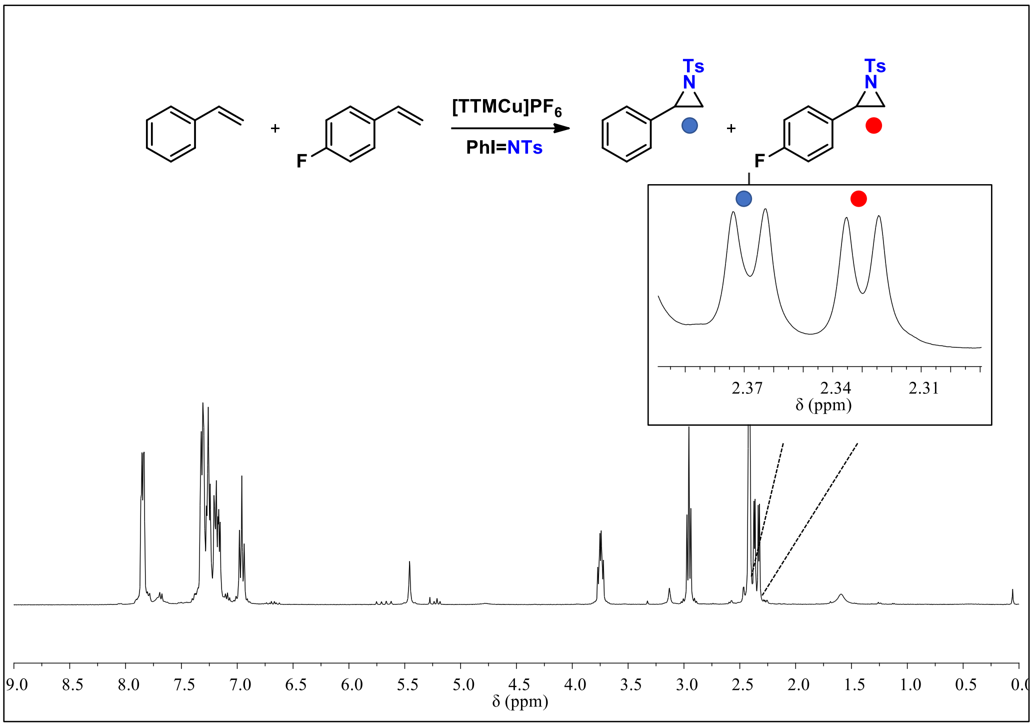

^1H NMR spectrum for the crude reaction mixture of styrene vs 4-chlorostyrene ($CDCl_3$, 400 MHz).

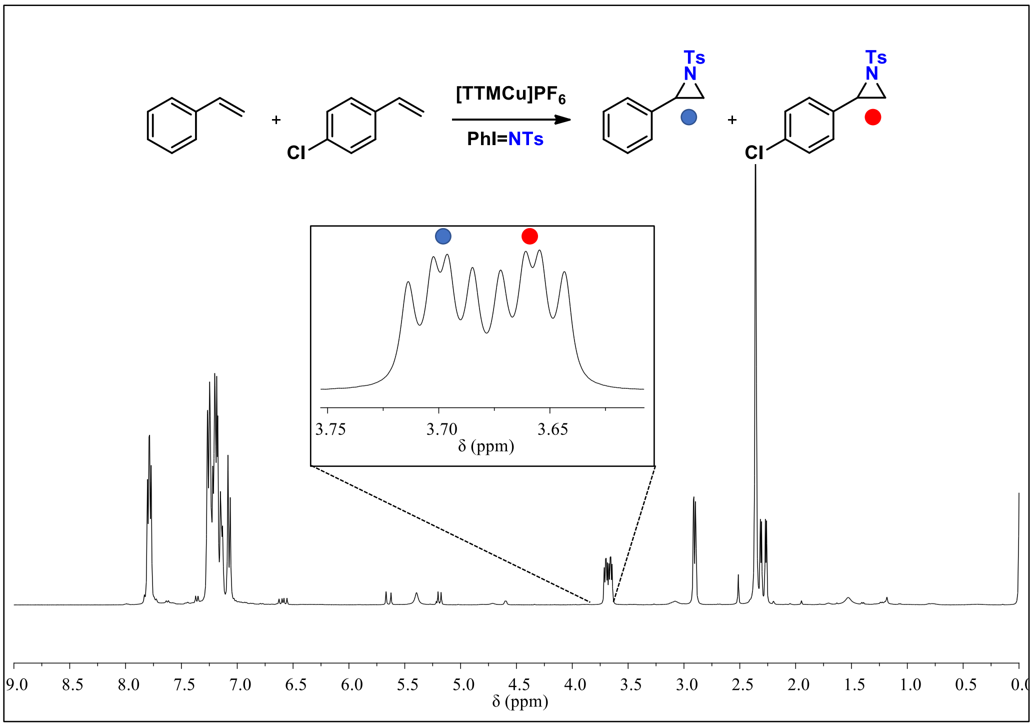

^{1}H NMR spectrum for the crude reaction mixture of styrene vs 4-nitrostyrene ($CDCl_3$, 400 MHz).

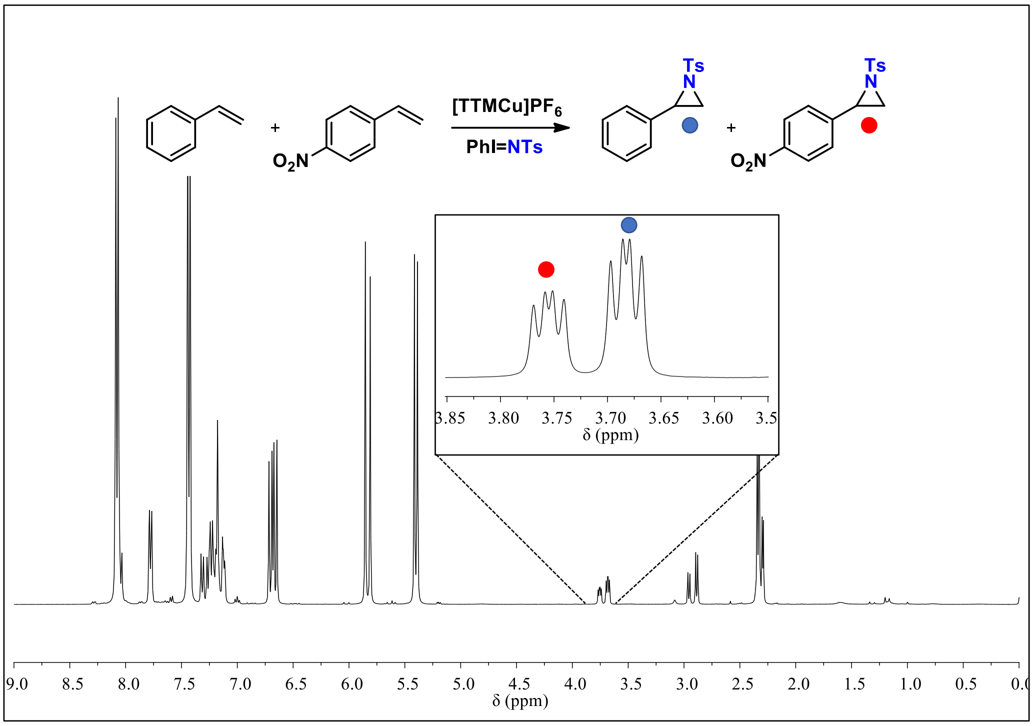

S3.3- Procedure for the aziridination of *(E)* and *(Z)* olefins.

The [TTMCu]X (X = PF_6 or Cl) complex (0.01 mmol) was dissolved in deoxygenated DCM (6 mL). The olefin was added (2 mmol), followed by the addition of PhI=NTs (74.4 mg, 0.2 mmol) in one portion. After 12 hours, volatiles were removed under reduced pressure and the crude was analyzed by ^1H NMR spectroscopy.

S3.4- ^1H NMR spectra for the aziridination of *(E)* and *(Z)* olefins.

- Employing the complex [TTMCu]Cl (**1**) as catalyst:

^1H NMR spectrum for the crude reaction mixture of *trans-β*-methylstyrene ($CDCl_3$, 400 MHz).

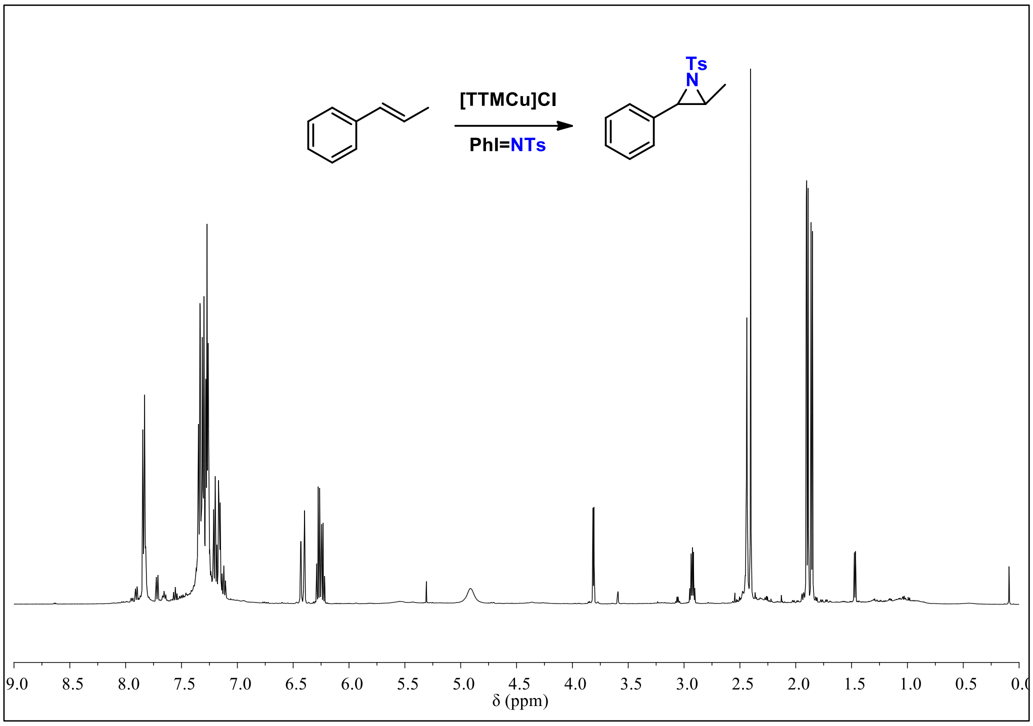

Absence of *cis* aziridine was confirmed according to its spectroscopic data reported in the literature.[4]

^{1}H NMR spectrum for the crude reaction mixture of (*Z*)-pent-2-ene ($CDCl_3$, 400 MHz).

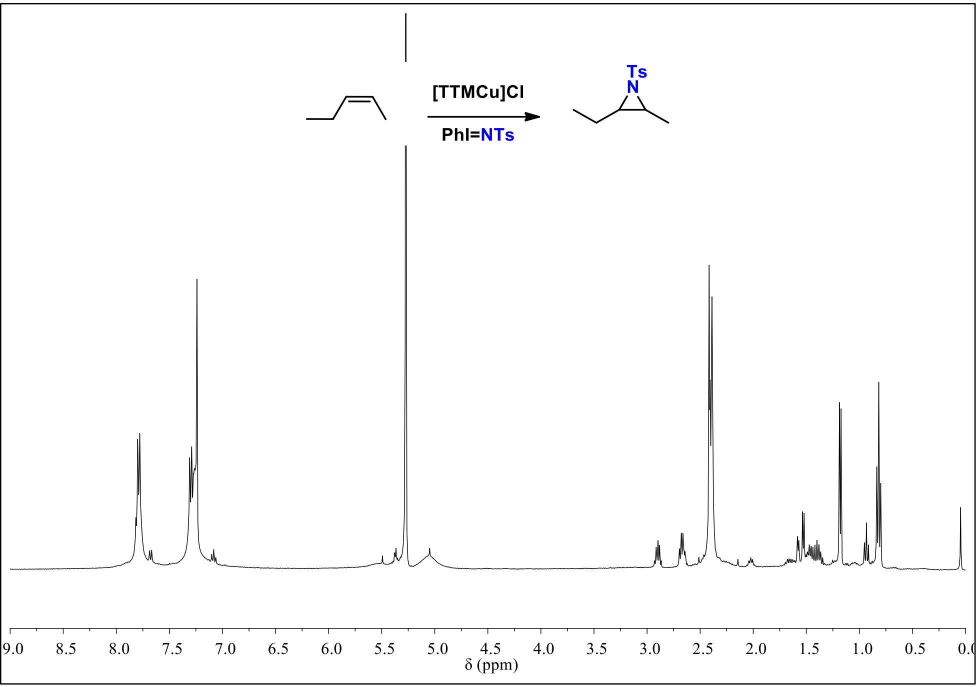

The formation of *cis* aziridine was confirmed according to its spectroscopic data reported in the literature.[5]

- Employing the complex [TTMCu]PF_6 (**2**) as catalyst:

^{1}H NMR spectrum for the crude reaction mixture of *trans*-*β*-Methylstyrene ($CDCl_3$, 400 MHz).

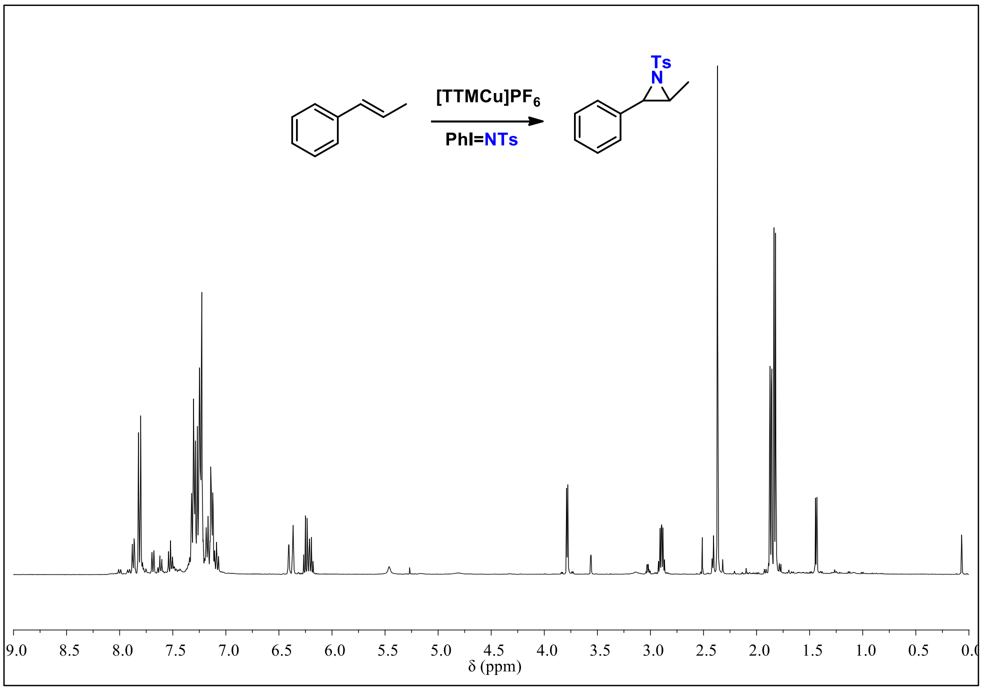

Absence of *cis* aziridine was confirmed according to its spectroscopic data reported in the literature.[4]

^{1}H NMR spectrum for the crude reaction mixture of (*Z*)-pent-2-ene ($CDCl_3$, 400 MHz).

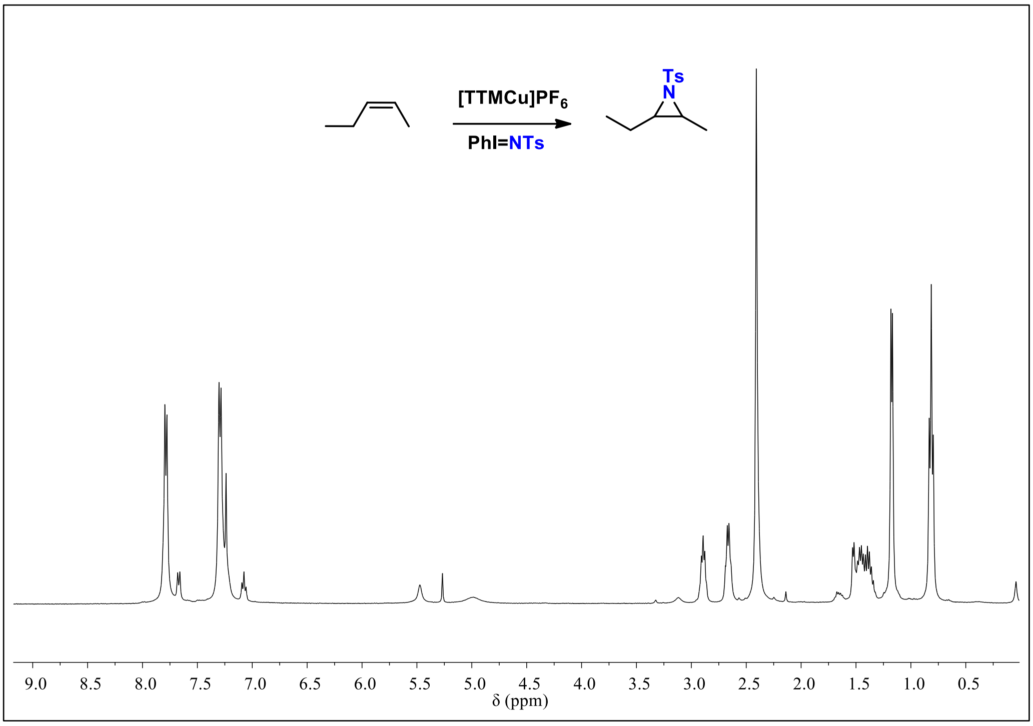

The formation of *cis* aziridine was confirmed according to its spectroscopic data reported in the literature.[5]

S3.5- Procedure for the aziridination of styrene in the presence of BHT.

The [TTMCu]X (X = PF_6 or Cl) complex (0.01 mmol) was dissolved in deoxygenated DCM (6 mL). Styrene (2 mmol, 230 µL) and BHT (0.2 mmol, 44 mg) were added, followed by the addition of PhI=NTs (74.4 mg, 0.2 mmol) in one portion. After 12 hours, volatiles were removed under reduced pressure and the crude was analyzed by ^1H NMR spectroscopy.

S3.6- ^1H NMR spectra for the aziridination of styrene in the presence of BHT.

- Employing the complex [TTMCu]Cl (**1**) as catalyst:

^1H NMR spectrum for the crude reaction mixture of the aziridination of styrene. Top: without BHT. Bottom: with 1 equiv. of BHT ($CDCl_3$, 400 MHz).

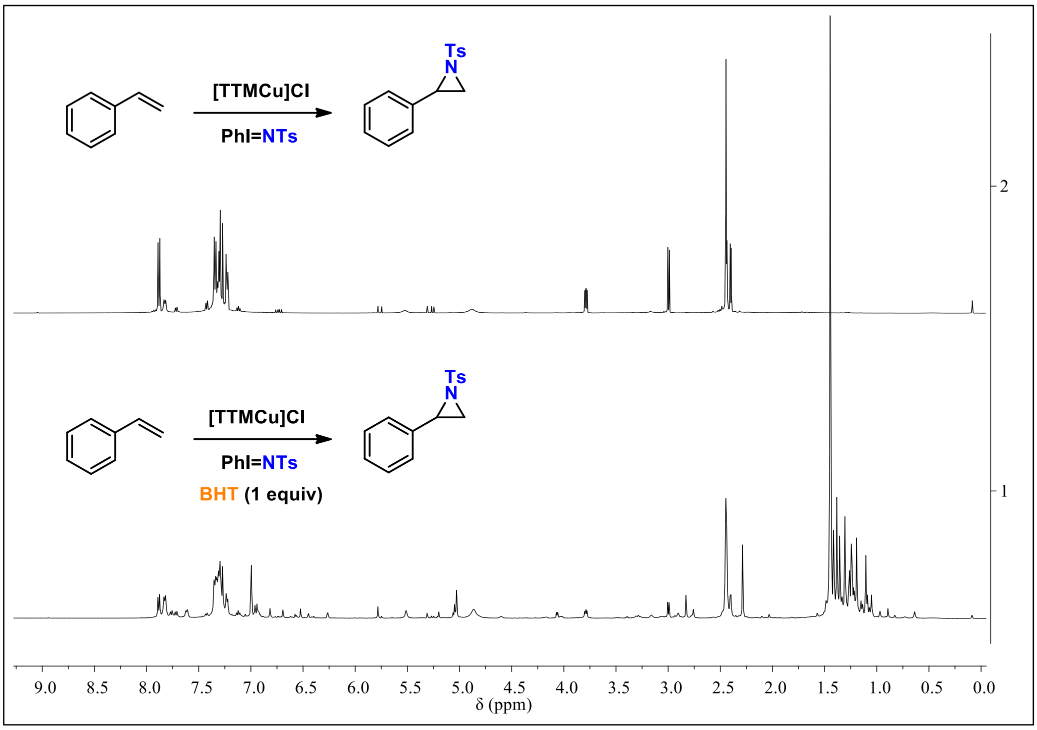

- Employing the complex [TTMCu]PF_6 (**2**) as catalyst:

^1H NMR spectrum for the crude reaction mixture of the aziridination of styrene. Top: without BHT. Bottom: with 1 equiv. of BHT ($CDCl_3$, 400 MHz).

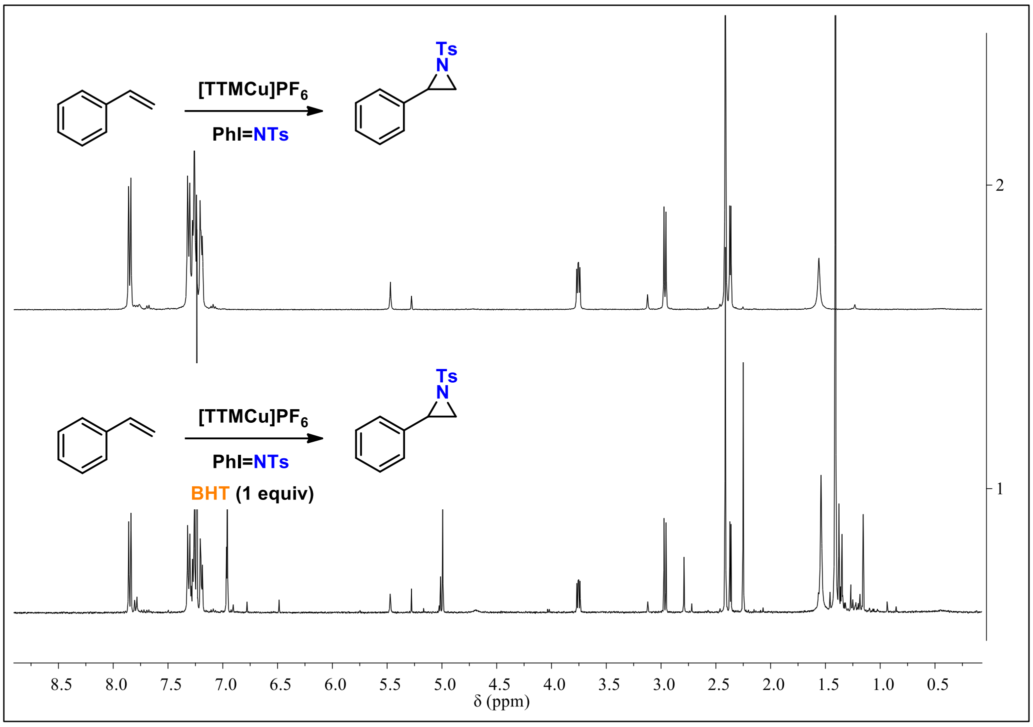

S3.7- Procedure for the aziridination of other olefins.

The [TTMCu]X (X = PF_6 or Cl) complex (0.01 mmol) was dissolved in deoxygenated DCM (6 mL). The olefin was added (2 mmol), followed by the addition of PhI=NTs (74.4 mg, 0.2 mmol) in one portion. After 12 hours, volatiles were removed under reduced pressure and the crude was analyzed by ^{1}H NMR spectroscopy.

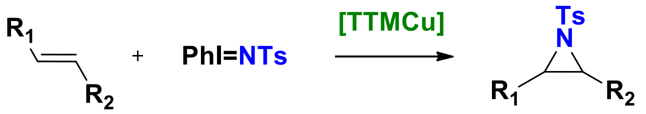

TTMCuCl	TTMCu(NCMe)PF_6	Ref	
29%	78%	6	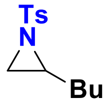
6%	23%	6	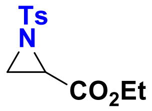
19%	68%	5	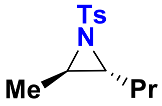
<5%	21%	6	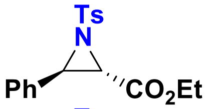
21%	56%	6	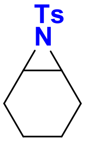

S4- Hammet Plots.

The values of σ^+,[7] $\sigma^\bullet_{Ji}$,[8] $\sigma^\bullet_{Jackson}$[9] and $\sigma^\bullet_{Fisher}$[10] used in the Hammet Plots can be consulted in the provided references.

- Employing the complex [TTMCu]Cl (**1**) as catalyst:

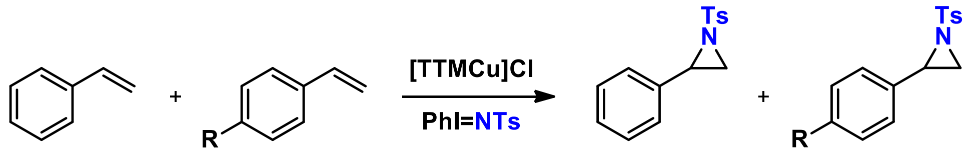

R = H, Me, OMe, F, Cl, NO_2

X	k_X/k_H	$\log(k_X/k_H)$	σ^+	$\sigma^\bullet_{Ji}$	$\sigma^\bullet_{Jackson}$	$\sigma^\bullet_{Fisher}$
**OMe**	2.55	0.41	-0.78	0.23	0.42	-0.12
**Me**	1.50	0.18	-0.31	0.15	0.39	-0.02
**H**	1.00	0.00	0.00	0.00	0.00	0.00
**F**	0.92	-0.03	-0.07	-0.02	0.12	-0.25
**Cl**	1.33	0.12	0.11	0.22	0.18	0.08
**NO_2**	1.15	0.06	0.79	0.36	0.76	0.27

Hammett equation:

$$log(k_x/k_H) = \sigma^+ \cdot \rho^+$$

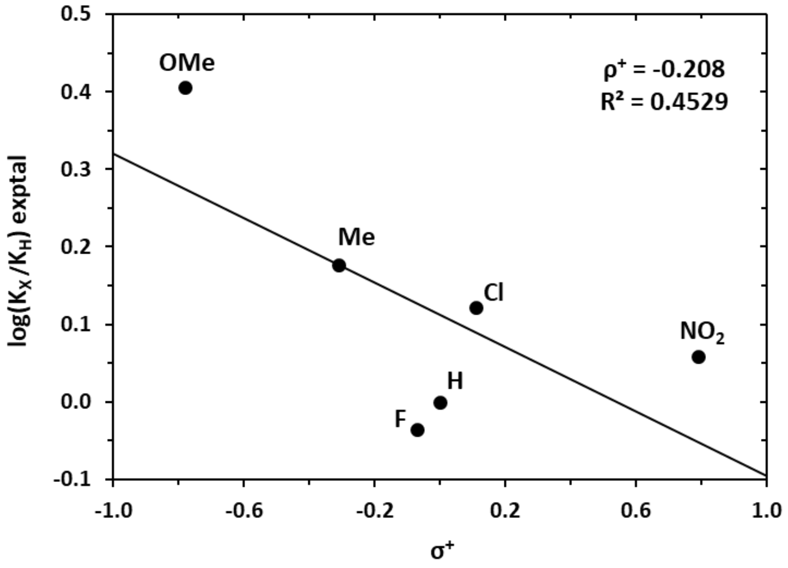

$\boldsymbol{Dual\ parameter\ Hammett\ equation}$:

$$log(k_x/k_H) = \sigma^+ \cdot \rho^+ + \sigma^\cdot \cdot \rho^\cdot$$

- $\sigma^\cdot$ from Ji's Scale:

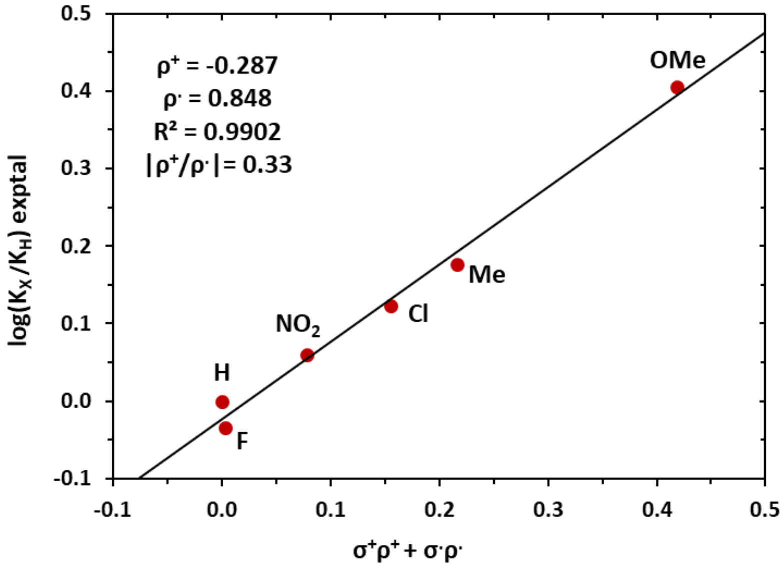

- $\sigma^\cdot$ from Jackson's Scale:

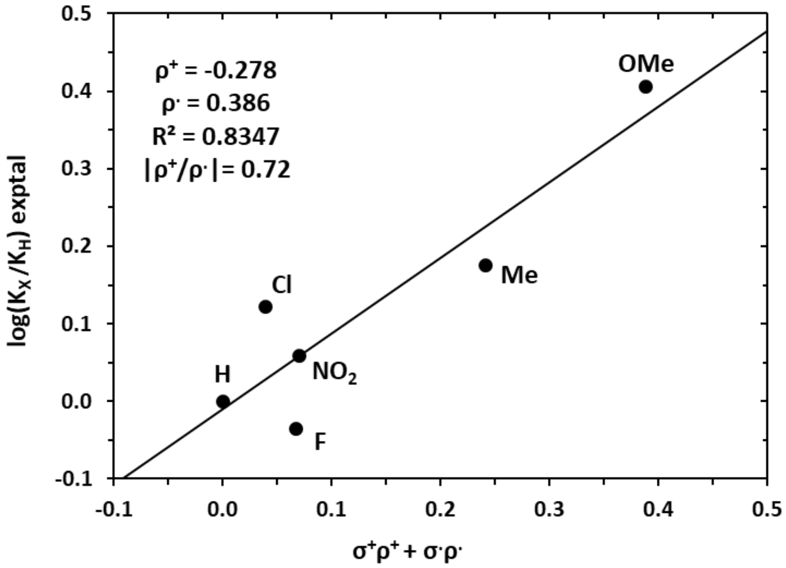

- σ˙ from Fisher's Scale:

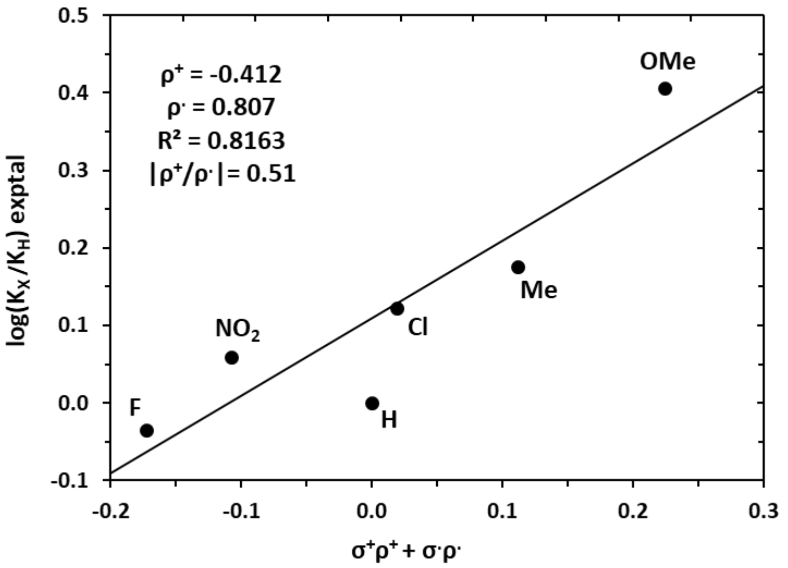

- Employing the complex [TTMCu]PF_6 (**2**) as catalyst:

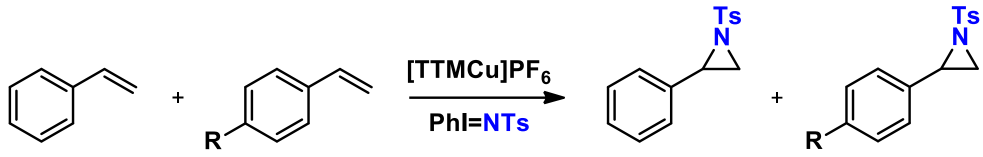

R = H, Me, OMe, F, Cl, NO_2

X	k_X/k_H	$\log(k_X/k_H)$	σ^+	$\sigma^{\bullet}_{Ji}$	$\sigma^{\bullet}_{Jackson}$	$\sigma^{\bullet}_{Fisher}$
**OMe**	1.99	0.30	-0.78	0.23	0.42	-0.12
**Me**	1.22	0.09	-0.31	0.15	0.39	-0.02
**H**	1.00	0.00	0.00	0.00	0.00	0.00
**F**	0.89	-0.05	-0.07	-0.02	0.12	-0.25
**Cl**	1.00	0.00	0.11	0.22	0.18	0.08
**NO_2**	0.65	-0.18	0.79	0.36	0.76	0.27

Hammett equation:

$$\log(k_x/k_H) = \sigma^+ \cdot \rho^+$$

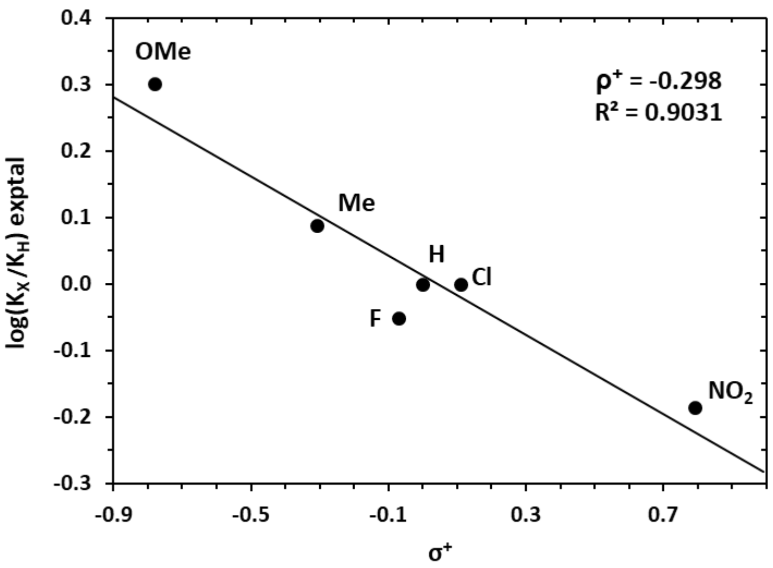

Dual parameter Hammett equation:

$$log(k_x/k_H) = \sigma^+ \cdot \rho^+ + \sigma^{\cdot} \cdot \rho^{\cdot}$$

- $\sigma^{\bullet}$ from Ji's Scale:

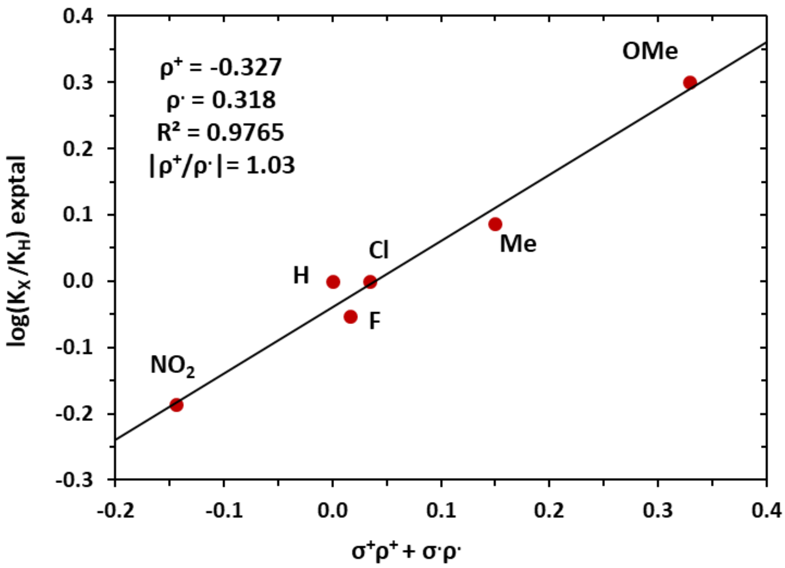

- $\sigma^{\bullet}$ from Jackson's Scale:

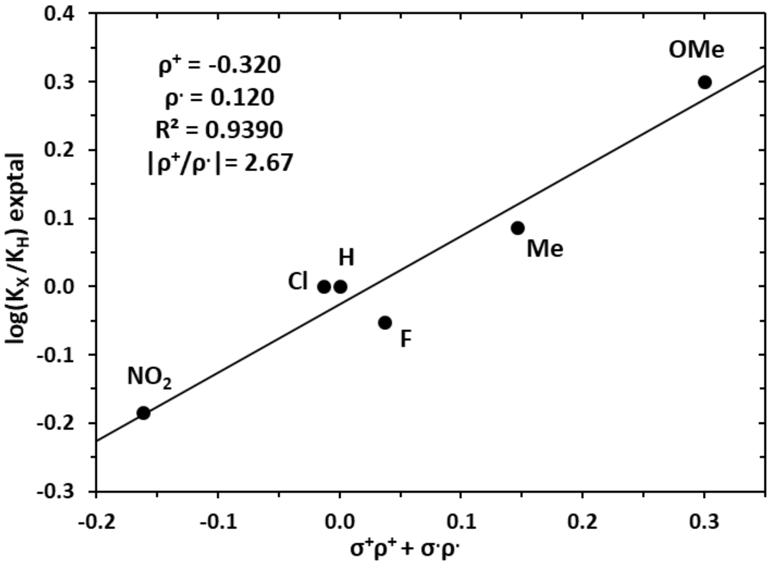

- $\sigma^{\bullet}$ from Fisher's Scale:

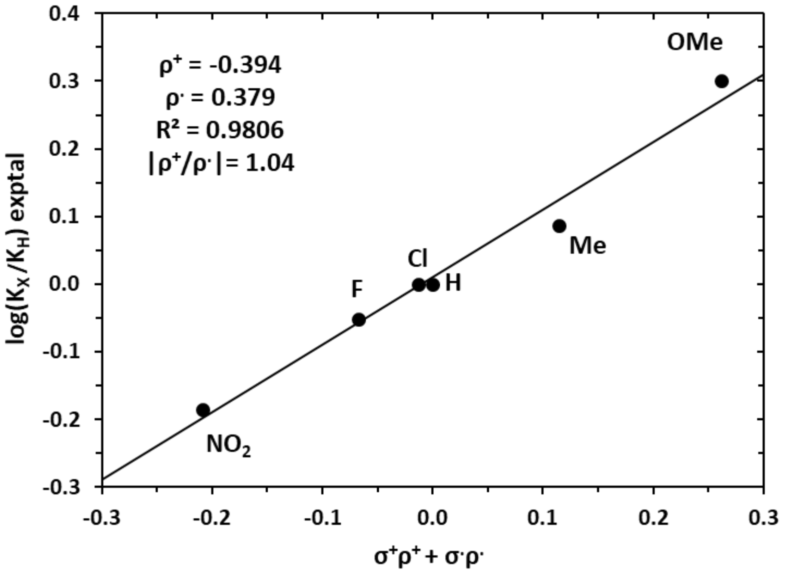

S5- Computational Details.

S5.1- Availability in repository of computational results

A data set collection of computational results is available in the ioChem-BD repository and can be accessed via https://doi.org/10.19061/iochem-bd-1-255.

S5.2- Geometry and coordination of the ligand

The impact of the ligand on the mechanism seems associated to the different flexibility in terms of coordination number. Here we report the most stable structure for the catalyst. The ability of the ligand to disconnect one of the nitrogen atoms differs between the Tpz and TTM ligands, see Figure S1. For the Tpz complex, the three nitrogen atoms are coordinated to the Cu. In the TTMCuCl complex, the halide occupies a third coordination site. Once the halide is removed, only two sites in the coordination sphere are permanently occupied by the ligand.

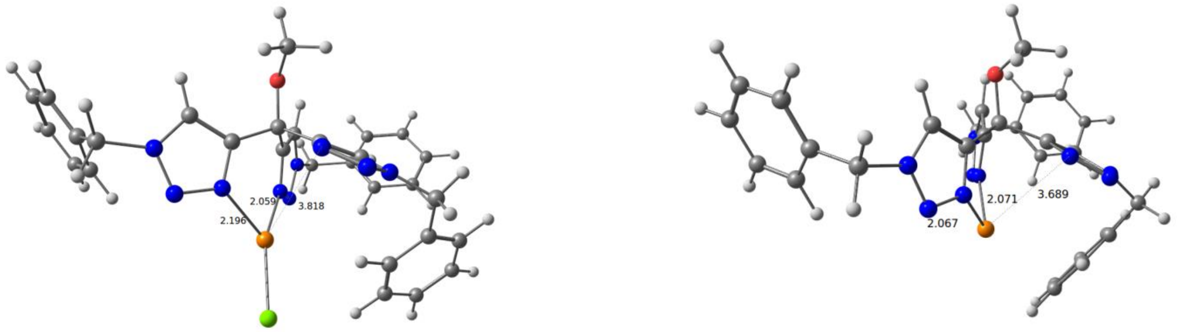

a) b)

Figure S1. Optimized geometry of a) TTMCuCl and b) $TTMCu^{+}$ complexes, distances are in Å

S5.3- Structures, energies and relevant bond lengths of species 3, TS0 and ^{1}4

Structure	Cationic system	Chlorinated system
**i3**	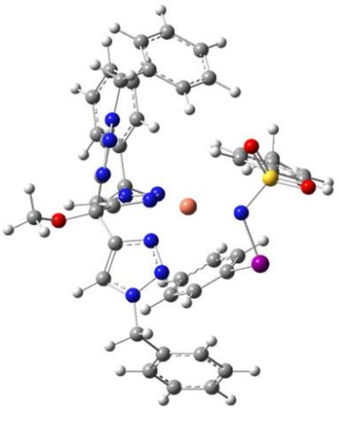	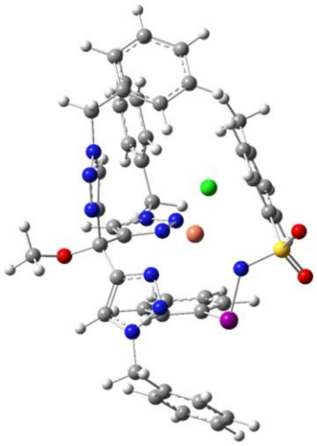
**TS3-4**	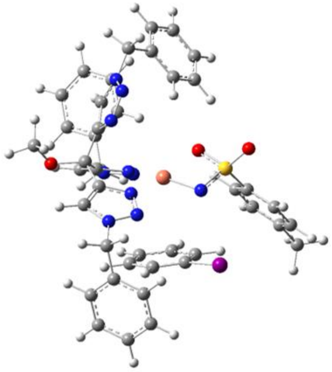	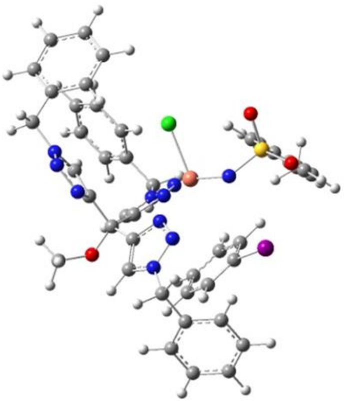
**^{1}i4**	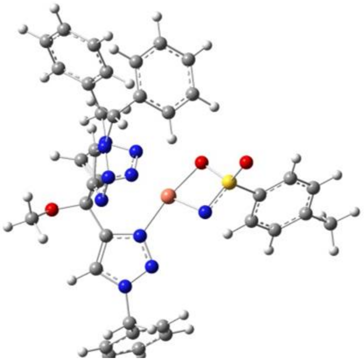	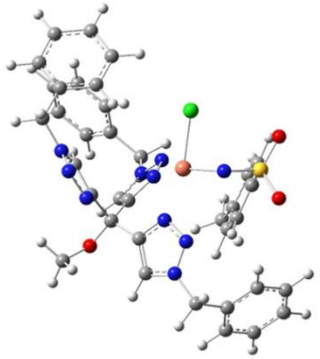

Figure S2: Optimized structures of complexes i**3**, **TS3-4** and **^{1}i4** for both systems

Table S1: Relevant bond lengths (Å) of structures in Fig 2 and their relative energies.

Bond lengths/Å						Relative energies/kcal mol^{-1}
**Cationic system**						
**Structure**	$Cu\text{-}N1_{TTM}$	$Cu\text{-}N2_{TTM}$	$Cu\text{-}N_{PhINTs}$	N-I	Cu-O	
**i3**	2.02	2.18	2.01	2.06		-8.1
**TS3-4**	2.01	2.02	1.89	3.22	1.96	5.6
**^{1}i4**	2.01	1.96	1.89		1.90	5.2
**Chlorinated system**						
**Structure**	$Cu\text{-}N1_{TTM}$	$Cu\text{-}N2_{TTM}$	$Cu\text{-}N_{PhINTs}$	N-I	Cu-Cl	
**i3**	2.18	2.23	2.16	2.03	2.32	-6.1
**TS3-4**	2.05	2.30	1.85	3.12	2.29	0.9
**^{1}i4**	2.09	2.21	1.89		2.25	2.3

S5.4- Energy profile from i4 to i5 with calculated multiplicities

We also calculated the intermediates (**i4** and **TS4-5**) in an open-shell singlet state, however, there are always higher in energy than the triplet states. Here we also add the energy for **^{1}TS4-5** in a singlet state, which is too high to occur.

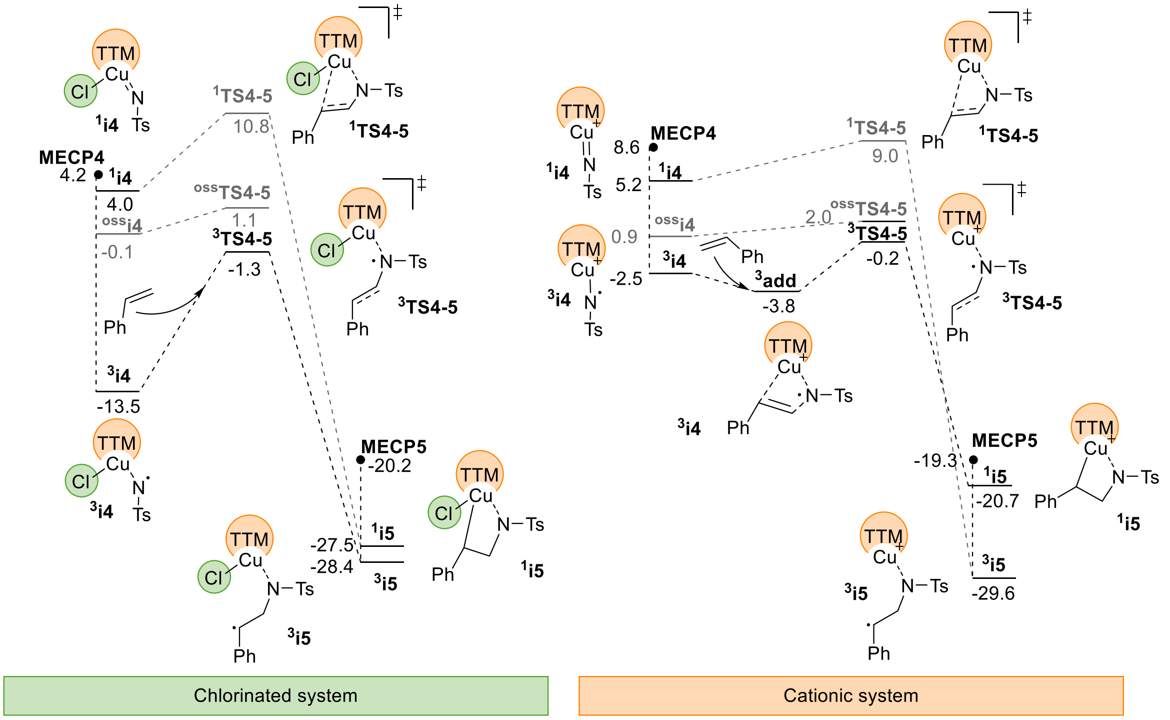

Figure S3: Energy profile for complexes i**4**, **TS4-5** and **i5** in the different multiplicities for both systems

S5.5- Spin densities of relevant intermediates

Here we show the spin densities of the calculated intermediates in Figure S3 in triplet and open-shell singlet states. The C_1 corresponds to the C closest to the Cu and the C_2 to the one attached to the Ph ring.

Table S2: Spin densities of structures on Figure S3.

Spin Densities					
**Cationic system**					
**Structure**	Cu	N	C_1	C_2	
**^{3}i4**	0.49	1.03			
**^{oss}i4**	0.517	-0.654			
**^{3}TS4-5**	0.59	0.91	-0.08	0.28	
**^{oss}TS4-5**	-0.672	0.403	-0.046	0.308	
**^{3}i5**	0.631	0.28	-0.02	0.75	
**Chlorinated system**					
**Structure**	Cu	N	C_1	C_2	Cl
**^{3}i4**	0.47	1.08			0.16
**^{oss}i4**	0.05	-0.08			0.08
**^{3}TS4-5**	0.52	0.92	-0.03	0.26	0.13
**^{oss}TS4-5**	-0.61	0.63	0.02	0.12	-0.13
**^{3}i5**	0.61	0.12	-0.06	0.49	0.13

S5.6- Possible coordination states of the cationic complex.

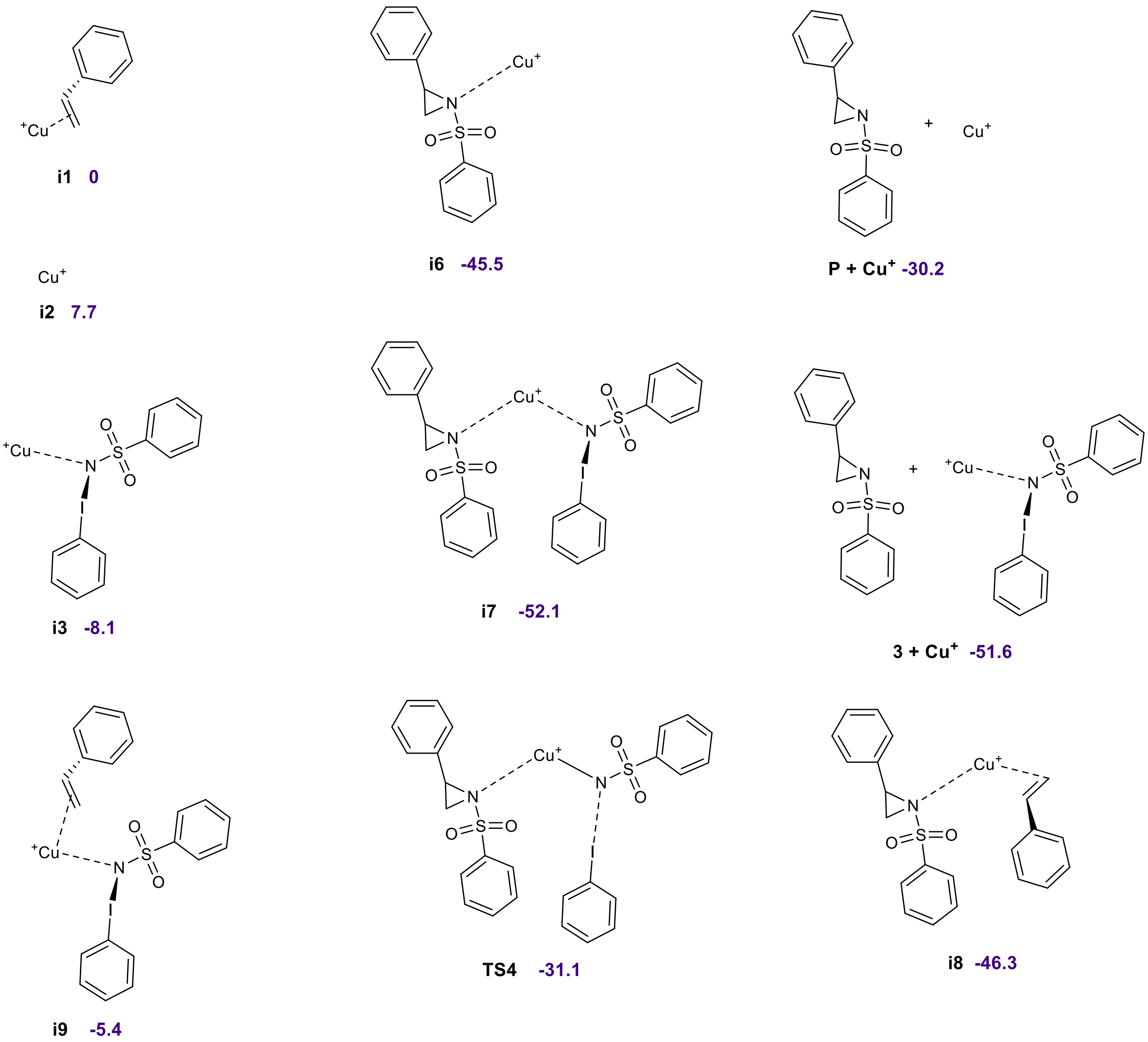

Figure S4: Different coordination possibilities for the cationic copper complex. Relative free energies in kcal mol^{-1}.

S5.7- Cartesian coordinates of the optimized structure

i5-Cl-trans-t-1

	Atom	X	Y	Z
1	C	-0.9138	-0.1161	0.0662
2	C	-0.475	-0.8921	-1.1561
3	C	0.2096	-0.0613	1.0807
4	C	-1.2526	1.2842	-0.4144
5	C	-1.626	1.6858	-1.6825
6	H	-1.7516	1.1571	-2.6151
7	C	-1.2005	-1.7494	-1.9474
8	H	-2.1872	-2.1722	-1.8506
9	C	0.2987	-0.5273	2.373
10	H	-0.4002	-1.0331	3.0197
11	N	1.5591	-0.2039	2.7637
12	N	-1.7926	3.0209	-1.5757
13	N	-0.3956	-1.9809	-3.017
14	N	-1.21	2.3865	0.3887
15	N	-1.5307	3.4394	-0.3166
16	N	0.757	-1.3159	-2.9174
17	N	0.7089	-0.6556	-1.7885
18	N	1.4112	0.4925	0.7706
19	N	2.2311	0.4098	1.7913
20	Cu	2.3071	0.5658	-1.1093
21	O	-2.0232	-0.8352	0.5982
22	C	-2.9159	-0.0964	1.4374
23	H	-3.5103	0.6217	0.86
24	H	-3.5819	-0.84	1.8819
25	H	-2.3886	0.4403	2.2345
26	C	-0.7689	-2.5926	-4.2986
27	H	0.1692	-2.7988	-4.8191
28	H	-1.2752	-3.5368	-4.0888
29	C	-2.0495	3.9839	-2.6458
30	H	-2.9628	3.6845	-3.1668
31	H	-2.2413	4.9367	-2.1437
32	C	2.2411	-0.5193	4.0204
33	H	2.6201	0.4189	4.4343
34	H	1.4714	-0.8968	4.6995
35	C	-1.6532	-1.643	-5.0862
36	C	-1.2283	-0.327	-5.3238
37	C	-2.8996	-2.0589	-5.5636
38	C	-2.0443	0.5599	-6.0266
39	H	-0.2571	0.0018	-4.9668
40	C	-3.7143	-1.1721	-6.2755
41	H	-3.2358	-3.0764	-5.3781
42	C	-3.2905	0.1389	-6.5041
43	H	-1.7012	1.5759	-6.2028
44	H	-4.6814	-1.5055	-6.6422
45	H	-3.9265	0.8295	-7.0515
46	C	3.3677	-1.5241	3.8418
47	C	4.3395	-1.617	4.8471
48	C	3.4576	-2.358	2.722
49	C	5.3838	-2.5368	4.7375
50	H	4.2832	-0.9605	5.7126
51	C	4.5065	-3.2742	2.6117
52	H	2.7455	-2.2779	1.9076
53	C	5.4696	-3.3701	3.6174
54	H	6.1351	-2.5948	5.5208
55	H	4.5771	-3.8955	1.7236
56	H	6.2896	-4.0769	3.5235
57	C	-0.8824	4.0887	-3.6108
58	C	-1.1249	4.2633	-4.9786
59	C	0.439	4.0397	-3.147
60	C	-0.0595	4.4083	-5.8721
61	H	-2.1486	4.2887	-5.345
62	C	1.5023	4.1761	-4.0404
63	H	0.6483	3.8864	-2.0919
64	C	1.2566	4.3694	-5.4036
65	H	-0.2589	4.5455	-6.9316
66	H	2.5196	4.1289	-3.663
67	H	2.0858	4.481	-6.0971
68	N	3.6978	-0.3239	-2.1918
69	S	4.1042	-1.7394	-1.5285
70	O	3.0001	-2.0969	-0.5985
71	O	4.5068	-2.7398	-2.5466
72	C	5.5422	-1.4889	-0.4723
73	C	5.4621	-0.5298	0.5446
74	C	6.6869	-2.2674	-0.6272
75	C	6.534	-0.3692	1.4151
76	H	4.5667	0.0713	0.6652
77	C	7.757	-2.0932	0.256
78	H	6.7349	-3.0039	-1.4224
79	C	7.6949	-1.1529	1.2914
80	H	6.4646	0.3625	2.2165
81	H	8.6489	-2.7042	0.1398
82	C	8.828	-0.9922	2.2749
83	H	8.5152	-1.3046	3.2799
84	H	9.6974	-1.5943	1.9914
85	H	9.1469	0.0546	2.3496
86	Cl	3.0822	2.7427	-0.8447
87	C	3.6045	0.831	-4.3321
88	C	4.5142	0.1926	-3.3198

89	H	4.9945	-0.6746	-3.7906
90	H	3.4752	1.9095	-4.274
91	C	2.9174	0.1333	-5.3559
92	C	2.9385	-1.2897	-5.4719
93	C	2.172	0.8576	-6.334
94	C	2.2753	-1.9272	-6.5132
95	H	3.451	-1.8805	-4.7194
96	C	1.5176	0.2079	-7.3709
97	H	2.1257	1.9401	-6.2559
98	C	1.564	-1.1907	-7.4734
99	H	2.3047	-3.0125	-6.5791
100	H	0.9574	0.7873	-8.1006
101	H	1.0455	-1.6993	-8.2814
102	C	5.6189	1.1535	-2.8654
103	H	6.2952	0.6554	-2.1632
104	H	6.2068	1.4916	-3.7285
105	H	5.1868	2.0245	-2.3662

i5-Cl-trans-s-1

	Atom	X	Y	Z
1	C	0.6089	-0.2211	0.0877
2	C	0.9505	-0.9819	-1.173
3	C	1.8168	-0.1116	0.9998
4	C	0.0993	1.1455	-0.3217
5	C	-0.5275	1.5051	-1.4991
6	H	-0.749	0.9656	-2.406
7	C	0.2691	-2.0136	-1.7772
8	H	-0.606	-2.5757	-1.4955
9	C	1.9549	-0.5215	2.3074
10	H	1.2822	-0.9977	3.002
11	N	3.2295	-0.1941	2.6342
12	N	-0.8231	2.8122	-1.3363
13	N	0.9485	-2.2299	-2.9326
14	N	0.1438	2.2393	0.4901
15	N	-0.4129	3.2502	-0.1248
16	N	1.9794	-1.3809	-3.0532
17	N	1.9816	-0.6251	-1.9865
18	N	3.0083	0.4171	0.6089
19	N	3.8661	0.3658	1.5969
20	Cu	3.7684	0.6937	-1.4127
21	O	-0.3918	-1.0177	0.738
22	C	-1.2823	-0.3402	1.626
23	H	-2.0121	0.2704	1.0799
24	H	-1.8098	-1.1294	2.1686
25	H	-0.7544	0.3	2.3419
26	C	0.576	-3.0722	-4.067
27	H	1.5017	-3.2616	-4.6183
28	H	0.2107	-4.0244	-3.6755
29	C	-1.5309	3.7146	-2.2494
30	H	-2.6018	3.4972	-2.1905
31	H	-1.3671	4.7182	-1.8466
32	C	3.9345	-0.4301	3.8901
33	H	4.6868	0.3586	3.9748
34	H	3.2115	-0.2838	4.698
35	C	-0.4652	-2.4082	-4.9525
36	C	-0.4227	-1.0307	-5.213
37	C	-1.4666	-3.1854	-5.5451
38	C	-1.3704	-0.4432	-6.0534
39	H	0.3507	-0.4151	-4.764
40	C	-2.4104	-2.5983	-6.3927
41	H	-1.5097	-4.253	-5.342
42	C	-2.3656	-1.2254	-6.6471
43	H	-1.3278	0.6259	-6.2418
44	H	-3.1838	-3.2131	-6.8456
45	H	-3.1029	-0.7671	-7.3011
46	C	4.5839	-1.8012	3.9874
47	C	5.3493	-2.084	5.1276
48	C	4.4603	-2.7692	2.987
49	C	5.9849	-3.3184	5.2643
50	H	5.4525	-1.3314	5.9066
51	C	5.0949	-4.0075	3.1277
52	H	3.9067	-2.558	2.0783
53	C	5.8583	-4.2867	4.2617
54	H	6.5791	-3.5244	6.1509
55	H	5.0055	-4.7431	2.3334
56	H	6.3578	-5.2464	4.3634
57	C	-1.0418	3.6063	-3.6783
58	C	-1.9621	3.4683	-4.7232
59	C	0.3278	3.6758	-3.9712
60	C	-1.524	3.4158	-6.0502
61	H	-3.0245	3.4055	-4.5002
62	C	0.7645	3.6175	-5.295
63	H	1.0525	3.7541	-3.165
64	C	-0.159	3.4904	-6.339
65	H	-2.2484	3.3125	-6.8539
66	H	1.8277	3.6749	-5.5118
67	H	0.1841	3.4489	-7.3694
68	N	5.3531	-0.5119	-1.2939
69	S	5.1682	-2.1264	-1.0355
70	O	3.9172	-2.3439	-0.2808
71	O	5.3747	-2.9513	-2.2525
72	C	6.5432	-2.4933	0.0659
73	C	6.8732	-1.5978	1.0896
74	C	7.2116	-3.7106	-0.0623

75	C	7.8806	-1.9361	1.9876
76	H	6.3393	-0.6582	1.1796
77	C	8.2139	-4.0378	0.8538
78	H	6.9525	-4.3899	-0.8677
79	C	8.5594	-3.1624	1.8917
80	H	8.1325	-1.2479	2.7908
81	H	8.733	-4.9884	0.7589
82	C	9.6211	-3.5205	2.9017
83	H	9.1898	-3.5842	3.9089
84	H	10.0897	-4.4831	2.6729
85	H	10.4085	-2.7574	2.9368
86	Cl	3.2131	2.9472	-1.7003
87	C	5.1857	0.6856	-3.1687
88	C	6.2264	-0.1004	-2.4115
89	H	6.5998	-0.949	-2.9915
90	H	5.211	1.7645	-3.0342
91	C	4.4178	0.1969	-4.2846
92	C	4.5887	-1.0999	-4.8255
93	C	3.4765	1.0686	-4.8895
94	C	3.8765	-1.4867	-5.9548
95	H	5.256	-1.8036	-4.3436
96	C	2.756	0.6669	-6.0051
97	H	3.3101	2.0441	-4.4429
98	C	2.9581	-0.6105	-6.5449
99	H	4.0215	-2.4807	-6.3686
100	H	2.0255	1.3366	-6.4479
101	H	2.3885	-0.9253	-7.415
102	C	7.3889	0.7504	-1.9187
103	H	7.016	1.5897	-1.3203
104	H	8.0566	0.1486	-1.2934
105	H	7.9621	1.1486	-2.7655

MECP5trans

	Atom	X	Y	Z
1	C	-0.7706	0.0473	-0.0212
2	C	-0.342	-0.7143	-1.2548
3	C	0.3603	0.1016	0.9845
4	C	-1.155	1.439	-0.4863
5	C	-1.6202	1.8194	-1.7302
6	H	-1.7818	1.2804	-2.6512
7	C	-1.0373	-1.6424	-1.9917
8	H	-1.9807	-2.1388	-1.8365
9	C	0.4475	-0.4222	2.2546
10	H	-0.249	-0.9658	2.8729
11	N	1.6994	-0.1006	2.666
12	N	-1.83	3.1478	-1.6178
13	N	-0.2724	-1.8367	-3.0974
14	N	-1.106	2.5456	0.3094
15	N	-1.5084	3.5827	-0.3783
16	N	0.8283	-1.0787	-3.0684
17	N	0.7861	-0.399	-1.9501
18	N	1.5536	0.6875	0.6968
19	N	2.3667	0.5666	1.718
20	Cu	2.345	0.8826	-1.3402
21	O	-1.8655	-0.6954	0.5177
22	C	-2.7611	0.0238	1.3689
23	H	-3.3878	0.721	0.7992
24	H	-3.397	-0.7347	1.8321
25	H	-2.2333	0.5811	2.1514
26	C	-0.6672	-2.489	-4.3491
27	H	0.254	-2.6306	-4.9201
28	H	-1.0867	-3.4676	-4.1061
29	C	-2.2076	4.0911	-2.6708
30	H	-3.1828	3.7956	-3.0675
31	H	-2.3223	5.0572	-2.171
32	C	2.3628	-0.4398	3.9248
33	H	2.8056	0.4789	4.3178
34	H	1.5716	-0.749	4.6139
35	C	-1.6642	-1.625	-5.1013
36	C	-1.4209	-0.2539	-5.2748
37	C	-2.8314	-2.1846	-5.6294
38	C	-2.334	0.5427	-5.966
39	H	-0.5129	0.1891	-4.8776
40	C	-3.743	-1.3872	-6.3296
41	H	-3.0318	-3.2445	-5.4924
42	C	-3.4985	-0.0226	-6.496
43	H	-2.1274	1.6016	-6.094
44	H	-4.6455	-1.8337	-6.7382
45	H	-4.2099	0.5982	-7.0349
46	C	3.4151	-1.5245	3.7734
47	C	4.4444	-1.5952	4.7209
48	C	3.3729	-2.4604	2.7348
49	C	5.4137	-2.5977	4.6386
50	H	4.4907	-0.8601	5.5214
51	C	4.3464	-3.4598	2.6507
52	H	2.6164	-2.3936	1.96
53	C	5.3661	-3.5336	3.6008
54	H	6.2105	-2.6388	5.3769
55	H	4.3162	-4.1637	1.8236
56	H	6.1293	-4.3039	3.5256
57	C	-1.1671	4.1535	-3.7726
58	C	-1.5641	4.2428	-5.1116
59	C	0.1984	4.1423	-3.4594
60	C	-0.6078	4.3334	-6.1279

61	H	-2.6232	4.2426	-5.3605
62	C	1.1523	4.2301	-4.473
63	H	0.5289	4.0503	-2.4292
64	C	0.7536	4.3292	-5.81
65	H	-0.9261	4.4012	-7.1646
66	H	2.2055	4.2099	-4.2067
67	H	1.4984	4.393	-6.5988
68	N	3.9438	-0.1866	-1.9005
69	S	4.0571	-1.7474	-1.3829
70	O	2.881	-2.0252	-0.5302
71	O	4.3694	-2.7114	-2.4659
72	C	5.4952	-1.6607	-0.314
73	C	5.4556	-0.769	0.7658
74	C	6.6236	-2.438	-0.5648
75	C	6.5591	-0.6735	1.6043
76	H	4.5687	-0.1742	0.9515
77	C	7.7244	-2.3302	0.2916
78	H	6.6379	-3.1181	-1.4101
79	C	7.7083	-1.4539	1.3836
80	H	6.5268	0.0064	2.4519
81	H	8.6059	-2.9391	0.1054
82	C	8.8786	-1.3521	2.3296
83	H	8.5876	-1.6684	3.3398
84	H	9.7155	-1.9798	2.0076
85	H	9.2342	-0.3171	2.4084
86	Cl	2.9594	3.1536	-1.4096
87	C	3.7948	0.7993	-4.0546
88	C	4.7613	0.1894	-3.0793
89	H	5.2203	-0.7027	-3.5174
90	H	3.5657	1.8494	-3.8881
91	C	3.1119	0.1307	-5.0941
92	C	3.254	-1.2643	-5.3551
93	C	2.2238	0.8756	-5.9266
94	C	2.5563	-1.8608	-6.394
95	H	3.8751	-1.87	-4.7038
96	C	1.5344	0.2678	-6.9631
97	H	2.0846	1.9338	-5.7255
98	C	1.6938	-1.1055	-7.2058
99	H	2.6688	-2.9273	-6.5745
100	H	0.8555	0.8537	-7.5773
101	H	1.1423	-1.5844	-8.0096
102	C	5.8548	1.1774	-2.6673
103	H	6.5485	0.699	-1.9678
104	H	6.4158	1.5124	-3.5493
105	H	5.4063	2.0473	-2.178

MECP5_+

	Atom	X	Y	Z
1	C	0.1818	0.0104	0.0672
2	C	0.3812	-0.8796	-1.1479
3	C	1.3227	-0.2468	1.0446
4	C	0.2109	1.4384	-0.4738
5	C	-0.7753	2.3897	-0.6209
6	H	-1.8035	2.4399	-0.3026
7	C	-0.4332	-1.8211	-1.7268
8	H	-1.4027	-2.2109	-1.4623
9	C	1.3796	-1.0806	2.143
10	H	0.6419	-1.69	2.6403
11	N	2.6671	-0.9968	2.5599
12	N	-0.1858	3.3876	-1.3274
13	N	0.2481	-2.2153	-2.8348
14	N	1.3166	1.9152	-1.1132
15	N	1.0785	3.097	-1.63
16	N	1.4083	-1.5663	-2.9578
17	N	1.4892	-0.7579	-1.9318
18	N	2.5661	0.2688	0.8491
19	N	3.3786	-0.1795	1.7717
20	Cu	2.9405	0.7106	-1.5039
21	O	-1.0656	-0.354	0.6068
22	C	-1.52	0.3544	1.7732
23	H	-2.2667	1.1043	1.4931
24	H	-1.9873	-0.3832	2.431
25	H	-0.7002	0.8415	2.3097
26	C	-0.2294	-3.0667	-3.9272
27	H	0.6566	-3.3209	-4.5155
28	H	-0.6271	-3.9848	-3.489
29	C	-0.814	4.5926	-1.9002
30	H	-1.6881	4.8075	-1.2824
31	H	-0.0967	5.4078	-1.7908
32	C	3.313	-1.6787	3.6885
33	H	3.6377	-0.916	4.4012
34	H	2.5295	-2.2726	4.1678
35	C	-1.2784	-2.36	-4.7684
36	C	-1.2067	-0.9812	-5.0078
37	C	-2.3213	-3.1017	-5.3346
38	C	-2.1667	-0.3545	-5.8048
39	H	-0.401	-0.3934	-4.5789
40	C	-3.2777	-2.4754	-6.138
41	H	-2.3857	-4.1707	-5.1457
42	C	-3.2044	-1.0996	-6.3725
43	H	-2.1005	0.7161	-5.9785
44	H	-4.0833	-3.0614	-6.5721
45	H	-3.9529	-0.6111	-6.9906
46	C	4.4799	-2.5481	3.2589

47	C	5.5772	-2.6886	4.117
48	C	4.4713	-3.2343	2.039
49	C	6.6484	-3.5141	3.7657
50	H	5.596	-2.1489	5.0612
51	C	5.5472	-4.0493	1.6833
52	H	3.6502	-3.103	1.3429
53	C	6.6361	-4.1962	2.5463
54	H	7.4953	-3.6135	4.4394
55	H	5.5382	-4.5556	0.7224
56	H	7.4758	-4.8254	2.2643
57	C	-1.187	4.3681	-3.3502
58	C	-2.335	3.6341	-3.6761
59	C	-0.3699	4.8601	-4.3748
60	C	-2.6651	3.3984	-5.012
61	H	-2.9722	3.2493	-2.8833
62	C	-0.7034	4.6312	-5.7124
63	H	0.5222	5.4286	-4.1246
64	C	-1.8495	3.8978	-6.0327
65	H	-3.5603	2.8331	-5.2556
66	H	-0.0682	5.0237	-6.5015
67	H	-2.1101	3.7217	-7.0727
68	N	4.7515	0.2284	-1.8562
69	S	5.2449	-1.3647	-1.6589
70	O	4.1724	-2.0237	-0.8933
71	O	5.6569	-1.9566	-2.95
72	C	6.6945	-1.2487	-0.6222
73	C	6.5491	-0.7724	0.6864
74	C	7.9263	-1.6928	-1.1011
75	C	7.6613	-0.7554	1.5201
76	H	5.5803	-0.4495	1.0513
77	C	9.0287	-1.673	-0.2446
78	H	8.0155	-2.0583	-2.1186
79	C	8.9135	-1.2147	1.0742
80	H	7.5552	-0.4032	2.5423
81	H	9.9896	-2.0298	-0.6049
82	C	10.0923	-1.2341	2.0142
83	H	9.9187	-1.9431	2.8343
84	H	11.0127	-1.5298	1.501
85	H	10.2534	-0.2502	2.4711
86	C	4.4084	1.876	-3.629
87	C	5.42	1.0074	-2.9364
88	H	5.923	0.3389	-3.6428
89	H	4.329	2.9166	-3.3256
90	C	3.474	1.3683	-4.5691
91	C	3.526	0.0221	-5.044
92	C	2.4256	2.201	-5.0686
93	C	2.616	-0.4362	-5.9871
94	H	4.2764	-0.6583	-4.6561
95	C	1.5119	1.722	-5.9948
96	H	2.3448	3.2208	-4.7008
97	C	1.6078	0.4054	-6.4757
98	H	2.68	-1.4634	-6.3364
99	H	0.7133	2.3699	-6.3442
100	H	0.8944	0.0385	-7.2072
101	H	6.1863	1.6324	-2.462

MECP5_Cl

	Atom	X	Y	Z
1	C	-1.6807	1.559	0.0729
2	C	-1.2656	0.8614	-1.2044
3	C	-0.5212	1.648	1.0487
4	C	-2.1941	2.9307	-0.3157
5	C	-2.8071	3.2979	-1.4982
6	H	-3.0139	2.7652	-2.4123
7	C	-1.8879	-0.1644	-1.875
8	H	-2.7558	-0.7607	-1.6475
9	C	-0.4133	1.1573	2.3312
10	H	-1.0913	0.6168	2.9716
11	N	0.8404	1.4987	2.7201
12	N	-3.1062	4.6027	-1.3298
13	N	-1.1644	-0.313	-3.0147
14	N	-2.1618	4.0175	0.5057
15	N	-2.7116	5.0325	-0.1099
16	N	-0.1615	0.5626	-3.075
17	N	-0.2243	1.2751	-1.9788
18	N	0.6633	2.2356	0.7331
19	N	1.4917	2.1439	1.7423
20	Cu	1.3606	2.4807	-1.3933
21	O	-2.6974	0.7185	0.6308
22	C	-3.6274	1.3472	1.5158
23	H	-4.3491	1.9655	0.968
24	H	-4.1569	0.5309	2.014
25	H	-3.1305	1.9691	2.2691
26	C	-1.4661	-1.1437	-4.1826
27	H	-0.5308	-1.2145	-4.7438
28	H	-1.734	-2.1399	-3.8254
29	C	-3.7994	5.5097	-2.2507
30	H	-4.8746	5.3193	-2.1788
31	H	-3.6077	6.5157	-1.8671
32	C	1.5148	1.2064	3.9811
33	H	2.2271	2.0192	4.1458
34	H	0.7593	1.2651	4.7702
35	C	-2.5744	-0.538	-5.023
36	C	-2.5725	0.8315	-5.3258

37	C	-3.5974	-1.351	-5.522
38	C	-3.5856	1.3771	-6.1154
39	H	-1.7796	1.4716	-4.9494
40	C	-4.6068	-0.8056	-6.3208
41	H	-3.6045	-2.4127	-5.2864
42	C	-4.6046	0.5598	-6.6156
43	H	-3.5758	2.4397	-6.3378
44	H	-5.3972	-1.4461	-6.703
45	H	-5.3936	0.9866	-7.2293
46	C	2.2224	-0.1387	4.0058
47	C	3.036	-0.4309	5.1094
48	C	2.096	-1.0796	2.9799
49	C	3.7126	-1.6486	5.1869
50	H	3.1434	0.3009	5.9074
51	C	2.7748	-2.2994	3.0594
52	H	1.5061	-0.8603	2.0965
53	C	3.5829	-2.5892	4.159
54	H	4.3426	-1.8618	6.0467
55	H	2.6826	-3.0125	2.2449
56	H	4.1148	-3.5351	4.2138
57	C	-3.3269	5.3602	-3.6818
58	C	-4.2625	5.2533	-4.7168
59	C	-1.9582	5.3505	-3.9871
60	C	-3.8393	5.1567	-6.0459
61	H	-5.3246	5.2487	-4.4841
62	C	-1.5364	5.2391	-5.3123
63	H	-1.2233	5.4128	-3.1888
64	C	-2.4748	5.1472	-6.3463
65	H	-4.5751	5.0793	-6.842
66	H	-0.4735	5.2277	-5.5363
67	H	-2.1444	5.0657	-7.3786
68	N	2.8411	1.1905	-1.4965
69	S	2.6463	-0.3997	-1.0678
70	O	1.4303	-0.495	-0.2348
71	O	2.7509	-1.323	-2.2219
72	C	4.0672	-0.7083	-0.0171
73	C	4.3903	0.2068	0.9921
74	C	4.7811	-1.897	-0.1641
75	C	5.4452	-0.0806	1.8519
76	H	3.8116	1.1173	1.1104
77	C	5.8294	-2.1733	0.7161
78	H	4.5174	-2.5947	-0.9517
79	C	6.1768	-1.2747	1.7338
80	H	5.6911	0.6208	2.6452
81	H	6.3817	-3.1037	0.6102
82	C	7.2916	-1.5823	2.7024
83	H	6.9004	-1.6772	3.7235

84	H	7.8019	-2.5166	2.4478
85	H	8.0389	-0.7793	2.7177
86	Cl	0.947	4.7515	-1.6607
87	C	3.1321	2.3264	-3.6329
88	C	3.7957	1.4427	-2.6179
89	H	4.1375	0.5048	-3.0647
90	H	3.1306	3.3936	-3.4246
91	C	2.3443	1.8652	-4.7189
92	C	2.3128	0.5025	-5.1348
93	C	1.5112	2.7865	-5.423
94	C	1.5024	0.0981	-6.1871
95	H	2.9016	-0.2356	-4.6019
96	C	0.6833	2.3644	-6.4524
97	H	1.5067	3.8282	-5.1118
98	C	0.6738	1.0172	-6.8476
99	H	1.4978	-0.9467	-6.4884
100	H	0.0294	3.078	-6.9463
101	H	0.0204	0.6877	-7.6503
102	H	4.6679	1.9539	-2.1925

MECP4+

	Atom	X	Y	Z
1	C	-0.4041	-0.0871	0.002
2	C	0.0373	-0.9326	-1.1748
3	C	0.6605	-0.1356	1.0852
4	C	-0.5161	1.3369	-0.513
5	C	-0.6617	1.7693	-1.8151
6	H	-0.7662	1.2507	-2.7558
7	C	-0.6903	-1.7317	-2.0234
8	H	-1.7196	-2.0511	-2.0191
9	C	0.6091	-0.5163	2.406
10	H	-0.1946	-0.8689	3.0328
11	N	1.8805	-0.3549	2.859
12	N	-0.574	3.114	-1.7338
13	N	0.1871	-2.0752	-3.0025
14	N	-0.3547	2.431	0.2877
15	N	-0.3797	3.5062	-0.4543
16	N	1.386	-1.5358	-2.8052
17	N	1.2926	-0.8412	-1.6949
18	N	1.9523	0.2175	0.8342
19	N	2.6957	0.0777	1.9069
20	Cu	2.8052	0.2538	-0.9547
21	O	-1.6131	-0.6644	0.4661
22	C	-2.4758	0.1964	1.2226
23	H	-2.9499	0.9494	0.5826
24	H	-3.2426	-0.4576	1.6437
25	H	-1.9418	0.7037	2.0334

26	C	-0.108	-2.7568	-4.2761
27	H	0.8524	-3.1099	-4.6572
28	H	-0.7382	-3.6184	-4.0475
29	C	-0.3369	4.0424	-2.85
30	H	-1.1774	3.9741	-3.5445
31	H	-0.3185	5.0428	-2.4119
32	C	2.4466	-0.7429	4.166
33	H	3.2305	-0.0154	4.3846
34	H	1.6476	-0.6314	4.9003
35	C	-0.7897	-1.7978	-5.2314
36	C	-0.0958	-0.6781	-5.7142
37	C	-2.1217	-1.9987	-5.6068
38	C	-0.7309	0.2268	-6.5656
39	H	0.9378	-0.5147	-5.4194
40	C	-2.7557	-1.0946	-6.4647
41	H	-2.6631	-2.8629	-5.2296
42	C	-2.0625	0.0191	-6.9434
43	H	-0.1857	1.089	-6.94
44	H	-3.7898	-1.2605	-6.7541
45	H	-2.5553	0.7228	-7.6088
46	C	2.9812	-2.1589	4.1232
47	C	2.291	-3.193	4.7651
48	C	4.1618	-2.4467	3.4221
49	C	2.7751	-4.503	4.7127
50	H	1.3781	-2.9718	5.3124
51	C	4.642	-3.7556	3.3671
52	H	4.7023	-1.6478	2.9212
53	C	3.9495	-4.7862	4.0121
54	H	2.2356	-5.2984	5.2193
55	H	5.5595	-3.9695	2.8264
56	H	4.3258	-5.8047	3.9698
57	C	0.9744	3.6721	-3.5142
58	C	0.997	3.1111	-4.795
59	C	2.169	3.7812	-2.7867
60	C	2.2021	2.6633	-5.3468
61	H	0.0705	3.0095	-5.3545
62	C	3.3701	3.333	-3.3351
63	H	2.1497	4.1983	-1.7824
64	C	3.3876	2.7675	-4.6157
65	H	2.2114	2.2286	-6.343
66	H	4.2891	3.4082	-2.7637
67	H	4.3228	2.4073	-5.0346
68	N	4.2746	1.3118	-0.4073
69	S	5.2681	0.7112	-1.5017
70	O	4.1537	-0.0431	-2.3058
71	O	6.1091	1.6512	-2.2488
72	C	6.3046	-0.5738	-0.8336
73	C	5.7194	-1.7908	-0.4594
74	C	7.6435	-0.2941	-0.5539
75	C	6.4992	-2.7362	0.1958
76	H	4.6794	-1.9992	-0.692
77	C	8.4031	-1.2532	0.1169
78	H	8.0836	0.6516	-0.8518
79	C	7.8464	-2.4782	0.5085
80	H	6.058	-3.6886	0.4758
81	H	9.4445	-1.0423	0.3422
82	C	8.66	-3.4921	1.2703
83	H	8.5188	-4.5019	0.8687
84	H	9.7276	-3.2548	1.2403
85	H	8.3532	-3.5173	2.3245

MECP4Cl

	Atom	X	Y	Z
1	C	-2.536	1.1916	-0.0005
2	C	-2.0766	0.2672	-1.1074
3	C	-1.8186	0.8412	1.2902
4	C	-2.1851	2.6198	-0.3849
5	C	-1.975	3.1819	-1.6305
6	H	-1.9533	2.7758	-2.6291
7	C	-2.6586	-0.0111	-2.3259
8	H	-3.6059	0.2576	-2.7661
9	C	-2.3048	0.3371	2.4725
10	H	-3.2965	0.065	2.7967
11	N	-1.2116	0.2179	3.2681
12	N	-1.7325	4.4866	-1.3739
13	N	-1.7207	-0.7404	-2.9826
14	N	-2.0483	3.6048	0.5533
15	N	-1.7722	4.7316	-0.0456
16	N	-0.6205	-0.9055	-2.2286
17	N	-0.84	-0.3007	-1.0917
18	N	-0.4705	0.9817	1.4376
19	N	-0.1005	0.6	2.6379
20	Cu	0.8539	1.2875	-0.0716
21	O	-3.9398	0.9681	0.1349
22	C	-4.6868	1.973	0.8283
23	H	-4.779	2.8903	0.2344
24	H	-5.6799	1.5438	0.9827
25	H	-4.2461	2.2253	1.7992
26	C	-1.6494	-1.0198	-4.4233
27	H	-0.9022	-1.8077	-4.5445
28	H	-2.6203	-1.4002	-4.7474
29	C	-1.3973	5.5568	-2.3127
30	H	-2.1895	5.6132	-3.0643
31	H	-1.4252	6.4778	-1.7228

32	C	-1.1407	-0.3384	4.6276
33	H	-0.1495	-0.0719	5.0016
34	H	-1.892	0.1734	5.233
35	C	-1.2688	0.254	-5.1573
36	C	-0.0701	0.9106	-4.836
37	C	-2.1374	0.8248	-6.0925
38	C	0.2419	2.1315	-5.4334
39	H	0.6104	0.4777	-4.108
40	C	-1.816	2.0418	-6.7033
41	H	-3.0699	0.3225	-6.3385
42	C	-0.6313	2.7006	-6.3675
43	H	1.1581	2.6461	-5.1588
44	H	-2.498	2.4776	-7.4287
45	H	-0.3883	3.6554	-6.8252
46	C	-1.361	-1.8363	4.6197
47	C	-2.4662	-2.3944	5.2701
48	C	-0.4647	-2.6731	3.9395
49	C	-2.6731	-3.777	5.2487
50	H	-3.1648	-1.7469	5.7947
51	C	-0.6738	-4.0519	3.9139
52	H	0.392	-2.2409	3.4279
53	C	-1.7779	-4.6071	4.5709
54	H	-3.5345	-4.2017	5.7575
55	H	0.0253	-4.6939	3.3847
56	H	-1.9388	-5.6818	4.552
57	C	-0.0435	5.3665	-2.967
58	C	0.1422	5.7744	-4.293
59	C	1.0351	4.8277	-2.2551
60	C	1.3966	5.6598	-4.8973
61	H	-0.6963	6.1817	-4.8535
62	C	2.2859	4.7041	-2.862
63	H	0.9066	4.4954	-1.229
64	C	2.4714	5.1231	-4.1827
65	H	1.5302	5.9791	-5.9277
66	H	3.1106	4.2785	-2.3004
67	H	3.4458	5.0233	-4.6522
68	N	1.7917	1.2787	-1.6195
69	S	3.2285	0.5966	-1.8272
70	O	3.7422	-0.1897	-0.6877
71	O	2.9043	-0.1022	-3.1079
72	C	4.4916	1.8035	-2.2299
73	C	5.2624	2.3756	-1.2137
74	C	4.6495	2.2032	-3.5583
75	C	6.1888	3.3651	-1.5391
76	H	5.1369	2.0515	-0.1871
77	C	5.5924	3.1832	-3.8673
78	H	4.0486	1.7469	-4.3374
79	C	6.3685	3.7863	-2.867
80	H	6.7848	3.8166	-0.7496
81	H	5.7249	3.4835	-4.9037
82	C	7.3623	4.872	-3.2016
83	H	8.3028	4.7341	-2.6559
84	H	7.5884	4.8952	-4.2732
85	H	6.9704	5.8594	-2.9233
86	Cl	2.3008	2.849	0.6401

TS56-Cl-s-N

	Atom	X	Y	Z
1	C	-0.6619	0.6745	-0.03
2	C	0.6085	1.307	1.9767
3	C	-1.2589	-0.5176	-0.3698
4	H	-1.945	-1.1671	0.1483
5	C	0.89	0.705	3.1822
6	H	0.2701	0.2497	3.9375
7	N	2.2407	0.7752	3.2901
8	N	-0.7837	-0.7822	-1.613
9	N	0.0471	0.177	-2.0313
10	N	0.128	1.0622	-1.0692
11	N	1.7909	1.6877	1.4199
12	N	2.7817	1.3647	2.214
13	Cu	2.1436	2.0423	-0.7065
14	O	-1.7484	0.8251	2.0315
15	C	-2.2953	1.5766	3.1163
16	H	-2.9657	2.3695	2.7622
17	H	-2.8703	0.8611	3.7105
18	H	-1.52	2.0287	3.7453
19	C	-1.0751	-1.9046	-2.4989
20	H	-0.1156	-2.3384	-2.7931
21	H	-1.6097	-2.6467	-1.8986
22	C	3.0956	0.234	4.3413
23	H	3.9857	0.8675	4.3744
24	H	2.5627	0.3641	5.2881
25	C	-1.8803	-1.4978	-3.7197
26	C	-2.719	-0.3775	-3.7122
27	C	-1.7861	-2.2728	-4.8821
28	C	-3.4524	-0.0374	-4.8515
29	H	-2.7913	0.2415	-2.8222
30	C	-2.5243	-1.938	-6.0193
31	H	-1.125	-3.1363	-4.8988
32	C	-3.3596	-0.8167	-6.0079
33	H	-4.0932	0.8402	-4.8349
34	H	-2.439	-2.5462	-6.916
35	H	-3.9296	-0.5507	-6.894
36	C	3.4806	-1.2226	4.1334

37	C	4.4132	-1.7873	5.015
38	C	2.9525	-2.0046	3.1027
39	C	4.8105	-3.1167	4.8684
40	H	4.8336	-1.1807	5.8144
41	C	3.3505	-3.3372	2.9582
42	H	2.2651	-1.578	2.3808
43	C	4.2785	-3.8977	3.8362
44	H	5.5374	-3.5411	5.5561
45	H	2.9466	-3.926	2.1393
46	H	4.5927	-4.931	3.7159
47	N	3.4755	0.5358	-1.1819
48	S	3.026	-1.0354	-0.9249
49	O	1.9567	-1.0486	0.0928
50	O	2.7894	-1.8087	-2.1701
51	C	4.5053	-1.7241	-0.1658
52	C	5.1356	-1.0235	0.8695
53	C	4.9594	-2.9817	-0.5601
54	C	6.2264	-1.601	1.5107
55	H	4.7637	-0.0508	1.1735
56	C	6.0506	-3.5502	0.1013
57	H	4.4628	-3.5071	-1.3692
58	C	6.6948	-2.8748	1.1463
59	H	6.7119	-1.0664	2.3235
60	H	6.4024	-4.5343	-0.1987
61	C	7.8503	-3.4983	1.8887
62	H	7.5771	-3.6905	2.9344
63	H	8.1538	-4.4493	1.4395
64	H	8.7219	-2.8324	1.9021
65	C	3.2405	1.6837	-3.0066
66	C	4.2544	0.7662	-2.4125
67	H	4.4487	-0.1335	-3.0005
68	H	3.3323	2.732	-2.7338
69	C	2.1984	1.3465	-3.917
70	C	1.202	2.3241	-4.1759
71	C	2.1207	0.0935	-4.5746
72	C	0.1665	2.0566	-5.0582
73	H	1.2421	3.2662	-3.6368
74	C	1.0907	-0.157	-5.469
75	H	2.8485	-0.6769	-4.3554
76	C	0.109	0.8153	-5.7051
77	H	-0.6056	2.8003	-5.2291
78	H	1.0288	-1.1182	-5.9699
79	H	-0.7143	0.594	-6.3777
80	H	5.2	1.268	-2.189
81	C	-0.7228	1.4609	1.2587
82	C	-1.0211	2.9098	0.9472
83	C	-1.6774	3.4384	-0.147
84	N	-0.7085	3.9341	1.7925
85	H	-2.0621	2.9981	-1.0525
86	N	-1.7243	4.7651	0.0962
87	N	-1.1333	5.0588	1.2761
88	C	-2.3465	5.8223	-0.7101
89	H	-3.4285	5.7854	-0.5507
90	H	-1.9724	6.7612	-0.2933
91	C	-2.0139	5.6944	-2.181
92	C	-3.0335	5.5569	-3.1294
93	C	-0.6778	5.7189	-2.6052
94	C	-2.7259	5.462	-4.4906
95	H	-4.0708	5.5275	-2.8041
96	C	-0.3709	5.624	-3.9624
97	H	0.123	5.7859	-1.8741
98	C	-1.3942	5.4992	-4.9097
99	H	-3.5257	5.3589	-5.2193
100	H	0.668	5.6457	-4.2799
101	H	-1.1521	5.4298	-5.9671
102	Cl	2.3581	4.3865	-0.9849

TS45-Cl-t-2

	Atom	X	Y	Z
1	C	-0.0833	-0.4179	-0.0005
2	C	0.2673	-1.2262	-1.2293
3	C	1.0246	-0.5081	1.028
4	C	-0.25	1.0195	-0.464
5	C	-0.5177	1.4896	-1.7359
6	H	-0.6468	1.0028	-2.6906
7	C	-0.5663	-1.9102	-2.0824
8	H	-1.6151	-2.155	-2.0363
9	C	1.0388	-1.07	2.285
10	H	0.2837	-1.5648	2.8743
11	N	2.3077	-0.8781	2.7262
12	N	-0.564	2.8316	-1.5946
13	N	0.2272	-2.2388	-3.1348
14	N	-0.1452	2.0914	0.3743
15	N	-0.33	3.1886	-0.3115
16	N	1.4732	-1.7938	-2.9674
17	N	1.4995	-1.1798	-1.8081
18	N	2.2729	-0.0269	0.7784
19	N	3.0491	-0.2464	1.8151
20	Cu	3.2412	-0.112	-1.1911
21	O	-1.2792	-1.0058	0.5083
22	C	-2.0608	-0.1961	1.3922
23	H	-2.5741	0.6092	0.8533
24	H	-2.8034	-0.8701	1.8264
25	H	-1.4579	0.2436	2.1948

26	C	-0.1947	-2.7059	-4.4633
27	H	0.7115	-3.0601	-4.9605
28	H	-0.8792	-3.5454	-4.3271
29	C	-0.6935	3.8459	-2.6391
30	H	-1.5867	3.6187	-3.2273
31	H	-0.8643	4.7899	-2.1133
32	C	2.912	-1.2925	4.002
33	H	3.0657	-0.3976	4.6105
34	H	2.1626	-1.9162	4.4976
35	C	-0.8481	-1.5607	-5.2154
36	C	-0.1413	-0.3648	-5.4131
37	C	-2.1648	-1.6651	-5.6741
38	C	-0.7503	0.7139	-6.0552
39	H	0.8848	-0.2779	-5.0682
40	C	-2.7721	-0.5867	-6.3259
41	H	-2.7183	-2.5878	-5.5173
42	C	-2.0685	0.6054	-6.5124
43	H	-0.1924	1.6352	-6.198
44	H	-3.7961	-0.6775	-6.6785
45	H	-2.5431	1.4459	-7.0118
46	C	4.2143	-2.0376	3.7907
47	C	5.3383	-1.7069	4.5551
48	C	4.3101	-3.0569	2.8343
49	C	6.5417	-2.3954	4.375
50	H	5.2756	-0.905	5.2867
51	C	5.5142	-3.7348	2.6454
52	H	3.4553	-3.2997	2.2094
53	C	6.6327	-3.4084	3.4182
54	H	7.4101	-2.1267	4.9708
55	H	5.5812	-4.4991	1.8773
56	H	7.5729	-3.9308	3.2638
57	C	0.5353	3.923	-3.5267
58	C	0.3873	4.2264	-4.8857
59	C	1.8196	3.73	-3.0017
60	C	1.5104	4.3552	-5.7076
61	H	-0.6082	4.3646	-5.3013
62	C	2.9408	3.851	-3.8243
63	H	1.9535	3.4789	-1.9531
64	C	2.7906	4.1713	-5.1771
65	H	1.3842	4.5936	-6.7604
66	H	3.9264	3.6875	-3.3991
67	H	3.6651	4.2704	-5.8145
68	N	4.3334	-1.3608	-2.2549
69	S	5.323	-2.4418	-1.5134
70	O	4.456	-3.2139	-0.581
71	O	6.0632	-3.212	-2.5416
72	C	6.5498	-1.5954	-0.5198
73	C	6.1392	-0.9242	0.6356
74	C	7.8935	-1.6186	-0.8948
75	C	7.0884	-0.2849	1.4242
76	H	5.0961	-0.9054	0.9253
77	C	8.8351	-0.9733	-0.09
78	H	8.1958	-2.1439	-1.7944
79	C	8.4505	-0.304	1.0802
80	H	6.7681	0.2279	2.3273
81	H	9.8845	-0.9966	-0.3736
82	C	9.466	0.3634	1.9741
83	H	9.5439	-0.1655	2.9336
84	H	10.4607	0.3789	1.5173
85	H	9.1769	1.3966	2.2012
86	Cl	4.5195	1.7891	-1.0956
87	C	4.8062	0.4729	-4.475
88	C	5.7607	-0.1494	-3.7357
89	H	6.151	-1.1244	-4.0042
90	H	4.5463	1.499	-4.2253
91	C	4.0302	-0.1465	-5.5449
92	C	3.9294	-1.5482	-5.6787
93	C	3.3325	0.6656	-6.4629
94	C	3.1829	-2.1085	-6.7132
95	H	4.4105	-2.1888	-4.9467
96	C	2.5947	0.1015	-7.5023
97	H	3.3786	1.7457	-6.3548
98	C	2.5181	-1.2886	-7.6335
99	H	3.1115	-3.19	-6.7985
100	H	2.0688	0.7448	-8.2029
101	H	1.9336	-1.7304	-8.4361
102	H	6.2689	0.3864	-2.9423

TS45-Cl-s-c

	Atom	X	Y	Z
1	C	-2.6161	2.9681	0.061
2	C	-2.0693	2.2215	-1.134
3	C	-1.8066	2.6236	1.2963
4	C	-2.5155	4.4529	-0.2333
5	C	-2.4936	5.0968	-1.4565
6	H	-2.4633	4.742	-2.4749
7	C	-2.7287	1.6434	-2.1945
8	H	-3.7737	1.4876	-2.4077
9	C	-2.1891	2.0823	2.5014
10	H	-3.1417	1.7657	2.8943
11	N	-1.0369	1.9907	3.2129
12	N	-2.481	6.41	-1.1423
13	N	-1.7332	1.2624	-3.0364
14	N	-2.5042	5.4	0.7487

15	N	-2.4826	6.5852	0.1974
16	N	-0.5306	1.5819	-2.5525
17	N	-0.737	2.1633	-1.3992
18	N	-0.4593	2.8118	1.3472
19	N	0.01	2.4239	2.51
20	Cu	0.8211	3.1573	-0.2442
21	O	-3.9586	2.5063	0.208
22	C	-4.8561	3.3444	0.943
23	H	-5.1446	4.2308	0.3653
24	H	-5.7415	2.7306	1.1265
25	H	-4.4351	3.6695	1.9008
26	C	-1.8456	0.7736	-4.4203
27	H	-1.1389	-0.0498	-4.5381
28	H	-2.8615	0.3851	-4.5265
29	C	-2.4537	7.5669	-2.0453
30	H	-3.3097	7.4862	-2.7198
31	H	-2.604	8.4364	-1.4007
32	C	-0.8336	1.3945	4.5403
33	H	0.146	1.7472	4.8726
34	H	-1.5951	1.8035	5.2077
35	C	-1.5577	1.8998	-5.3936
36	C	-0.4247	1.8603	-6.2123
37	C	-2.3971	3.0226	-5.4352
38	C	-0.132	2.9306	-7.0632
39	H	0.2376	0.9997	-6.1728
40	C	-2.0977	4.097	-6.2734
41	H	-3.2815	3.0572	-4.8024
42	C	-0.9617	4.0534	-7.0887
43	H	0.7532	2.8917	-7.6918
44	H	-2.7502	4.9659	-6.2931
45	H	-0.7246	4.892	-7.7376
46	C	-0.8987	-0.1181	4.4834
47	C	-1.8004	-0.8188	5.2907
48	C	-0.0514	-0.826	3.6189
49	C	-1.8527	-2.2153	5.2416
50	H	-2.4618	-0.2725	5.9591
51	C	-0.109	-2.2185	3.5647
52	H	0.6491	-0.2842	2.9874
53	C	-1.0093	-2.9166	4.3775
54	H	-2.5555	-2.7512	5.874
55	H	0.5503	-2.759	2.8909
56	H	-1.0519	-4.0017	4.3355
57	C	-1.1582	7.6684	-2.8197
58	C	-1.1478	7.5155	-4.2101
59	C	0.0436	7.9285	-2.1451
60	C	0.0483	7.6353	-4.9245
61	H	-2.077	7.3054	-4.735
62	C	1.2354	8.0519	-2.858
63	H	0.0394	8.0319	-1.0625
64	C	1.2388	7.9103	-4.2501
65	H	0.0486	7.507	-6.0034
66	H	2.164	8.2475	-2.3306
67	H	2.1715	7.9924	-4.7996
68	N	1.9792	3.1591	-1.7368
69	S	3.1437	2.0588	-1.5656
70	O	2.9366	1.3419	-0.2693
71	O	3.187	1.2533	-2.8112
72	C	4.7621	2.832	-1.4406
73	C	5.1732	3.3803	-0.2244
74	C	5.573	2.9283	-2.5753
75	C	6.4037	4.0341	-0.1519
76	H	4.5352	3.3029	0.6482
77	C	6.8006	3.5798	-2.4849
78	H	5.2416	2.4994	-3.514
79	C	7.2342	4.1459	-1.2761
80	H	6.7241	4.463	0.7945
81	H	7.4276	3.6605	-3.3694
82	C	8.5612	4.8614	-1.2036
83	H	9.3933	4.1795	-1.4213
84	H	8.6105	5.6713	-1.9422
85	H	8.732	5.2956	-0.2132
86	Cl	2.0465	4.806	0.7206
87	C	1.2296	4.4041	-4.0533
88	H	0.4082	3.7471	-4.3169
89	C	2.3863	4.3717	-4.7392
90	H	2.4695	3.6906	-5.586
91	H	1.0602	5.093	-3.2346
92	C	3.5765	5.1841	-4.461
93	C	3.7825	5.819	-3.2184
94	C	4.5613	5.3354	-5.456
95	C	4.9121	6.6033	-2.9993
96	H	3.0702	5.6652	-2.4158
97	C	5.693	6.1219	-5.2356
98	H	4.4285	4.8359	-6.4134
99	C	5.8695	6.7665	-4.0079
100	H	5.0575	7.0718	-2.0294
101	H	6.4371	6.23	-6.021
102	H	6.7518	7.3763	-3.8309

TS45-Cl-oss-2

	Atom	X	Y	Z
1	C	0.3779	-1.1625	0.0034
2	C	0.7345	-1.9844	-1.2149
3	C	1.482	-1.244	1.0362

4	C	0.2143	0.2705	-0.4744	51	C	5.9026	-4.5168	2.6332
5	C	-0.0496	0.7304	-1.7508	52	H	3.8533	-4.0241	2.2091
6	H	-0.1726	0.2363	-2.7026	53	C	7.0309	-4.2278	3.4069
7	C	-0.0991	-2.6684	-2.0672	54	H	7.8454	-2.982	4.9694
8	H	-1.1507	-2.9014	-2.0258	55	H	5.9481	-5.2771	1.8594
9	C	1.4933	-1.7847	2.3022	56	H	7.9563	-4.7739	3.246
10	H	0.7352	-2.2639	2.9004	57	C	0.9997	3.1653	-3.5494
11	N	2.765	-1.5975	2.7378	58	C	0.8588	3.4361	-4.9159
12	N	-0.1009	2.0733	-1.6189	59	C	2.2822	3.0086	-3.0085
13	N	0.697	-3.0115	-3.1129	60	C	1.9873	3.5675	-5.7302
14	N	0.3131	1.3486	0.3567	61	H	-0.1352	3.5462	-5.3433
15	N	0.1271	2.4401	-0.3376	62	C	3.4087	3.1329	-3.823
16	N	1.9452	-2.5775	-2.9438	63	H	2.4098	2.7822	-1.9535
17	N	1.9705	-1.9543	-1.7884	64	C	3.2654	3.4189	-5.1842
18	N	2.7343	-0.7795	0.778	65	H	1.8667	3.7797	-6.7893
19	N	3.5113	-0.9886	1.8165	66	H	4.3934	2.9968	-3.3862
20	Cu	3.6788	-0.8694	-1.1745	67	H	4.1442	3.5186	-5.8156
21	O	-0.8199	-1.7445	0.5127	68	N	4.79	-2.0633	-2.2754
22	C	-1.6051	-0.9258	1.3858	69	S	5.8117	-3.14	-1.6065
23	H	-2.1151	-0.1253	0.8372	70	O	4.9643	-3.9947	-0.7253
24	H	-2.35	-1.5955	1.8225	71	O	6.5877	-3.8229	-2.6692
25	H	-1.0053	-0.4792	2.1869	72	C	7.0099	-2.3215	-0.5549
26	C	0.2734	-3.4797	-4.4416	73	C	6.5694	-1.7062	0.6202
27	H	1.1784	-3.836	-4.9389	74	C	8.3601	-2.3076	-0.9057
28	H	-0.4139	-4.3165	-4.3032	75	C	7.4933	-1.0848	1.4519
29	C	-0.2341	3.081	-2.67	76	H	5.5214	-1.7143	0.8913
30	H	-1.121	2.8413	-3.2624	77	C	9.2771	-1.6827	-0.0571
31	H	-0.4178	4.0258	-2.1501	78	H	8.6861	-2.7869	-1.8225
32	C	3.3724	-2.011	4.0127	79	C	8.8614	-1.0688	1.1323
33	H	3.5569	-1.1125	4.6069	80	H	7.148	-0.6146	2.3688
34	H	2.6124	-2.6081	4.5249	81	H	10.3315	-1.6776	-0.3229
35	C	-0.3767	-2.3306	-5.1896	82	C	9.8496	-0.419	2.0691
36	C	0.3506	-1.1533	-5.4219	83	H	9.8993	-0.9651	3.0206
37	C	-1.711	-2.4079	-5.6007	84	H	10.8574	-0.3943	1.6425
38	C	-0.2563	-0.0654	-6.0503	85	H	9.5535	0.6102	2.3062
39	H	1.3906	-1.0885	-5.1157	86	Cl	4.9592	1.0379	-1.0674
40	C	-2.3163	-1.3202	-6.2387	87	C	5.2441	-0.1314	-4.6415
41	H	-2.2794	-3.3166	-5.4172	88	C	6.2523	-0.678	-3.9328
42	C	-1.5924	-0.1461	-6.4585	89	H	6.6241	-1.6783	-4.1277
43	H	0.3176	0.841	-6.2214	90	H	4.9657	0.9013	-4.4421
44	H	-3.3539	-1.3897	-6.5545	91	C	4.4434	-0.8194	-5.659
45	H	-2.0649	0.702	-6.9471	92	C	4.3267	-2.2239	-5.6936
46	C	4.6521	-2.7933	3.7951	93	C	3.7501	-0.0663	-6.6274
47	C	5.7857	-2.5007	4.5606	94	C	3.5711	-2.8474	-6.6859
48	C	4.7179	-3.8081	2.8309	95	H	4.8057	-2.8161	-4.9208
49	C	6.9691	-3.2215	4.3729	96	C	3.0012	-0.6929	-7.623
50	H	5.7471	-1.7037	5.2991	97	H	3.8103	1.0184	-6.5966

98	C	2.9106	-2.0875	-7.6585
99	H	3.4899	-3.9316	-6.6961
100	H	2.4787	-0.0935	-8.364
101	H	2.3183	-2.5777	-8.4266
102	H	6.7614	-0.1024	-3.1673

TS34-Cl

	Atom	X	Y	Z
1	C	-1.9304	-0.9384	-0.0075
2	C	-0.8419	-1.1025	-1.045
3	C	-1.3775	-0.7233	1.3862
4	C	-2.7937	0.2376	-0.4259
5	C	-3.1416	0.6483	-1.6996
6	H	-2.8349	0.3322	-2.6845
7	C	-0.8925	-1.8574	-2.196
8	H	-1.6189	-2.5593	-2.5712
9	C	-1.7391	-1.3446	2.5607
10	H	-2.4436	-2.1266	2.7909
11	N	-0.9802	-0.7431	3.509
12	N	-4.0054	1.6672	-1.4987
13	N	0.2291	-1.5179	-2.8759
14	N	-3.4575	1.0206	0.4717
15	N	-4.1873	1.8894	-0.1772
16	N	0.9476	-0.6121	-2.2113
17	N	0.2993	-0.3641	-1.0968
18	N	-0.4222	0.2007	1.6837
19	N	-0.1747	0.1826	2.9732
20	Cu	1.0454	1.0831	0.144
21	O	-2.645	-2.1844	-0.0425
22	C	-4.0345	-2.1514	0.2936
23	H	-4.6308	-1.6762	-0.4947
24	H	-4.3342	-3.1985	0.3872
25	H	-4.2269	-1.6349	1.2406
26	C	0.5676	-1.835	-4.2658
27	H	1.6185	-1.559	-4.3881
28	H	0.4736	-2.9145	-4.402
29	C	-4.7228	2.4744	-2.4866
30	H	-5.4152	1.8211	-3.026
31	H	-5.3185	3.1794	-1.8989
32	C	-0.9088	-1.0586	4.9455
33	H	-0.5556	-0.1526	5.4402
34	H	-1.9308	-1.2633	5.2735
35	C	-0.3365	-1.0739	-5.2189
36	C	-0.6262	0.2802	-4.9937
37	C	-0.8948	-1.7214	-6.3256
38	C	-1.4709	0.972	-5.862
39	H	-0.2027	0.7953	-4.1352
40	C	-1.7325	-1.0242	-7.2021
41	H	-0.6789	-2.7727	-6.5006
42	C	-2.0259	0.3213	-6.969
43	H	-1.6937	2.0169	-5.6725
44	H	-2.1631	-1.5372	-8.058
45	H	-2.6858	0.8623	-7.6421
46	C	0.0092	-2.2331	5.2057
47	C	-0.4375	-3.542	4.9755
48	C	1.3326	-2.0205	5.607
49	C	0.4341	-4.6217	5.1259
50	H	-1.4676	-3.7134	4.6721
51	C	2.2049	-3.101	5.7629
52	H	1.6838	-1.0075	5.7804
53	C	1.759	-4.4016	5.517
54	H	0.0808	-5.6325	4.9408
55	H	3.2329	-2.9241	6.0664
56	H	2.4392	-5.2413	5.6317
57	C	-3.811	3.2041	-3.4534
58	C	-4.254	3.4473	-4.7596
59	C	-2.5577	3.6827	-3.0537
60	C	-3.4631	4.1784	-5.6497
61	H	-5.2217	3.0676	-5.0797
62	C	-1.759	4.3973	-3.9491
63	H	-2.1929	3.4893	-2.0499
64	C	-2.2119	4.6547	-5.2465
65	H	-3.8187	4.3646	-6.6597
66	H	-0.7834	4.7509	-3.6274
67	H	-1.591	5.2143	-5.9409
68	N	2.3069	1.8067	1.287
69	I	3.5559	-0.6081	2.8248
70	S	3.6556	2.6072	0.8043
71	C	2.6488	-1.908	1.4342
72	O	3.2678	3.905	0.214
73	O	4.5954	2.6197	1.9461
74	C	4.4339	1.6375	-0.4963
75	C	3.1428	-1.9565	0.1264
76	C	1.5736	-2.7095	1.8353
77	C	3.7997	1.5393	-1.7403
78	C	5.6008	0.9216	-0.2332
79	C	2.5544	-2.8357	-0.7868
80	H	3.9678	-1.3231	-0.1796
81	C	0.9923	-3.574	0.9067
82	H	1.1965	-2.6629	2.8492
83	C	4.3328	0.6974	-2.7114
84	H	2.8875	2.0956	-1.9332
85	C	6.1286	0.0876	-1.2234
86	H	6.0811	1.0075	0.735

87	C	1.482	-3.643	-0.4018
88	H	2.9356	-2.8747	-1.803
89	H	0.1507	-4.1901	1.211
90	C	5.501	-0.046	-2.469
91	H	3.8316	0.61	-3.6725
92	H	7.0365	-0.4739	-1.0177
93	H	1.0258	-4.3209	-1.1174
94	C	6.0518	-0.9629	-3.5337
95	H	6.3591	-0.3965	-4.4221
96	H	6.9208	-1.5229	-3.1741
97	H	5.2935	-1.6852	-3.8615
98	Cl	0.3425	2.8894	-1.068

i6-Cl

	Atom	X	Y	Z
1	C	-3.4894	-0.6326	0.5019
2	C	-3.7269	-1.4687	-0.7416
3	C	-2.4125	-1.2881	1.3475
4	C	-3.1114	0.7822	0.1153
5	C	-2.8711	1.3126	-1.1397
6	H	-2.8858	0.8822	-2.1259
7	C	-4.6466	-1.2565	-1.7527
8	H	-5.3576	-0.4683	-1.9448
9	C	-2.5735	-2.3688	2.1869
10	H	-3.4421	-2.942	2.4684
11	N	-1.3304	-2.648	2.6362
12	N	-2.5614	2.6067	-0.907
13	N	-4.5055	-2.3282	-2.5639
14	N	-2.9191	1.7732	1.0347
15	N	-2.5966	2.8781	0.41
16	N	-3.5551	-3.1635	-2.0973
17	N	-3.0856	-2.6433	-0.9907
18	N	-1.0832	-0.9821	1.3302
19	N	-0.426	-1.8126	2.1158
20	Cu	-0.0071	0.3399	0.2592
21	O	-4.752	-0.714	1.1905
22	C	-4.9121	0.026	2.4041
23	H	-5.0526	1.0946	2.2134
24	H	-5.8114	-0.3807	2.8758
25	H	-4.062	-0.1019	3.0851
26	C	-5.1704	-2.6099	-3.8383
27	H	-5.1778	-3.699	-3.9356
28	H	-6.2039	-2.2672	-3.7551
29	C	-2.2027	3.6755	-1.8595
30	H	-3.014	4.4097	-1.8429
31	H	-1.2952	4.1391	-1.4691
32	C	-0.8949	-3.7972	3.4371
33	H	0.0932	-3.5343	3.824
34	H	-1.5812	-3.8956	4.2809
35	C	-4.4736	-1.9635	-5.0202
36	C	-3.1037	-2.1678	-5.2382
37	C	-5.2057	-1.1914	-5.9283
38	C	-2.4801	-1.6101	-6.3542
39	H	-2.5269	-2.7654	-4.5382
40	C	-4.5825	-0.641	-7.0529
41	H	-6.2676	-1.027	-5.7609
42	C	-3.2187	-0.8507	-7.2682
43	H	-1.4184	-1.7772	-6.5115
44	H	-5.1623	-0.0484	-7.7555
45	H	-2.7325	-0.4233	-8.141
46	C	-0.8551	-5.0656	2.6048
47	C	-1.4132	-6.25	3.0965
48	C	-0.2459	-5.0602	1.3427
49	C	-1.3586	-7.4225	2.3359
50	H	-1.8922	-6.2572	4.0728
51	C	-0.1985	-6.2285	0.5817
52	H	0.191	-4.1481	0.9498
53	C	-0.7534	-7.4135	1.0768
54	H	-1.7954	-8.3382	2.7256
55	H	0.2747	-6.2079	-0.3963
56	H	-0.7161	-8.3234	0.4836
57	C	-1.9848	3.129	-3.2491
58	C	-3.0788	2.8496	-4.0807
59	C	-0.6904	2.843	-3.6993
60	C	-2.8827	2.2709	-5.3355
61	H	-4.0858	3.0733	-3.7354
62	C	-0.4921	2.2771	-4.9618
63	H	0.1545	3.0445	-3.0469
64	C	-1.5876	1.9815	-5.7767
65	H	-3.7364	2.037	-5.9648
66	H	0.5168	2.0582	-5.3014
67	H	-1.4367	1.5252	-6.7506
68	N	0.8708	-1.0707	-1.4499
69	S	2.248	-2.0604	-1.1339
70	O	1.7548	-3.2049	-0.3552
71	O	3.0314	-2.2973	-2.3588
72	C	3.1638	-0.9744	-0.0629
73	C	2.777	-0.8658	1.2771
74	C	4.2215	-0.2302	-0.5826
75	C	3.4725	0.012	2.1026
76	H	1.944	-1.4468	1.659
77	C	4.9059	0.6423	0.264
78	H	4.5034	-0.3311	-1.6252
79	C	4.5435	0.7782	1.6115

80	H	3.1777	0.1097	3.1445
81	H	5.7322	1.2279	-0.1301
82	C	5.2939	1.7107	2.5287
83	H	5.9224	1.1451	3.2293
84	H	5.9451	2.3891	1.9685
85	H	4.6037	2.3137	3.13
86	Cl	0.8888	2.3219	-0.3378
87	C	0.8103	-0.3732	-2.7533
88	C	-0.1174	-1.5025	-2.5006
89	H	-1.119	-1.2605	-2.1522
90	H	1.6416	-0.5469	-3.4317
91	C	-0.016	-2.8145	-3.2137
92	C	-0.4469	-3.9811	-2.565
93	C	0.4418	-2.8926	-4.5345
94	C	-0.4143	-5.207	-3.2293
95	H	-0.8102	-3.922	-1.5453
96	C	0.4692	-4.1201	-5.2012
97	H	0.7709	-1.9923	-5.0447
98	C	0.0421	-5.2796	-4.5494
99	H	-0.7514	-6.1036	-2.7161
100	H	0.8225	-4.1682	-6.2277
101	H	0.0626	-6.2349	-5.0674
102	H	0.4708	0.652	-2.6676

i5-Cl-t

	Atom	X	Y	Z
1	C	-0.935	-0.1414	0.0953
2	C	-0.5434	-0.9473	-1.1239
3	C	0.2219	-0.0778	1.0721
4	C	-1.2672	1.2548	-0.4039
5	C	-1.6371	1.645	-1.6768
6	H	-1.7729	1.106	-2.6019
7	C	-1.3056	-1.7895	-1.8982
8	H	-2.3019	-2.1847	-1.7831
9	C	0.358	-0.5541	2.356
10	H	-0.3149	-1.0735	3.0193
11	N	1.6267	-0.2201	2.7083
12	N	-1.7822	2.9842	-1.5883
13	N	-0.5235	-2.0511	-2.9784
14	N	-1.2049	2.368	0.3834
15	N	-1.5103	3.4157	-0.3358
16	N	0.6479	-1.4191	-2.9007
17	N	0.6358	-0.7502	-1.7761
18	N	1.4065	0.4943	0.7264
19	N	2.2609	0.4102	1.7196
20	Cu	2.2332	0.503	-1.2155
21	O	-2.0358	-0.8346	0.6749
22	C	-2.8772	-0.0729	1.5465
23	H	-3.4781	0.6564	0.9903
24	H	-3.5408	-0.7995	2.0213
25	H	-2.3057	0.4547	2.3188
26	C	-0.9266	-2.6508	-4.2579
27	H	-0.0004	-2.9369	-4.7616
28	H	-1.5067	-3.5497	-4.0411
29	C	-2.0117	3.9385	-2.6717
30	H	-2.9096	3.6329	-3.2152
31	H	-2.2194	4.8949	-2.1829
32	C	2.3473	-0.5354	3.943
33	H	2.7275	0.4046	4.352
34	H	1.6013	-0.9252	4.6414
35	C	-1.7246	-1.6445	-5.0676
36	C	-1.176	-0.3844	-5.3513
37	C	-3.0166	-1.9467	-5.5088
38	C	-1.9169	0.5619	-6.0599
39	H	-0.1695	-0.1447	-5.0222
40	C	-3.7553	-1.0014	-6.2282
41	H	-3.4481	-2.92	-5.2873
42	C	-3.2094	0.2552	-6.4997
43	H	-1.4798	1.5345	-6.2689
44	H	-4.7585	-1.2461	-6.5667
45	H	-3.7865	0.9924	-7.0518
46	C	3.4794	-1.5276	3.7315
47	C	4.4563	-1.6375	4.7301
48	C	3.5709	-2.333	2.5912
49	C	5.5085	-2.5445	4.5932
50	H	4.3969	-1.005	5.6132
51	C	4.6277	-3.2365	2.4538
52	H	2.8517	-2.2452	1.7838
53	C	5.5986	-3.3462	3.4506
54	H	6.2628	-2.6167	5.3726
55	H	4.697	-3.8353	1.5508
56	H	6.4251	-4.042	3.3344
57	C	-0.8188	4.0403	-3.6055
58	C	-1.0264	4.2467	-4.9746
59	C	0.4907	3.9603	-3.1132
60	C	0.0616	4.3939	-5.84
61	H	-2.0407	4.2955	-5.3639
62	C	1.5767	4.0989	-3.9787
63	H	0.6731	3.7843	-2.0565
64	C	1.3659	4.325	-5.3423
65	H	-0.111	4.557	-6.9005
66	H	2.5829	4.0255	-3.5773
67	H	2.2127	4.439	-6.0138
68	N	3.5635	-0.4608	-2.3124

69	S	4.0826	-1.8355	-1.6335
70	O	2.9943	-2.3041	-0.738
71	O	4.5855	-2.7834	-2.6578
72	C	5.4861	-1.4777	-0.5601
73	C	5.416	-0.3925	0.3197
74	C	6.5757	-2.348	-0.5284
75	C	6.4404	-0.1943	1.2408
76	H	4.5729	0.289	0.2919
77	C	7.5933	-2.1383	0.4047
78	H	6.6196	-3.1816	-1.2214
79	C	7.537	-1.0689	1.3085
80	H	6.3796	0.6437	1.931
81	H	8.4391	-2.821	0.4343
82	C	8.6035	-0.8731	2.3573
83	H	8.2195	-1.1478	3.3487
84	H	9.4852	-1.4908	2.158
85	H	8.9229	0.1742	2.4142
86	Cl	3.3198	2.536	-0.998
87	C	3.6631	0.8534	-4.3648
88	C	4.4631	0.2229	-3.2636
89	H	5.1659	-0.512	-3.6809
90	H	3.5648	1.9352	-4.3619
91	C	2.9946	0.1282	-5.3827
92	C	3.0301	-1.2967	-5.4651
93	C	2.256	0.8235	-6.3868
94	C	2.3745	-1.9649	-6.4922
95	H	3.554	-1.8634	-4.7028
96	C	1.608	0.1431	-7.4079
97	H	2.2038	1.9077	-6.3386
98	C	1.6598	-1.2578	-7.4715
99	H	2.4147	-3.051	-6.5326
100	H	1.0497	0.6999	-8.1565
101	H	1.1462	-1.7899	-8.2675
102	H	5.0499	0.9895	-2.7461

i5-Cl-t-freq

	Atom	X	Y	Z
1	C	-1.6999	-0.4183	-0.0168
2	C	-1.3084	-1.2242	-1.236
3	C	-0.543	-0.3547	0.96
4	C	-2.0321	0.9779	-0.516
5	C	-2.4021	1.3681	-1.7888
6	H	-2.5378	0.8291	-2.7139
7	C	-2.0705	-2.0664	-2.0102
8	H	-3.0668	-2.4615	-1.8951
9	C	-0.4069	-0.831	2.2439
10	H	-1.0798	-1.3504	2.9072
11	N	0.8618	-0.497	2.5963
12	N	-2.5471	2.7073	-1.7004
13	N	-1.2884	-2.328	-3.0904
14	N	-1.9698	2.0911	0.2713
15	N	-2.2752	3.1388	-0.4479
16	N	-0.117	-1.696	-3.0128
17	N	-0.1291	-1.0271	-1.8882
18	N	0.6415	0.2175	0.6144
19	N	1.496	0.1333	1.6076
20	Cu	1.4683	0.2261	-1.3276
21	O	-2.8007	-1.1115	0.5628
22	C	-3.6421	-0.3498	1.4344
23	H	-4.243	0.3795	0.8782
24	H	-4.3057	-1.0764	1.9093
25	H	-3.0706	0.1778	2.2068
26	C	-1.6915	-2.9277	-4.3699
27	H	-0.7653	-3.2138	-4.8737
28	H	-2.2716	-3.8266	-4.1532
29	C	-2.7766	3.6616	-2.7837
30	H	-3.6745	3.356	-3.3272
31	H	-2.9843	4.618	-2.2949
32	C	1.5824	-0.8123	3.8309
33	H	1.9626	0.1277	4.2399
34	H	0.8364	-1.2021	4.5293
35	C	-2.4895	-1.9214	-5.1796
36	C	-1.9409	-0.6613	-5.4634
37	C	-3.7816	-2.2236	-5.6208
38	C	-2.6818	0.285	-6.1719
39	H	-0.9344	-0.4216	-5.1342
40	C	-4.5202	-1.2783	-6.3403
41	H	-4.2131	-3.1968	-5.3993
42	C	-3.9743	-0.0217	-6.6118
43	H	-2.2447	1.2576	-6.381
44	H	-5.5234	-1.523	-6.6788
45	H	-4.5514	0.7155	-7.1639
46	C	2.7144	-1.8045	3.6194
47	C	3.6914	-1.9144	4.618
48	C	2.806	-2.6099	2.4791
49	C	4.7436	-2.8214	4.4811
50	H	3.632	-1.2819	5.5011
51	C	3.8627	-3.5134	2.3417
52	H	2.0868	-2.5221	1.6717
53	C	4.8336	-3.6231	3.3385
54	H	5.4979	-2.8936	5.2605
55	H	3.9321	-4.1121	1.4387
56	H	5.6602	-4.3189	3.2224
57	C	-1.5837	3.7634	-3.7175

58	C	-1.7913	3.9698	-5.0867
59	C	-0.2742	3.6834	-3.2253
60	C	-0.7034	4.117	-5.952
61	H	-2.8057	4.0186	-5.476
62	C	0.8118	3.8221	-4.0908
63	H	-0.0919	3.5074	-2.1685
64	C	0.601	4.0482	-5.4544
65	H	-0.8759	4.2801	-7.0126
66	H	1.818	3.7486	-3.6894
67	H	1.4478	4.1621	-6.1259
68	N	2.7986	-0.7377	-2.4245
69	S	3.3177	-2.1124	-1.7456
70	O	2.2294	-2.581	-0.8501
71	O	3.8206	-3.0603	-2.7699
72	C	4.7211	-1.7546	-0.6721
73	C	4.651	-0.6694	0.2077
74	C	5.8108	-2.6249	-0.6405
75	C	5.6755	-0.4712	1.1287
76	H	3.808	0.0121	0.1798
77	C	6.8284	-2.4152	0.2926
78	H	5.8547	-3.4584	-1.3335
79	C	6.7721	-1.3458	1.1965
80	H	5.6147	0.3668	1.819
81	H	7.6742	-3.0979	0.3223
82	C	7.8386	-1.15	2.2452
83	H	7.4546	-1.4247	3.2367
84	H	8.7203	-1.7677	2.0459
85	H	8.158	-0.1027	2.3022
86	Cl	2.5549	2.2591	-1.11
87	C	2.8981	0.5765	-4.4769
88	C	3.6982	-0.054	-3.3756
89	H	4.401	-0.7889	-3.793
90	H	2.7999	1.6583	-4.474
91	C	2.2297	-0.1487	-5.4948
92	C	2.2652	-1.5736	-5.5772
93	C	1.4911	0.5466	-6.4988
94	C	1.6095	-2.2417	-6.6043
95	H	2.7891	-2.1403	-4.8149
96	C	0.8431	-0.1338	-7.52
97	H	1.4389	1.6308	-6.4506
98	C	0.8949	-1.5347	-7.5836
99	H	1.6498	-3.3279	-6.6447
100	H	0.2848	0.423	-8.2686
101	H	0.3813	-2.0668	-8.3795
102	H	4.285	0.7126	-2.8582

i5-Cl-s

	Atom	X	Y	Z
1	C	0.6109	-0.222	0.0865
2	C	0.9502	-0.9845	-1.1738
3	C	1.8198	-0.1132	0.9971
4	C	0.1028	1.145	-0.3234
5	C	-0.5241	1.5051	-1.5006
6	H	-0.7468	0.9656	-2.4073
7	C	0.2658	-2.0147	-1.7773
8	H	-0.6106	-2.5745	-1.4949
9	C	1.9566	-0.5182	2.3064
10	H	1.2823	-0.9896	3.0027
11	N	3.2321	-0.1933	2.6321
12	N	-0.818	2.8126	-1.3378
13	N	0.9442	-2.2332	-2.9327
14	N	0.1489	2.2389	0.4882
15	N	-0.4068	3.2503	-0.1265
16	N	1.9774	-1.3871	-3.0539
17	N	1.9819	-0.6308	-1.9876
18	N	3.0131	0.4103	0.6043
19	N	3.8705	0.3604	1.5928
20	Cu	3.776	0.6851	-1.4165
21	O	-0.3902	-1.0167	0.7387
22	C	-1.2812	-0.3368	1.6245
23	H	-2.0105	0.2725	1.0764
24	H	-1.8091	-1.1246	2.1686
25	H	-0.7537	0.3051	2.3391
26	C	0.5701	-3.0761	-4.0662
27	H	1.4955	-3.2684	-4.6169
28	H	0.2025	-4.0269	-3.6736
29	C	-1.5247	3.7159	-2.251
30	H	-2.5957	3.4981	-2.194
31	H	-1.3617	4.7191	-1.8467
32	C	3.9364	-0.4267	3.8888
33	H	4.6921	0.3593	3.9694
34	H	3.2145	-0.2737	4.6964
35	C	-0.4691	-2.411	-4.9532
36	C	-0.4257	-1.0334	-5.2128
37	C	-1.4695	-3.1876	-5.5485
38	C	-1.3712	-0.4452	-6.0551
39	H	0.3469	-0.4183	-4.7618
40	C	-2.4111	-2.5998	-6.398
41	H	-1.5133	-4.2553	-5.3461
42	C	-2.3653	-1.2268	-6.6516
43	H	-1.328	0.624	-6.2428
44	H	-3.1836	-3.2141	-6.853
45	H	-3.1008	-0.7679	-7.3071
46	C	4.5804	-1.8	3.9914

47	C	5.3374	-2.0842	5.1369
48	C	4.4618	-2.7676	2.9902
49	C	5.9697	-3.3198	5.2779
50	H	5.4365	-1.3319	5.9168
51	C	5.0931	-4.0071	3.1352
52	H	3.9147	-2.5555	2.0777
53	C	5.8482	-4.2877	4.2744
54	H	6.5574	-3.527	6.1686
55	H	5.008	-4.7424	2.3401
56	H	6.3453	-5.2484	4.3794
57	C	-1.0333	3.6093	-3.6792
58	C	-1.9515	3.4697	-4.7256
59	C	0.3367	3.6817	-3.9698
60	C	-1.511	3.4181	-6.0519
61	H	-3.0142	3.4047	-4.5044
62	C	0.7758	3.6243	-5.2927
63	H	1.0598	3.7612	-3.1622
64	C	-0.1457	3.4954	-6.3383
65	H	-2.2338	3.3135	-6.8568
66	H	1.8392	3.6835	-5.5078
67	H	0.1992	3.4546	-7.3681
68	N	5.3622	-0.5201	-1.2943
69	S	5.1796	-2.1327	-1.0369
70	O	3.9292	-2.352	-0.2817
71	O	5.3883	-2.9567	-2.2544
72	C	6.5539	-2.4975	0.0656
73	C	6.8757	-1.6026	1.0924
74	C	7.2289	-3.7112	-0.0626
75	C	7.8797	-1.9388	1.995
76	H	6.3349	-0.6668	1.1837
77	C	8.2279	-4.0363	0.8578
78	H	6.9762	-4.3901	-0.8705
79	C	8.5639	-3.162	1.8999
80	H	8.124	-1.2519	2.8016
81	H	8.7513	-4.9846	0.7637
82	C	9.6211	-3.5185	2.9153
83	H	9.1851	-3.5814	3.9205
84	H	10.0915		-4.481
	2.6899			
85	H	10.408	-2.755	2.9533
86	Cl	3.2199	2.9368	-1.7016
87	C	5.1868	0.6898	-3.1783
88	C	6.2135	-0.1129	-2.4242
89	H	6.6251	-0.9452	-3.0012
90	H	5.2197	1.7681	-3.0507
91	C	4.4101	0.1982	-4.2857
92	C	4.576	-1.1022	-4.8202
93	C	3.4705	1.0701	-4.8936
94	C	3.8631	-1.4914	-5.9479
95	H	5.2407	-1.8062	-4.3346
96	C	2.7488	0.6658	-6.0071
97	H	3.3073	2.0481	-4.4515
98	C	2.9476	-0.6146	-6.5418
99	H	4.0047	-2.4876	-6.3575
100	H	2.0201	1.3355	-6.4527
101	H	2.3773	-0.9311	-7.4107
102	H	7.0389	0.5149	-2.0737

i5-Cl-s-freq

	Atom	X	Y	Z
1	C	-1.6818	1.5273	0
2	C	-1.3425	0.7648	-1.2603
3	C	-0.4729	1.6361	0.9107
4	C	-2.1899	2.8942	-0.4099
5	C	-2.8167	3.2543	-1.5871
6	H	-3.0395	2.7149	-2.4938
7	C	-2.0269	-0.2654	-1.8638
8	H	-2.9032	-0.8252	-1.5814
9	C	-0.3361	1.2311	2.2199
10	H	-1.0103	0.7597	2.9163
11	N	0.9394	1.556	2.5457
12	N	-3.1107	4.5619	-1.4243
13	N	-1.3485	-0.4839	-3.0192
14	N	-2.1438	3.9881	0.4018
15	N	-2.6994	4.9996	-0.213
16	N	-0.3152	0.3622	-3.1403
17	N	-0.3107	1.1184	-2.0741
18	N	0.7204	2.1595	0.5179
19	N	1.5778	2.1097	1.5063
20	Cu	1.4833	2.4344	-1.5029
21	O	-2.6828	0.7326	0.6523
22	C	-3.5738	1.4125	1.538
23	H	-4.3031	2.0218	0.99
24	H	-4.1018	0.6246	2.0822
25	H	-3.0464	2.0544	2.2527
26	C	-1.7225	-1.3268	-4.1527
27	H	-0.7971	-1.5191	-4.7034
28	H	-2.0902	-2.2777	-3.7601
29	C	-3.8174	5.4652	-2.3374
30	H	-4.8883	5.2474	-2.2804
31	H	-3.6544	6.4683	-1.9332
32	C	1.6438	1.3226	3.8023
33	H	2.3995	2.1086	3.883
34	H	0.9219	1.4755	4.6099

35	C	-2.7618	-0.6618	-5.0396
36	C	-2.7183	0.7158	-5.2993
37	C	-3.7621	-1.4384	-5.6349
38	C	-3.6639	1.3041	-6.1415
39	H	-1.9457	1.3309	-4.8482
40	C	-4.7037	-0.8506	-6.4844
41	H	-3.806	-2.5061	-5.4325
42	C	-4.6579	0.5225	-6.7381
43	H	-3.6207	2.3733	-6.3293
44	H	-5.4763	-1.4649	-6.9395
45	H	-5.3935	0.9814	-7.3935
46	C	2.2877	-0.0507	3.905
47	C	3.0447	-0.335	5.0505
48	C	2.1691	-1.0184	2.9037
49	C	3.6771	-1.5706	5.1915
50	H	3.1438	0.4173	5.8303
51	C	2.8005	-2.2578	3.0488
52	H	1.622	-0.8063	1.9913
53	C	3.5555	-2.5385	4.1879
54	H	4.2647	-1.7777	6.0821
55	H	2.7153	-2.9931	2.2536
56	H	4.0526	-3.4991	4.2929
57	C	-3.3259	5.3586	-3.7656
58	C	-4.2442	5.2189	-4.8121
59	C	-1.956	5.4309	-4.0562
60	C	-3.8037	5.1674	-6.1383
61	H	-5.3069	5.154	-4.5908
62	C	-1.5168	5.3735	-5.3792
63	H	-1.2329	5.5105	-3.2487
64	C	-2.4384	5.2447	-6.4247
65	H	-4.5265	5.0628	-6.9432
66	H	-0.4534	5.4328	-5.5943
67	H	-2.0935	5.2039	-7.4546
68	N	3.0695	1.2292	-1.3807
69	S	2.8869	-0.3834	-1.1234
70	O	1.6366	-0.6027	-0.3681
71	O	3.0956	-1.2074	-2.3408
72	C	4.2612	-0.7482	-0.0208
73	C	4.583	0.1467	1.006
74	C	4.9362	-1.962	-0.149
75	C	5.5871	-0.1896	1.9085
76	H	4.0423	1.0824	1.0973
77	C	5.9352	-2.287	0.7713
78	H	4.6836	-2.6408	-0.957
79	C	6.2713	-1.4128	1.8135
80	H	5.8314	0.4974	2.7151
81	H	6.4586	-3.2353	0.6772
82	C	7.3285	-1.7693	2.8289
83	H	6.8925	-1.8321	3.8341
84	H	7.7988	-2.7318	2.6035
85	H	8.1153	-1.0057	2.8669
86	Cl	0.9273	4.6861	-1.788
87	C	2.8941	2.4391	-3.2648
88	C	3.9209	1.6364	-2.5107
89	H	4.3325	0.8041	-3.0877
90	H	2.9271	3.5174	-3.1371
91	C	2.1175	1.9475	-4.3722
92	C	2.2834	0.647	-4.9067
93	C	1.1778	2.8194	-4.9801
94	C	1.5704	0.2578	-6.0344
95	H	2.948	-0.0569	-4.4211
96	C	0.4561	2.415	-6.0935
97	H	1.0147	3.7974	-4.538
98	C	0.6549	1.1347	-6.6283
99	H	1.7121	-0.7383	-6.4439
100	H	-0.2726	3.0848	-6.5391
101	H	0.0847	0.8181	-7.4972
102	H	4.7462	2.2642	-2.1601

i4-Cl-t

	Atom	X	Y	Z
1	C	-1.3079	1.4143	-0.0604
2	C	-0.7412	0.4895	-1.1162
3	C	-0.8849	0.9157	1.3103
4	C	-0.7208	2.7939	-0.2906
5	C	-0.0998	3.293	-1.4187
6	H	0.1307	2.8689	-2.3808
7	C	-1.3249	-0.0481	-2.2379
8	H	-2.3279	-0.0025	-2.6292
9	C	-1.6157	0.5665	2.4211
10	H	-2.6734	0.5555	2.6294
11	N	-0.6835	0.1942	3.3358
12	N	0.2846	4.536	-1.0647
13	N	-0.3135	-0.7053	-2.8622
14	N	-0.6834	3.7526	0.6813
15	N	-0.0747	4.8089	0.2103
16	N	0.8356	-0.5892	-2.1963
17	N	0.574	0.1367	-1.1352
18	N	0.4284	0.724	1.6136
19	N	0.5527	0.2809	2.8423
20	Cu	1.9833	0.8827	0.2209
21	O	-2.7217	1.3286	-0.205
22	C	-3.4904	2.424	0.302
23	H	-3.3648	3.3225	-0.3132

24	H	-4.5313	2.0948	0.2543
25	H	-3.2328	2.6676	1.3389
26	C	-0.3199	-1.2957	-4.21
27	H	0.5627	-1.9373	-4.2579
28	H	-1.2141	-1.9161	-4.2965
29	C	1.1082	5.4827	-1.8279
30	H	1.042	6.4294	-1.2858
31	H	2.1396	5.1253	-1.7933
32	C	-0.9024	-0.3491	4.6849
33	H	0.0343	-0.1899	5.2238
34	H	-1.6813	0.2546	5.155
35	C	-0.2908	-0.2015	-5.2583
36	C	0.7872	0.6952	-5.2935
37	C	-1.3467	-0.0524	-6.1628
38	C	0.7971	1.7375	-6.2197
39	H	1.6126	0.586	-4.5946
40	C	-1.3294	0.9884	-7.0973
41	H	-2.1842	-0.7456	-6.1345
42	C	-0.2609	1.887	-7.1234
43	H	1.6301	2.4339	-6.2313
44	H	-2.1533	1.0988	-7.7975
45	H	-0.2525	2.7029	-7.8414
46	C	-1.2869	-1.8131	4.641
47	C	-2.5511	-2.2269	5.0731
48	C	-0.376	-2.7641	4.1595
49	C	-2.9034	-3.5793	5.0296
50	H	-3.2597	-1.4911	5.4462
51	C	-0.7293	-4.1127	4.1107
52	H	0.6073	-2.4441	3.8229
53	C	-1.994	-4.523	4.5475
54	H	-3.8869	-3.8921	5.37
55	H	-0.0175	-4.8439	3.7372
56	H	-2.2669	-5.5744	4.5121
57	C	0.6333	5.6194	-3.2569
58	C	-0.6165	6.1918	-3.5342
59	C	1.4327	5.1689	-4.3132
60	C	-1.0584	6.3158	-4.8514
61	H	-1.2412	6.5365	-2.7131
62	C	0.9948	5.3039	-5.6342
63	H	2.3859	4.6962	-4.0962
64	C	-0.2502	5.8751	-5.9056
65	H	-2.0285	6.7608	-5.0573
66	H	1.6249	4.9575	-6.4489
67	H	-0.5919	5.9751	-6.9325
68	N	3.4723	0.6642	-1.0275
69	S	3.6064	1.6323	-2.3589
70	O	3.6956	0.745	-3.5453
71	O	2.6103	2.7305	-2.4358
72	C	5.1971	2.3921	-2.0789
73	C	6.3459	1.8033	-2.6115
74	C	5.2673	3.5667	-1.3209
75	C	7.5806	2.4127	-2.3917
76	H	6.2691	0.8935	-3.1979
77	C	6.5088	4.157	-1.109
78	H	4.3659	3.996	-0.8982
79	C	7.6821	3.5933	-1.6406
80	H	8.4786	1.9654	-2.8099
81	H	6.5737	5.0695	-0.5215
82	C	9.0153	4.2596	-1.4091
83	H	9.075	5.2123	-1.9516
84	H	9.1638	4.486	-0.3465
85	H	9.8452	3.6299	-1.7448
86	Cl	3.2632	2.3378	1.5424

i4-Cl-t-freq

	Atom	X	Y	Z
1	C	0.8125	-0.4286	0
2	C	1.3792	-1.3534	-1.0558
3	C	1.2355	-0.9272	1.3706
4	C	1.3996	0.951	-0.2302
5	C	2.0206	1.4502	-1.3583
6	H	2.251	1.026	-2.3204
7	C	0.7955	-1.891	-2.1775
8	H	-0.2075	-1.8454	-2.5688
9	C	0.5047	-1.2764	2.4815
10	H	-0.5531	-1.2874	2.6898
11	N	1.4368	-1.6487	3.3962
12	N	2.405	2.6931	-1.0043
13	N	1.8068	-2.5482	-2.8018
14	N	1.437	1.9097	0.7417
15	N	2.0457	2.966	0.2707
16	N	2.956	-2.4321	-2.1359
17	N	2.6943	-1.7062	-1.0748
18	N	2.5488	-1.1189	1.674
19	N	2.673	-1.562	2.9027
20	Cu	4.1036	-0.9602	0.2812
21	O	-0.6013	-0.5143	-0.1446
22	C	-1.37	0.5811	0.3624
23	H	-1.2445	1.4796	-0.2528
24	H	-2.411	0.2519	0.3147
25	H	-1.1124	0.8247	1.3992
26	C	1.8004	-3.1386	-4.1496
27	H	2.6831	-3.7802	-4.1975
28	H	0.9062	-3.759	-4.2361

29	C	3.2286	3.6398	-1.7675
30	H	3.1624	4.5865	-1.2254
31	H	4.2599	3.2824	-1.7329
32	C	1.2179	-2.192	4.7453
33	H	2.1547	-2.0328	5.2842
34	H	0.4391	-1.5883	5.2154
35	C	1.8296	-2.0444	-5.1979
36	C	2.9076	-1.1477	-5.2331
37	C	0.7737	-1.8953	-6.1024
38	C	2.9175	-0.1054	-6.1593
39	H	3.7329	-1.2569	-4.5342
40	C	0.791	-0.8545	-7.0369
41	H	-0.0639	-2.5885	-6.0741
42	C	1.8595	0.0442	-7.063
43	H	3.7505	0.591	-6.1709
44	H	-0.0329	-0.7441	-7.7371
45	H	1.8679	0.86	-7.781
46	C	0.8335	-3.656	4.7014
47	C	-0.4307	-4.0698	5.1335
48	C	1.7444	-4.607	4.2198
49	C	-0.783	-5.4221	5.09
50	H	-1.1393	-3.334	5.5066
51	C	1.391	-5.9555	4.1711
52	H	2.7277	-4.287	3.8833
53	C	0.1263	-6.3659	4.6079
54	H	-1.7665	-5.735	5.4304
55	H	2.1028	-6.6868	3.7976
56	H	-0.1465	-7.4173	4.5725
57	C	2.7537	3.7765	-3.1965
58	C	1.5039	4.3489	-3.4738
59	C	3.5531	3.326	-4.2528
60	C	1.0619	4.473	-4.791
61	H	0.8792	4.6936	-2.6527
62	C	3.1151	3.461	-5.5738
63	H	4.5063	2.8534	-4.0358
64	C	1.8702	4.0323	-5.8452
65	H	0.0919	4.9179	-4.997
66	H	3.7453	3.1146	-6.3885
67	H	1.5285	4.1323	-6.8721
68	N	5.5927	-1.1787	-0.9671
69	S	5.7268	-0.2106	-2.2985
70	O	5.8159	-1.0979	-3.4849
71	O	4.7307	0.8877	-2.3754
72	C	7.3175	0.5492	-2.0185
73	C	8.4663	-0.0396	-2.5512
74	C	7.3876	1.7238	-1.2606
75	C	9.701	0.5698	-2.3314
76	H	8.3895	-0.9494	-3.1375
77	C	8.6292	2.3142	-1.0486
78	H	6.4863	2.1532	-0.8378
79	C	9.8024	1.7504	-1.5802
80	H	10.599	0.1225	-2.7496
81	H	8.6941	3.2266	-0.4611
82	C	11.1356	2.4167	-1.3487
83	H	11.1953	3.3695	-1.8912
84	H	11.2842	2.6431	-0.2861
85	H	11.9655	1.787	-1.6844
86	Cl	5.3836	0.4949	1.6028

i4-Cl-s

	Atom	X	Y	Z
1	C	-2.7305	-0.3077	-0.1564
2	C	-1.6344	-0.2949	-1.1995
3	C	-2.1189	-0.1369	1.2203
4	C	-3.6604	0.8457	-0.4818
5	C	-3.9636	1.3687	-1.7245
6	H	-3.6283	1.1317	-2.7219
7	C	-1.4141	-1.1249	-2.2725
8	H	-1.9028	-2.0228	-2.6137
9	C	-2.0963	-0.9887	2.3027
10	H	-2.5212	-1.964	2.4775
11	N	-1.3631	-0.3264	3.2337
12	N	-4.8051	2.3907	-1.4612
13	N	-0.3751	-0.5501	-2.9339
14	N	-4.3349	1.5626	0.4613
15	N	-5.0247	2.5026	-0.1315
16	N	0.0288	0.5702	-2.3334
17	N	-0.7333	0.7239	-1.277
18	N	-1.393	0.9655	1.5509
19	N	-0.9319	0.8479	2.7717
20	Cu	-0.305	2.2347	0.115
21	O	-3.3429	-1.5907	-0.2631
22	C	-4.6586	-1.7038	0.2882
23	H	-5.4003	-1.1889	-0.3342
24	H	-4.8808	-2.7737	0.3023
25	H	-4.7156	-1.3083	1.309
26	C	0.1539	-0.8927	-4.26
27	H	1.1319	-0.4096	-4.3242
28	H	0.2968	-1.9745	-4.2945
29	C	-5.4147	3.3374	-2.3987
30	H	-6.2359	2.8339	-2.9176
31	H	-5.8447	4.126	-1.774
32	C	-0.8771	-0.8084	4.539
33	H	-0.965	0.0274	5.236

34	H	-1.5562	-1.6033	4.8523
35	C	-0.7876	-0.4181	-5.351
36	C	-1.2141	0.9181	-5.382
37	C	-1.2474	-1.3097	-6.3254
38	C	-2.0931	1.3516	-6.3744
39	H	-0.8652	1.6176	-4.6262
40	C	-2.1204	-0.872	-7.3265
41	H	-0.9243	-2.3477	-6.3019
42	C	-2.5472	0.4572	-7.3501
43	H	-2.4218	2.3865	-6.3832
44	H	-2.4712	-1.5725	-8.0797
45	H	-3.2314	0.7969	-8.123
46	C	0.5518	-1.2961	4.4266
47	C	0.8232	-2.6494	4.1911
48	C	1.6109	-0.3807	4.4884
49	C	2.1404	-3.0848	4.0228
50	H	0.0034	-3.3624	4.1397
51	C	2.9263	-0.8149	4.3156
52	H	1.4055	0.6749	4.6392
53	C	3.1935	-2.1674	4.0845
54	H	2.3433	-4.1374	3.8438
55	H	3.7374	-0.0933	4.349
56	H	4.2177	-2.5045	3.9487
57	C	-4.4173	3.8981	-3.3938
58	C	-4.8212	4.1287	-4.7142
59	C	-3.1067	4.2185	-3.0126
60	C	-3.9344	4.6857	-5.6395
61	H	-5.8332	3.8726	-5.0197
62	C	-2.2154	4.7581	-3.9412
63	H	-2.7689	4.0375	-1.9958
64	C	-2.6275	4.9988	-5.2558
65	H	-4.2613	4.8629	-6.6608
66	H	-1.2	4.987	-3.6312
67	H	-1.9329	5.4217	-5.9765
68	N	0.7378	3.0748	1.4479
69	S	2.3373	2.8389	1.3501
70	O	3.1658	3.865	0.6895
71	O	2.6009	2.585	2.799
72	C	2.5923	1.3045	0.4512
73	C	3.2946	1.2943	-0.7534
74	C	1.9951	0.1346	0.9425
75	C	3.3895	0.1024	-1.4749
76	H	3.7413	2.2097	-1.1257
77	C	2.1032	-1.0443	0.2144
78	H	1.4419	0.1598	1.8731
79	C	2.7928	-1.0774	-1.0101
80	H	3.924	0.0943	-2.4214
81	H	1.6381	-1.95	0.596
82	C	2.8733	-2.3599	-1.8001
83	H	3.4995	-3.1004	-1.2854
84	H	1.8804	-2.8107	-1.9202
85	H	3.2989	-2.1952	-2.7951
86	Cl	0.1852	3.9868	-1.2053

i3-Cl

	Atom	X	Y	Z
1	C	-2.3436	-0.8072	0.2544
2	C	-1.1323	-1.0905	-0.6037
3	C	-1.9258	-0.6871	1.7071
4	C	-2.9783	0.4689	-0.256
5	C	-2.9818	0.9552	-1.5492
6	H	-2.5165	0.6149	-2.4606
7	C	-0.9762	-1.9789	-1.6414
8	H	-1.6265	-2.7228	-2.0715
9	C	-2.1494	-1.561	2.7489
10	H	-2.7048	-2.4821	2.8246
11	N	-1.5014	-1.0037	3.8023
12	N	-3.7167	2.0851	-1.4814
13	N	0.2795	-1.7377	-2.0987
14	N	-3.7087	1.3168	0.522
15	N	-4.154	2.2981	-0.2194
16	N	0.8695	-0.7546	-1.4069
17	N	0.0127	-0.359	-0.4985
18	N	-1.1462	0.3292	2.1739
19	N	-0.889	0.1357	3.4478
20	Cu	0.3004	1.3505	0.8248
21	O	-3.1849	-1.9554	0.0933
22	C	-4.5557	-1.7882	0.4619
23	H	-5.0929	-1.1555	-0.2549
24	H	-4.9885	-2.7918	0.45
25	H	-4.6673	-1.3591	1.4647
26	C	0.9152	-2.2364	-3.3135
27	H	1.9835	-2.0352	-3.1948
28	H	0.7749	-3.3191	-3.3541
29	C	-4.0792	3.0084	-2.5604
30	H	-4.8623	2.5417	-3.1659
31	H	-4.5144	3.8785	-2.0602
32	C	-1.3082	-1.5527	5.1524
33	H	-1.4707	-0.7388	5.8617
34	H	-2.0897	-2.3025	5.2953
35	C	0.3603	-1.5576	-4.5539
36	C	-0.1088	-0.2376	-4.5105
37	C	0.3547	-2.2474	-5.7719
38	C	-0.5811	0.3798	-5.6704

39	H	-0.0953	0.3179	-3.5775
40	C	-0.1085	-1.6256	-6.9345
41	H	0.7115	-3.2741	-5.8104
42	C	-0.58	-0.311	-6.8853
43	H	-0.9412	1.403	-5.6232
44	H	-0.1085	-2.1714	-7.8743
45	H	-0.9464	0.1717	-7.7875
46	C	0.0771	-2.1469	5.2995
47	C	0.4074	-3.332	4.6289
48	C	1.0572	-1.4852	6.0468
49	C	1.7038	-3.8464	4.6994
50	H	-0.3499	-3.8473	4.0429
51	C	2.353	-2.0037	6.1285
52	H	0.8084	-0.5593	6.5586
53	C	2.6798	-3.1819	5.4516
54	H	1.9522	-4.7613	4.1686
55	H	3.1071	-1.4824	6.7114
56	H	3.6888	-3.5811	5.5076
57	C	-2.8973	3.4033	-3.4229
58	C	-3.0512	3.4956	-4.8114
59	C	-1.6609	3.7218	-2.8452
60	C	-1.9875	3.9174	-5.615
61	H	-4.0059	3.2417	-5.2662
62	C	-0.5977	4.1359	-3.6493
63	H	-1.5179	3.644	-1.7703
64	C	-0.7565	4.2368	-5.0356
65	H	-2.1198	3.987	-6.6915
66	H	0.3523	4.3876	-3.186
67	H	0.0733	4.5599	-5.6587
68	N	2.2546	0.8216	1.5873
69	I	2.4728	-0.9438	2.5565
70	S	3.668	1.4582	0.9244
71	C	2.33	-2.4638	1.0792
72	O	3.6093	2.9069	1.1922
73	O	4.8774	0.7115	1.3411
74	C	3.5102	1.2559	-0.8531
75	C	3.4463	-2.754	0.2904
76	C	1.1235	-3.1549	0.9469
77	C	2.6118	2.0607	-1.5573
78	C	4.2848	0.299	-1.5112
79	C	3.3409	-3.7675	-0.6647
80	H	4.3708	-2.2015	0.4164
81	C	1.0424	-4.1752	-0.0037
82	H	0.2653	-2.8989	1.5591
83	C	2.4967	1.8977	-2.9376
84	H	1.9997	2.7888	-1.0322
85	C	4.1571	0.1518	-2.8918
86	H	4.9846	-0.3103	-0.9505
87	C	2.1453	-4.4787	-0.8073
88	H	4.197	-4.0038	-1.2902
89	H	0.1108	-4.7209	-0.1223
90	C	3.2673	0.9504	-3.6275
91	H	1.7922	2.5154	-3.4871
92	H	4.7664	-0.5869	-3.4082
93	H	2.0736	-5.2715	-1.5467
94	C	3.1625	0.7967	-5.1255
95	H	2.3454	1.3972	-5.5349
96	H	4.093	1.1114	-5.6159
97	H	2.9846	-0.2473	-5.4082
98	Cl	-0.1812	3.6017	0.502

i2-Cl

	Atom	X	Y	Z
1	C	-2.7257	1.5056	0.0991
2	C	-2.5335	0.7031	-1.1731
3	C	-1.6814	1.0964	1.1213
4	C	-2.5572	2.9646	-0.2832
5	C	-2.7572	3.5407	-1.5216
6	H	-3.0755	3.1429	-2.4719
7	C	-3.4551	0.0599	-1.9645
8	H	-4.5124	-0.1184	-1.855
9	C	-1.8231	0.4285	2.3158
10	H	-2.6823	0.0629	2.8549
11	N	-0.5536	0.275	2.7687
12	N	-2.3735	4.8245	-1.3625
13	N	-2.7451	-0.3442	-3.0494
14	N	-2.0816	3.9239	0.5628
15	N	-1.9613	5.0486	-0.0938
16	N	-1.4626	0.0269	-2.961
17	N	-1.3324	0.6639	-1.8193
18	N	-0.3493	1.2954	0.909
19	N	0.3362	0.7915	1.9087
20	Cu	0.3904	1.5653	-1.1411
21	Cl	2.4167	2.4065	-1.637
22	O	-4.0298	1.1707	0.5677
23	C	-4.6149	2.0904	1.4936
24	H	-4.8891	3.0334	1.0053
25	H	-5.5181	1.5998	1.8643
26	H	-3.9486	2.3082	2.3367
27	C	-3.2573	-0.8899	-4.3119
28	H	-2.3958	-1.3326	-4.8182
29	H	-3.9672	-1.6843	-4.0725
30	C	-2.1991	5.8377	-2.4064
31	H	-3.1839	6.1805	-2.7368

32	H	-1.6909	6.6752	-1.9199
33	C	-0.0935	-0.4544	3.9555
34	H	0.9396	-0.1346	4.1145
35	H	-0.6921	-0.123	4.8071
36	C	-3.9046	0.2029	-5.142
37	C	-3.1892	1.3693	-5.4533
38	C	-5.2233	0.0666	-5.5865
39	C	-3.7904	2.3841	-6.198
40	H	-2.1648	1.4863	-5.108
41	C	-5.8224	1.0802	-6.3413
42	H	-5.7841	-0.8321	-5.3409
43	C	-5.1087	2.2412	-6.6448
44	H	-3.2271	3.2844	-6.4262
45	H	-6.8478	0.9641	-6.682
46	H	-5.5759	3.0324	-7.2252
47	C	-0.1915	-1.9549	3.7621
48	C	-0.8728	-2.7462	4.6925
49	C	0.4045	-2.5612	2.6471
50	C	-0.9536	-4.1312	4.5168
51	H	-1.3401	-2.2796	5.5565
52	C	0.3178	-3.9418	2.4679
53	H	0.9319	-1.949	1.9191
54	C	-0.3609	-4.7306	3.4036
55	H	-1.4844	-4.7374	5.2462
56	H	0.7814	-4.403	1.5999
57	H	-0.4267	-5.8062	3.2633
58	C	-1.3983	5.2851	-3.5714
59	C	-1.7933	5.5569	-4.8854
60	C	-0.2669	4.4866	-3.3464
61	C	-1.0638	5.0447	-5.9636
62	H	-2.6761	6.1652	-5.068
63	C	0.4514	3.9617	-4.4208
64	H	0.0519	4.2501	-2.3342
65	C	0.0557	4.2409	-5.7336
66	H	-1.3792	5.2647	-6.9802
67	H	1.3156	3.336	-4.2168
68	H	0.6148	3.8317	-6.5709

i1-Cl-s

	Atom	X	Y	Z
1	C	-0.1134	-0.6311	-0.0367
2	C	0.4698	-1.0809	-1.3597
3	C	0.8343	-0.9538	1.104
4	C	-0.3554	0.8615	-0.1338
5	C	-0.6781	1.5959	-1.258
6	H	-0.7976	1.3291	-2.2955
7	C	-0.0814	-1.866	-2.3461
8	H	-1.0118	-2.4052	-2.4154
9	C	0.6244	-1.7384	2.2152
10	H	-0.2261	-2.3032	2.5585
11	N	1.8033	-1.7007	2.8839
12	N	-0.7779	2.8653	-0.8121
13	N	0.8304	-1.8338	-3.3501
14	N	-0.2902	1.7114	0.9294
15	N	-0.5425	2.9278	0.5178
16	N	1.8834	-1.0688	-3.0264
17	N	1.6663	-0.6157	-1.8151
18	N	2.115	-0.4932	1.1584
19	N	2.7042	-0.9464	2.2411
20	Cu	3.0988	0.3788	-0.5163
21	O	-1.3144	-1.3923	0.1215
22	C	-2.3036	-0.8384	0.9926
23	H	-2.8188	0.0112	0.5278
24	H	-3.0227	-1.6419	1.1714
25	H	-1.881	-0.5132	1.9502
26	C	0.6977	-2.3584	-4.7118
27	H	1.7088	-2.3603	-5.1279
28	H	0.3518	-3.3923	-4.6433
29	C	-1.0743	4.0835	-1.5759
30	H	-2.1605	4.1903	-1.6547
31	H	-0.6951	4.9088	-0.9663
32	C	2.149	-2.3006	4.1816
33	H	3.236	-2.4205	4.1676
34	H	1.8965	-1.5822	4.9667
35	C	-0.25	-1.5119	-5.5411
36	C	-0.1213	-0.1153	-5.5616
37	C	-1.2586	-2.1199	-6.2954
38	C	-0.9941	0.6594	-6.326
39	H	0.6576	0.3669	-4.9758
40	C	-2.1272	-1.3439	-7.0691
41	H	-1.367	-3.2018	-6.2769
42	C	-1.9984	0.0465	-7.0831
43	H	-0.8883	1.7403	-6.3275
44	H	-2.9075	-1.8268	-7.6513
45	H	-2.6778	0.6512	-7.6781
46	C	1.4462	-3.6195	4.4122
47	C	0.7158	-3.8284	5.5869
48	C	1.5361	-4.652	3.467
49	C	0.0899	-5.0565	5.8216
50	H	0.6362	-3.0294	6.32
51	C	0.9048	-5.874	3.6967
52	H	2.0935	-4.4925	2.5486
53	C	0.183	-6.0812	4.8774
54	H	-0.4732	-5.2082	6.7385

55	H	0.9788	-6.6657	2.9557
56	H	-0.3075	-7.0342	5.0567
57	C	-0.434	4.0627	-2.9485
58	C	-1.1928	4.3754	-4.0816
59	C	0.9232	3.7382	-3.0962
60	C	-0.6027	4.3779	-5.3497
61	H	-2.247	4.6196	-3.9738
62	C	1.5067	3.7252	-4.3634
63	H	1.523	3.4826	-2.2252
64	C	0.7471	4.0477	-5.4938
65	H	-1.2006	4.6272	-6.2225
66	H	2.5576	3.4667	-4.465
67	H	1.2046	4.0394	-6.4796
68	Cl	3.492	2.6927	-0.5694
69	C	3.647	-5.1527	0.1777
70	C	4.3836	-4.4743	1.1526
71	C	4.8139	-3.1673	0.9214
72	C	4.5349	-2.5153	-0.2936
73	C	3.7984	-3.2139	-1.2702
74	C	3.3553	-4.5131	-1.0331
75	H	3.3045	-6.1683	0.3578
76	H	4.6166	-4.9599	2.0964
77	H	5.3751	-2.6397	1.6882
78	H	3.5594	-2.7337	-2.2123
79	H	2.7822	-5.0313	-1.7979
80	C	5.0296	-1.1407	-0.4864
81	H	5.5452	-0.7168	0.3748
82	C	4.9698	-0.391	-1.6193
83	H	5.4776	0.5674	-1.6679
84	H	4.5414	-0.763	-2.5462

add-Cl-t

	Atom	X	Y	Z
1	C	0.2922	-1.6669	0.1071
2	C	1.0582	-2.3166	-1.0197
3	C	0.9078	-1.9742	1.46
4	C	0.2523	-0.1723	-0.1445
5	C	0.1482	0.4942	-1.3522
6	H	0.1867	0.1642	-2.3785
7	C	0.5759	-2.8176	-2.2085
8	H	-0.4234	-2.9506	-2.5896
9	C	0.2572	-2.2775	2.6345
10	H	-0.7837	-2.4055	2.8844
11	N	1.2477	-2.4063	3.5488
12	N	-0.0191	1.7883	-1.0064
13	N	1.6888	-3.1462	-2.9112
14	N	0.1486	0.7449	0.859
15	N	-0.0106	1.9338	0.3356
16	N	2.7975	-2.8751	-2.2299
17	N	2.416	-2.3694	-1.0798
18	N	2.247	-1.9562	1.7316
19	N	2.4481	-2.222	3.004
20	Cu	3.8482	-1.892	0.3577
21	O	-1.0136	-2.262	0.0491
22	C	-2.1314	-1.4587	0.4397
23	H	-2.3618	-0.693	-0.3109
24	H	-2.9716	-2.1538	0.5123
25	H	-1.984	-0.9687	1.4084
26	C	1.7979	-3.5267	-4.3292
27	H	2.725	-4.096	-4.4191
28	H	0.9531	-4.1787	-4.5582
29	C	-0.23	2.9518	-1.882
30	H	-1.0636	2.7082	-2.5463
31	H	-0.5418	3.7581	-1.2154
32	C	1.1365	-2.8086	4.9591
33	H	2.0458	-2.4448	5.443
34	H	0.2805	-2.2786	5.3817
35	C	1.8075	-2.2836	-5.1957
36	C	2.9373	-1.4512	-5.2073
37	C	0.6749	-1.9265	-5.9351
38	C	2.9244	-0.2726	-5.9548
39	H	3.8168	-1.7243	-4.6277
40	C	0.6669	-0.7481	-6.6878
41	H	-0.201	-2.5711	-5.924
42	C	1.7913	0.0804	-6.6966
43	H	3.7965	0.3743	-5.9564
44	H	-0.2144	-0.481	-7.2649
45	H	1.7879	0.9971	-7.2802
46	C	0.9864	-4.3091	5.098
47	C	-0.1796	-4.8589	5.6406
48	C	2.0186	-5.158	4.6736
49	C	-0.3133	-6.2451	5.7655
50	H	-0.9824	-4.2026	5.9682
51	C	1.8826	-6.5411	4.7929
52	H	2.9243	-4.7318	4.2485
53	C	0.7163	-7.0873	5.3406
54	H	-1.2212	-6.664	6.1912
55	H	2.6871	-7.1926	4.4624
56	H	0.613	-8.165	5.4351
57	C	1.0009	3.3325	-2.6732
58	C	1.1857	2.8503	-3.975
59	C	1.968	4.1736	-2.1081
60	C	2.3179	3.2147	-4.7073
61	H	0.4417	2.193	-4.4192

62	C	3.0975	4.5407	-2.8411
63	H	1.8343	4.5363	-1.0921
64	C	3.2722	4.0648	-4.1436
65	H	2.4532	2.8326	-5.7148
66	H	3.8441	5.1906	-2.3945
67	H	4.1561	4.3433	-4.7099
68	N	5.1905	-1.5056	-0.9998
69	S	6.0414	-2.6115	-1.8753
70	O	5.9215	-4.0235	-1.4434
71	O	5.7768	-2.3286	-3.31
72	C	7.6996	-2.0604	-1.4879
73	C	8.5636	-2.8893	-0.7736
74	C	8.097	-0.7815	-1.8979
75	C	9.8486	-2.4327	-0.4759
76	H	8.2301	-3.8718	-0.4572
77	C	9.3784	-0.3413	-1.588
78	H	7.4091	-0.1431	-2.4413
79	C	10.2741	-1.1574	-0.8734
80	H	10.5284	-3.075	0.0781
81	H	9.69	0.6533	-1.8981
82	C	11.66	-0.6585	-0.5481
83	H	12.2371	-0.4757	-1.4638
84	H	11.6179	0.2919	-0.0017
85	H	12.2149	-1.3777	0.0627
86	Cl	5.5747	-1.9215	1.9578
87	C	3.4604	1.683	0.5943
88	H	2.6498	1.2839	1.1951
89	C	3.6577	1.2536	-0.661
90	H	2.9837	0.4924	-1.0514
91	H	4.0916	2.431	1.0681
92	C	4.7027	1.6813	-1.6058
93	C	5.6652	2.6614	-1.3013
94	C	4.7482	1.0796	-2.875
95	C	6.635	3.0251	-2.2345
96	H	5.6593	3.1426	-0.3277
97	C	5.7183	1.4403	-3.81
98	H	4.0219	0.3123	-3.1229
99	C	6.6669	2.4174	-3.4952
100	H	7.3701	3.7837	-1.9773
101	H	5.7432	0.9463	-4.7776
102	H	7.4266	2.6988	-4.2197

TS56-+-s-2

	Atom	X	Y	Z
1	C	-2.353	-0.9868	0.3559
2	C	-2.4807	-1.9593	-0.8034
3	C	-1.1581	-1.4236	1.1915
4	C	-2.1772	0.3865	-0.2914
5	C	-3.1207	1.3691	-0.5078
6	H	-4.1377	1.4914	-0.1703
7	C	-3.5166	-2.7612	-1.2212
8	H	-4.4993	-2.96	-0.8256
9	C	-1.1011	-2.3136	2.2454
10	H	-1.859	-2.8367	2.8064
11	N	0.2228	-2.4529	2.499
12	N	-2.5046	2.2611	-1.3209
13	N	-3.047	-3.3361	-2.3602
14	N	-1.0638	0.7302	-0.9996
15	N	-1.2614	1.8695	-1.6226
16	N	-1.8021	-2.9205	-2.6409
17	N	-1.4586	-2.0883	-1.6912
18	N	0.1217	-1.1025	0.8553
19	N	0.958	-1.7165	1.6563
20	Cu	0.3892	-0.7397	-1.3576
21	O	-3.5724	-1.0587	1.0673
22	C	-3.6617	-0.2884	2.2742
23	H	-3.2766	0.7293	2.1452
24	H	-4.7258	-0.2423	2.5162
25	H	-3.1252	-0.7663	3.1008
26	C	-3.7745	-4.1654	-3.3252
27	H	-3.0895	-4.9429	-3.6691
28	H	-4.5874	-4.6451	-2.7726
29	C	-3.0917	3.4212	-2.0117
30	H	-3.8492	3.84	-1.347
31	H	-2.2866	4.1496	-2.1294
32	C	0.8794	-3.2774	3.5199
33	H	1.3555	-2.6071	4.2406
34	H	0.0767	-3.81	4.0379
35	C	-4.3	-3.3482	-4.4917
36	C	-4.9895	-2.1467	-4.278
37	C	-4.1086	-3.8014	-5.8009
38	C	-5.4751	-1.4087	-5.3578
39	H	-5.1392	-1.7777	-3.2666
40	C	-4.6041	-3.0687	-6.8838
41	H	-3.5639	-4.7258	-5.9749
42	C	-5.2834	-1.8682	-6.6653
43	H	-5.9956	-0.4726	-5.1788
44	H	-4.4483	-3.4318	-7.8961
45	H	-5.6607	-1.2926	-7.5063
46	C	1.892	-4.241	2.9313
47	C	3.0508	-4.5395	3.6579
48	C	1.6811	-4.8582	1.6923
49	C	3.9801	-5.457	3.1606
50	H	3.2284	-4.0508	4.6131

51	C	2.6155	-5.7656	1.1911
52	H	0.8118	-4.6048	1.0943
53	C	3.764	-6.0728	1.9251
54	H	4.8775	-5.6785	3.7325
55	H	2.451	-6.2171	0.2169
56	H	4.4942	-6.7733	1.529
57	C	-3.6782	3.0098	-3.3471
58	C	-5.0621	2.8848	-3.511
59	C	-2.8281	2.7087	-4.4206
60	C	-5.5925	2.4673	-4.7354
61	H	-5.7264	3.1179	-2.6823
62	C	-3.356	2.2842	-5.6408
63	H	-1.7528	2.8035	-4.2956
64	C	-4.7406	2.1639	-5.8004
65	H	-6.6688	2.3789	-4.8556
66	H	-2.6888	2.0481	-6.4647
67	H	-5.1517	1.8358	-6.751
68	N	2.1467	-1.3298	-2.1557
69	S	2.3466	-3.0174	-2.1306
70	O	1.2636	-3.5574	-1.2949
71	O	2.561	-3.5778	-3.4801
72	C	3.8794	-3.144	-1.2206
73	C	3.9064	-2.7083	0.1105
74	C	5.0027	-3.7047	-1.8263
75	C	5.0837	-2.8489	0.8351
76	H	3.0189	-2.2893	0.5726
77	C	6.1735	-3.8426	-1.0769
78	H	4.957	-4.0376	-2.8578
79	C	6.2313	-3.4224	0.2584
80	H	5.1109	-2.5283	1.8731
81	H	7.051	-4.2893	-1.5374
82	C	7.4843	-3.5898	1.0803
83	H	7.2924	-4.2273	1.9529
84	H	8.292	-4.0444	0.4984
85	H	7.8392	-2.6241	1.4613
86	C	1.3901	-0.3106	-3.6679
87	C	2.7787	-0.6388	-3.2936
88	H	3.3167	-1.2927	-3.9799
89	H	0.9956	0.592	-3.2056
90	C	0.5485	-0.9291	-4.662
91	C	-0.7917	-0.4837	-4.7515
92	C	1.0134	-1.8978	-5.5779
93	C	-1.6389	-0.9826	-5.7324
94	H	-1.1557	0.2456	-4.0334
95	C	0.1596	-2.3924	-6.5571
96	H	2.0295	-2.2649	-5.5101
97	C	-1.1628	-1.9387	-6.6368
98	H	-2.668	-0.6432	-5.7865
99	H	0.5219	-3.1359	-7.2611
100	H	-1.8276	-2.3355	-7.3976
101	H	3.3959	0.204	-2.9791

TS45-+-t-2

	Atom	X	Y	Z
1	C	0.6693	-0.8237	0.0091
2	C	0.9036	-1.7897	-1.1389
3	C	1.7965	-0.9776	1.0211
4	C	0.6653	0.572	-0.613
5	C	-0.3499	1.4911	-0.7554
6	H	-1.3687	1.5208	-0.4047
7	C	0.1602	-2.8396	-1.6206
8	H	-0.7687	-3.2849	-1.3031
9	C	1.8909	-1.7784	2.1405
10	H	1.2009	-2.4486	2.6281
11	N	3.1436	-1.5573	2.6072
12	N	0.1914	2.4857	-1.5038
13	N	0.8549	-3.2707	-2.7068
14	N	1.7411	1.0614	-1.2932
15	N	1.4563	2.2223	-1.8324
16	N	1.9567	-2.5412	-2.9085
17	N	1.9847	-1.6487	-1.9531
18	N	2.9919	-0.3459	0.8613
19	N	3.8069	-0.6935	1.8256
20	Cu	3.3897	-0.1292	-1.5612
21	O	-0.5994	-1.1499	0.5318
22	C	-1.0107	-0.477	1.7335
23	H	-2.1025	-0.4431	1.7008
24	H	-0.6962	-1.0304	2.6241
25	H	-0.6193	0.5436	1.7946
26	C	0.4395	-4.251	-3.7096
27	H	1.3498	-4.5442	-4.2399
28	H	0.0592	-5.1282	-3.1806
29	C	-0.4768	3.6766	-2.0482
30	H	-1.3373	3.8631	-1.4007
31	H	0.2177	4.5119	-1.945
32	C	3.7567	-2.0792	3.8366
33	H	4.0575	-1.2191	4.4399
34	H	2.9551	-2.5991	4.3692
35	C	-0.6012	-3.6931	-4.6662
36	C	-0.761	-2.3173	-4.8699
37	C	-1.3963	-4.5891	-5.3916
38	C	-1.6962	-1.8446	-5.7943
39	H	-0.1621	-1.6063	-4.3096
40	C	-2.3293	-4.1164	-6.3167

41	H	-1.2834	-5.659	-5.2324
42	C	-2.4815	-2.7418	-6.5217
43	H	-1.8037	-0.7737	-5.9448
44	H	-2.9405	-4.8223	-6.8725
45	H	-3.2088	-2.374	-7.2403
46	C	4.9325	-3.0014	3.5885
47	C	6.0844	-2.8743	4.3732
48	C	4.8665	-4.0138	2.6236
49	C	7.1555	-3.756	4.2045
50	H	6.1438	-2.0836	5.1176
51	C	5.9416	-4.8852	2.4459
52	H	3.9931	-4.0983	1.9863
53	C	7.086	-4.763	3.2392
54	H	8.0463	-3.6467	4.8175
55	H	5.8881	-5.6542	1.6801
56	H	7.9233	-5.4406	3.0959
57	C	-0.8951	3.4844	-3.4932
58	C	-1.673	2.3825	-3.8753
59	C	-0.5225	4.427	-4.4572
60	C	-2.0748	2.2298	-5.2029
61	H	-1.9659	1.6407	-3.1365
62	C	-0.9301	4.2783	-5.7863
63	H	0.088	5.2789	-4.1685
64	C	-1.706	3.1793	-6.1622
65	H	-2.6826	1.3747	-5.486
66	H	-0.634	5.0168	-6.5261
67	H	-2.0205	3.061	-7.1955
68	N	5.0929	-0.9341	-1.5506
69	S	5.2707	-2.5513	-1.2246
70	O	4.2314	-2.9408	-0.2433
71	O	5.3767	-3.3669	-2.454
72	C	6.8562	-2.5685	-0.4
73	C	7.0013	-1.8519	0.7947
74	C	7.9172	-3.2885	-0.9465
75	C	8.233	-1.8647	1.4382
76	H	6.1612	-1.308	1.2146
77	C	9.1429	-3.2991	-0.2769
78	H	7.7826	-3.8326	-1.8754
79	C	9.3193	-2.5918	0.9194
80	H	8.3538	-1.3176	2.3693
81	H	9.972	-3.8665	-0.6918
82	C	10.6302	-2.6231	1.6639
83	H	10.5209	-3.1667	2.6117
84	H	11.4157	-3.1155	1.0821
85	H	10.9696	-1.6104	1.9131
86	C	5.6577	0.6799	-3.9354
87	C	6.1223	-0.5719	-3.6646
88	H	5.6061	-1.4686	-3.9839
89	H	6.287	1.5308	-3.6778
90	C	4.3404	1.0122	-4.4617
91	C	3.3559	0.0338	-4.7356
92	C	4.0133	2.3676	-4.6873
93	C	2.1048	0.4037	-5.2198
94	H	3.5613	-1.0152	-4.5491
95	C	2.7603	2.7318	-5.1746
96	H	4.7572	3.1321	-4.4762
97	C	1.7999	1.7516	-5.4432
98	H	1.3644	-0.3626	-5.4273
99	H	2.5251	3.7797	-5.3377
100	H	0.8199	2.0367	-5.8119
101	H	7.125	-0.7096	-3.2738

TS45-+-s

	Atom	X	Y	Z
1	C	-1.9221	1.5986	0.0827
2	C	-1.8983	0.3171	-0.726
3	C	-0.6961	1.634	0.98
4	C	-1.8527	2.7599	-0.8984
5	C	-2.2012	2.7889	-2.2372
6	H	-2.5913	2.0416	-2.9103
7	C	-2.9289	-0.4231	-1.2634
8	H	-4.0016	-0.359	-1.1756
9	C	-0.5471	1.5221	2.3443
10	H	-1.2572	1.4249	3.1501
11	N	0.7954	1.5597	2.5484
12	N	-1.9083	4.0444	-2.6352
13	N	-2.3045	-1.3536	-2.0297
14	N	-1.3623	3.9888	-0.5616
15	N	-1.3949	4.7657	-1.6149
16	N	-0.9736	-1.2046	-1.9856
17	N	-0.7262	-0.1918	-1.1946
18	N	0.5567	1.7201	0.4572
19	N	1.4623	1.6832	1.4053
20	Cu	1.0369	1.4992	-1.4482
21	O	-3.1133	1.5545	0.8565
22	C	-3.5454	2.7955	1.4259
23	H	-3.9332	3.4767	0.6596
24	H	-4.3495	2.5359	2.1184
25	H	-2.7414	3.299	1.9755
26	C	-2.8924	-2.2705	-3.0151
27	H	-2.184	-3.0959	-3.1207
28	H	-3.822	-2.6591	-2.5954
29	C	-1.9702	4.6062	-3.9944
30	H	-2.8966	4.2467	-4.4477

31	H	-2.0376	5.6886	-3.8686
32	C	1.5361	1.5065	3.8284
33	H	1.7026	2.5356	4.1569
34	H	0.8671	1.0164	4.5395
35	C	-3.1283	-1.5588	-4.3353
36	C	-2.057	-0.957	-5.0134
37	C	-4.4156	-1.4868	-4.8782
38	C	-2.2793	-0.2948	-6.2218
39	H	-1.0517	-0.9996	-4.6027
40	C	-4.6353	-0.8257	-6.0909
41	H	-5.2482	-1.9505	-4.3542
42	C	-3.568	-0.2262	-6.7633
43	H	-1.4419	0.1632	-6.7409
44	H	-5.639	-0.7775	-6.5047
45	H	-3.7366	0.2896	-7.7049
46	C	2.8374	0.757	3.6646
47	C	4.0529	1.4482	3.6388
48	C	2.829	-0.6336	3.4868
49	C	5.252	0.7572	3.4414
50	H	4.061	2.5272	3.7706
51	C	4.024	-1.3226	3.2805
52	H	1.8843	-1.1722	3.5013
53	C	5.2386	-0.6277	3.2589
54	H	6.1928	1.3007	3.4301
55	H	4.0107	-2.3996	3.1398
56	H	6.1697	-1.1657	3.1052
57	C	-0.7621	4.211	-4.8168
58	C	-0.8093	3.0855	-5.6482
59	C	0.4272	4.945	-4.7204
60	C	0.3203	2.692	-6.3681
61	H	-1.728	2.5095	-5.7246
62	C	1.5547	4.5571	-5.4486
63	H	0.4662	5.8214	-4.0777
64	C	1.5045	3.4264	-6.2696
65	H	0.2802	1.8069	-6.9963
66	H	2.4709	5.1371	-5.3747
67	H	2.3859	3.1127	-6.8202
68	N	1.3377	1.3092	-3.2732
69	S	2.3568	0.2761	-4.0146
70	O	1.372	-0.5896	-4.7218
71	O	3.3888	0.9208	-4.8523
72	C	3.2418	-0.6987	-2.7936
73	C	2.5429	-1.3243	-1.7543
74	C	4.6301	-0.82	-2.8929
75	C	3.2505	-2.0411	-0.7908
76	H	1.4628	-1.2623	-1.7021
77	C	5.3196	-1.5492	-1.9276
78	H	5.1575	-0.3315	-3.7027
79	C	4.6473	-2.1607	-0.8576
80	H	2.7086	-2.5207	0.0202
81	H	6.4007	-1.6366	-1.999
82	C	5.4225	-2.9067	0.1982
83	H	6.1759	-3.5623	-0.2534
84	H	5.9539	-2.2043	0.8527
85	H	4.7664	-3.5165	0.8269
86	C	1.9262	3.4026	-1.6538
87	H	1.0585	3.9067	-2.0726
88	C	2.9485	3.0018	-2.526
89	H	2.8201	3.2148	-3.581
90	H	2.1338	3.5823	-0.6021
91	C	4.2437	2.5254	-2.0858
92	C	5.3168	2.542	-3.0002
93	C	4.4672	2.0693	-0.7671
94	C	6.5916	2.1558	-2.5935
95	H	5.1367	2.8573	-4.0223
96	C	5.737	1.6704	-0.372
97	H	3.6414	2.0072	-0.0635
98	C	6.8031	1.7214	-1.2816
99	H	7.4162	2.1821	-3.2998
100	H	5.8966	1.3095	0.6397
101	H	7.7955	1.4094	-0.9681

TS45-+-oss-2

	Atom	X	Y	Z
1	C	1.7289	-0.6435	0.0166
2	C	1.8702	-1.6582	-1.1047
3	C	2.9289	-0.7651	0.9486
4	C	1.7133	0.7202	-0.6772
5	C	0.7035	1.6423	-0.836
6	H	-0.302	1.7036	-0.4518
7	C	1.0174	-2.6109	-1.6135
8	H	0.0428	-2.9597	-1.3129
9	C	3.0854	-1.4271	2.1472
10	H	2.4105	-1.9893	2.7723
11	N	4.3885	-1.2331	2.4732
12	N	1.2294	2.5866	-1.6581
13	N	1.6796	-3.1018	-2.6944
14	N	2.769	1.157	-1.4217
15	N	2.4786	2.2899	-2.0144
16	N	2.8592	-2.4983	-2.865
17	N	2.971	-1.626	-1.8998
18	N	4.1417	-0.2412	0.6146
19	N	5.0253	-0.5212	1.5389
20	Cu	4.4325	-0.0266	-1.593

21	O	0.4998	-0.9142	0.6458
22	C	0.1692	-0.1427	1.8132
23	H	-0.9209	-0.0677	1.8284
24	H	0.505	-0.6472	2.7249
25	H	0.5991	0.8636	1.7837
26	C	1.1902	-4.0191	-3.7389
27	H	1.9549	-4.7833	-3.8896
28	H	0.2932	-4.4938	-3.3359
29	C	0.5505	3.7458	-2.2574
30	H	-0.2694	3.9998	-1.5809
31	H	1.2647	4.571	-2.2603
32	C	5.0936	-1.6627	3.6886
33	H	5.5218	-0.769	4.1488
34	H	4.3205	-2.0457	4.3608
35	C	0.9136	-3.248	-5.0127
36	C	-0.1771	-2.3704	-5.08
37	C	1.7888	-3.3421	-6.1002
38	C	-0.3886	-1.5961	-6.2217
39	H	-0.8569	-2.289	-4.2347
40	C	1.5738	-2.5719	-7.2464
41	H	2.6434	-4.0114	-6.0456
42	C	0.4884	-1.6949	-7.3071
43	H	-1.2342	-0.9155	-6.2649
44	H	2.2606	-2.6495	-8.0847
45	H	0.3253	-1.0897	-8.1947
46	C	6.1643	-2.7047	3.4323
47	C	7.3551	-2.6549	4.1672
48	C	5.9681	-3.7394	2.5104
49	C	8.3347	-3.6357	3.9922
50	H	7.5164	-1.8466	4.8769
51	C	6.9527	-4.7114	2.327
52	H	5.0684	-3.7653	1.9059
53	C	8.1355	-4.666	3.0697
54	H	9.2567	-3.586	4.5653
55	H	6.7994	-5.497	1.5923
56	H	8.9029	-5.4207	2.9204
57	C	0.0512	3.4435	-3.657
58	C	-0.7825	2.3428	-3.8969
59	C	0.4144	4.2732	-4.7226
60	C	-1.244	2.0765	-5.1862
61	H	-1.066	1.6877	-3.0766
62	C	-0.0529	4.0112	-6.0138
63	H	1.0653	5.125	-4.5425
64	C	-0.8803	2.9107	-6.2489
65	H	-1.8885	1.2198	-5.362
66	H	0.2374	4.6617	-6.8341
67	H	-1.239	2.702	-7.253
68	N	6.1478	-0.8297	-1.6727
69	S	6.3072	-2.4532	-1.4828
70	O	5.3615	-2.8717	-0.4186
71	O	6.2602	-3.2117	-2.7547
72	C	7.9661	-2.6109	-0.8245
73	C	8.2645	-1.9873	0.3928
74	C	8.9278	-3.3529	-1.5081
75	C	9.5438	-2.1169	0.9217
76	H	7.5016	-1.4207	0.9165
77	C	10.2067	-3.4757	-0.9594
78	H	8.6762	-3.8263	-2.4513
79	C	10.5325	-2.8658	0.2593
80	H	9.7797	-1.6401	1.8693
81	H	10.9604	-4.0557	-1.4861
82	C	11.9012	-3.0297	0.8727
83	H	11.8711	-3.7567	1.6956
84	H	12.6314	-3.389	0.1404
85	H	12.2678	-2.0853	1.2912
86	C	6.5878	0.8234	-4.0876
87	C	7.1673	-0.3699	-3.767
88	H	6.74	-1.3168	-4.0702
89	H	7.144	1.7397	-3.8933
90	C	5.2306	1.0034	-4.5778
91	C	4.2992	-0.0615	-4.6495
92	C	4.7979	2.2935	-4.9613
93	C	2.9943	0.1624	-5.0818
94	H	4.5891	-1.0598	-4.3393
95	C	3.4992	2.5069	-5.4126
96	H	5.4984	3.1235	-4.9053
97	C	2.5908	1.443	-5.4724
98	H	2.2904	-0.6632	-5.1083
99	H	3.1829	3.5038	-5.7053
100	H	1.5731	1.6153	-5.8081
101	H	8.1853	-0.3971	-3.3926

TS34-+

	Atom	X	Y	Z
1	C	-1.4054	-1.6636	-0.0049
2	C	-0.4895	-2.0692	-1.1397
3	C	-0.6554	-1.7183	1.3167
4	C	-1.821	-0.2315	-0.2977
5	C	-2.091	0.3405	-1.5256
6	H	-2.0911	-0.0473	-2.5324
7	C	-0.678	-2.902	-2.2157
8	H	-1.4826	-3.5618	-2.4973
9	C	-0.9877	-2.304	2.5171
10	H	-1.8358	-2.8881	2.8357

11	N	0.0354	-1.9828	3.35
12	N	-2.3246	1.6394	-1.2454
13	N	0.4304	-2.7186	-2.9827
14	N	-1.9083	0.7389	0.6564
15	N	-2.2059	1.8754	0.0803
16	N	1.263	-1.8305	-2.4437
17	N	0.7016	-1.4396	-1.3245
18	N	0.5448	-1.1022	1.5048
19	N	0.9658	-1.2595	2.7354
20	Cu	1.6129	-0.2525	0.0225
21	O	-2.4718	-2.6012	-0.0027
22	C	-3.7102	-2.1469	0.5599
23	H	-4.216	-1.4413	-0.1094
24	H	-4.3251	-3.0421	0.6756
25	H	-3.5716	-1.6729	1.5383
26	C	0.6755	-3.218	-4.3455
27	H	1.7419	-3.066	-4.5289
28	H	0.4676	-4.2899	-4.3478
29	C	-2.5199	2.7406	-2.1916
30	H	-3.4702	2.5891	-2.7109
31	H	-2.6077	3.6415	-1.5776
32	C	0.2335	-2.3908	4.7563
33	H	0.4273	-1.4821	5.3293
34	H	-0.7183	-2.8166	5.0805
35	C	-0.1905	-2.4744	-5.3434
36	C	-0.099	-1.0782	-5.4489
37	C	-1.1026	-3.1681	-6.145
38	C	-0.9138	-0.3873	-6.3456
39	H	0.604	-0.5312	-4.8252
40	C	-1.9127	-2.4756	-7.0504
41	H	-1.1803	-4.2494	-6.0619
42	C	-1.8219	-1.0859	-7.1491
43	H	-0.8385	0.6938	-6.4143
44	H	-2.6171	-3.0226	-7.6711
45	H	-2.4558	-0.5473	-7.8483
46	C	1.3695	-3.3808	4.8768
47	C	1.203	-4.6997	4.4307
48	C	2.6112	-2.9726	5.3752
49	C	2.2715	-5.5959	4.4742
50	H	0.2387	-5.0193	4.0423
51	C	3.6799	-3.8721	5.4245
52	H	2.7451	-1.948	5.7109
53	C	3.5127	-5.1819	4.9701
54	H	2.1376	-6.6156	4.1238
55	H	4.6422	-3.5452	5.8077
56	H	4.3449	-5.8797	5.0016
57	C	-1.3718	2.8406	-3.18
58	C	-1.6297	3.1901	-4.5102
59	C	-0.051	2.5974	-2.778
60	C	-0.5804	3.3036	-5.427
61	H	-2.6527	3.3716	-4.8314
62	C	0.995	2.6936	-3.6972
63	H	0.1689	2.3229	-1.7506
64	C	0.7336	3.0491	-5.0245
65	H	-0.7926	3.578	-6.457
66	H	2.0102	2.4876	-3.3703
67	H	1.5474	3.125	-5.7406
68	N	2.8749	0.6324	1.1142
69	I	5.0988	-0.9848	2.7835
70	S	3.4153	1.6115	-0.036
71	C	4.3175	-2.3033	1.3282
72	O	2.3303	1.1959	-1.0813
73	O	3.527	3.0407	0.2784
74	C	4.9475	1.1053	-0.7908
75	C	4.8227	-2.2595	0.0245
76	C	3.2782	-3.1725	1.6719
77	C	4.9273	0.3799	-1.9847
78	C	6.1495	1.405	-0.1402
79	C	4.2525	-3.08	-0.953
80	H	5.641	-1.5972	-0.2286
81	C	2.7234	-3.9906	0.6837
82	H	2.8978	-3.2034	2.6834
83	C	6.1376	-0.0428	-2.534
84	H	3.9875	0.1464	-2.4718
85	C	7.3426	0.9525	-0.694
86	H	6.1548	1.9782	0.7811
87	C	3.2005	-3.941	-0.6291
88	H	4.6327	-3.035	-1.9697
89	H	1.9111	-4.6621	0.9485
90	C	7.3575	0.2236	-1.8965
91	H	6.1305	-0.5973	-3.4684
92	H	8.28	1.1737	-0.1906
93	H	2.7559	-4.57	-1.3951
94	C	8.6619	-0.27	-2.4695
95	H	9.4093	0.5314	-2.4985
96	H	9.0778	-1.0737	-1.8482
97	H	8.5347	-0.6607	-3.4837

i9-+

	Atom	X	Y	Z
1	C	0.1618	-0.388	0.059
2	C	1.2917	-0.735	-0.8881
3	C	0.6996	-0.377	1.4796
4	C	-0.3393	0.9811	-0.3533

5	C	-0.2062	1.5733	-1.5925	52	H	4.0915	-0.6003	5.3644
6	H	0.238	1.2429	-2.5172	53	C	5.244	-3.6626	4.4155
7	C	1.3691	-1.6888	-1.8755	54	H	3.9978	-5.3092	3.7876
8	H	0.6895	-2.4591	-2.2012	55	H	6.2145	-1.8577	5.097
9	C	0.4635	-1.2461	2.5229	56	H	6.173	-4.2114	4.2878
10	H	-0.1957	-2.092	2.6363	57	C	0.6754	3.9922	-3.0816
11	N	1.281	-0.8137	3.5153	58	C	0.7591	4.1737	-4.4659
12	N	-0.7171	2.8106	-1.4342	59	C	1.8576	3.8936	-2.3325
13	N	2.5821	-1.4795	-2.4481	60	C	2.0062	4.2657	-5.0932
14	N	-0.9397	1.8748	0.4847	61	H	-0.1517	4.2421	-5.0561
15	N	-1.1648	2.9863	-0.17	62	C	3.1025	3.9878	-2.9548
16	N	3.224	-0.4592	-1.8727	63	H	1.8074	3.747	-1.2585
17	N	2.4395	0.0019	-0.928	64	C	3.1793	4.1709	-4.3401
18	N	1.6492	0.5096	1.8867	65	H	2.0579	4.4096	-6.1692
19	N	2.0037	0.2428	3.1246	66	H	4.0038	3.9308	-2.3518
20	Cu	3.0502	1.4399	0.5111	67	H	4.1482	4.2409	-4.8275
21	O	-0.7926	-1.4358	-0.0892	68	N	5.0032	1.7359	1.0913
22	C	-2.1119	-1.1518	0.3866	69	I	5.8421	0.1816	2.1216
23	H	-2.6245	-0.4282	-0.2582	70	S	6.1047	2.6655	0.2468
24	H	-2.6474	-2.1038	0.3568	71	C	5.2902	-1.4407	0.8825
25	H	-2.1067	-0.77	1.4143	72	O	5.2654	3.7689	-0.2691
26	C	3.1636	-2.1127	-3.6327	73	O	7.2877	2.9936	1.0668
27	H	4.247	-2.0711	-3.4922	74	C	6.6767	1.7066	-1.1512
28	H	2.8606	-3.1614	-3.627	75	C	6.0883	-1.777	-0.2136
29	C	-0.6791	3.9126	-2.4022	76	C	4.1123	-2.1245	1.1944
30	H	-1.4776	3.7693	-3.1355	77	C	5.7677	1.3798	-2.1591
31	H	-0.8938	4.8185	-1.8295	78	C	7.9974	1.2549	-1.1968
32	C	1.5636	-1.4456	4.8164	79	C	5.684	-2.8434	-1.0189
33	H	1.6297	-0.6422	5.5531	80	H	6.9915	-1.2234	-0.4427
34	H	0.7023	-2.0726	5.0543	81	C	3.7301	-3.1899	0.3771
35	C	2.7427	-1.4257	-4.9195	82	H	3.5154	-1.8477	2.0543
36	C	2.7051	-0.0271	-5.0177	83	C	6.1914	0.5908	-3.2249
37	C	2.4213	-2.1992	-6.0408	84	H	4.7411	1.7203	-2.1054
38	C	2.3521	0.585	-6.2215	85	C	8.4042	0.4627	-2.2711
39	H	2.9466	0.5871	-4.1551	86	H	8.689	1.5202	-0.4045
40	C	2.0757	-1.5855	-7.2479	87	C	4.5128	-3.5479	-0.7237
41	H	2.4422	-3.2841	-5.9707	88	H	6.289	-3.1193	-1.8776
42	C	2.0384	-0.1922	-7.3405	89	H	2.8193	-3.7349	0.6067
43	H	2.3222	1.6693	-6.2796	90	C	7.5113	0.1195	-3.2988
44	H	1.8282	-2.1971	-8.1115	91	H	5.482	0.3298	-4.0057
45	H	1.7645	0.2856	-8.2773	92	H	9.4306	0.1067	-2.3118
46	C	2.8468	-2.2448	4.7397	93	H	4.2091	-4.3778	-1.3552
47	C	2.8278	-3.5732	4.2963	94	C	7.9625	-0.7225	-4.4661
48	C	4.0734	-1.6325	5.0256	95	H	8.3696	-0.0908	-5.2671
49	C	4.0217	-4.2796	4.1336	96	H	8.7509	-1.4241	-4.1734
50	H	1.8775	-4.053	4.0743	97	H	7.1302	-1.2925	-4.8931
51	C	5.2681	-2.3395	4.8663	98	C	1.4236	7.6085	-0.8668

99	C	0.2779	7.043	-0.3018
100	C	0.3871	5.9351	0.541
101	C	1.6417	5.3735	0.8413
102	C	2.789	5.9563	0.2665
103	C	2.678	7.0582	-0.578
104	H	1.3418	8.4652	-1.5305
105	H	-0.7019	7.4603	-0.5197
106	H	-0.5083	5.4854	0.9606
107	H	3.7653	5.5186	0.4449
108	H	3.5734	7.484	-1.0235
109	C	1.7008	4.1921	1.718
110	H	0.7344	3.7608	1.9766
111	C	2.8108	3.6162	2.2176
112	H	2.7418	2.7718	2.8957
113	H	3.8076	3.9888	2.0116

i7-+

	Atom	X	Y	Z
1	C	-1.5574	0.1319	0.0571
2	C	-1.1043	-0.1381	-1.3607
3	C	-0.5247	-0.1966	1.1195
4	C	-1.8842	1.6184	0.1105
5	C	-2.2116	2.4726	-0.9279
6	H	-2.2864	2.342	-1.996
7	C	-1.915	-0.4781	-2.4254
8	H	-2.9404	-0.8045	-2.4819
9	C	-0.7291	-0.7235	2.379
10	H	-1.6018	-1.1091	2.8813
11	N	0.4878	-0.6713	2.9716
12	N	-2.4391	3.6603	-0.328
13	N	-1.1387	-0.2847	-3.5175
14	N	-1.9349	2.3288	1.2715
15	N	-2.2579	3.5697	1.0064
16	N	0.0763	0.1497	-3.1711
17	N	0.1063	0.2372	-1.8611
18	N	0.7938	0.1312	1.0216
19	N	1.4054	-0.1538	2.1472
20	Cu	1.9893	0.5866	-0.7765
21	O	-2.7063	-0.7054	0.2441
22	C	-3.7669	-0.209	1.0639
23	H	-4.3121	0.6059	0.5718
24	H	-4.4418	-1.0569	1.206
25	H	-3.4175	0.1418	2.041
26	C	-1.5394	-0.2038	-4.9332
27	H	-0.7554	-0.6826	-5.5231
28	H	-2.4634	-0.776	-5.0339
29	C	-2.7214	4.9559	-0.9624
30	H	-3.3532	4.7506	-1.8299
31	H	-3.2947	5.5369	-0.2379
32	C	0.8365	-0.9954	4.3634
33	H	0.8558	-0.0579	4.9268
34	H	0.0119	-1.604	4.7448
35	C	-1.7221	1.2529	-5.3103
36	C	-0.6648	1.9715	-5.8815
37	C	-2.9158	1.9171	-4.9968
38	C	-0.7979	3.3389	-6.1363
39	H	0.2651	1.4618	-6.1195
40	C	-3.0489	3.285	-5.2485
41	H	-3.7403	1.3629	-4.5541
42	C	-1.9884	3.9985	-5.8173
43	H	0.0276	3.887	-6.5822
44	H	-3.9792	3.7922	-5.007
45	H	-2.0902	5.0626	-6.0117
46	C	2.1578	-1.7206	4.4771
47	C	2.401	-2.8823	3.7316
48	C	3.1411	-1.2474	5.3522
49	C	3.6149	-3.557	3.8582
50	H	1.6468	-3.2462	3.0383
51	C	4.3541	-1.9298	5.4875
52	H	2.9571	-0.3456	5.9311
53	C	4.5936	-3.0837	4.739
54	H	3.7998	-4.4488	3.2659
55	H	5.112	-1.5523	6.1687
56	H	5.54	-3.6088	4.8342
57	C	-1.4469	5.6671	-1.3632
58	C	-0.7659	5.2935	-2.5297
59	C	-0.9083	6.6673	-0.5438
60	C	0.4377	5.9143	-2.8718
61	H	-1.1763	4.5199	-3.1737
62	C	0.292	7.2954	-0.8914
63	H	-1.4308	6.9549	0.3649
64	C	0.9676	6.9183	-2.0543
65	H	0.955	5.624	-3.7818
66	H	0.699	8.0727	-0.2506
67	H	1.9031	7.4012	-2.3222
68	N	3.264	-0.8263	-1.8606
69	S	3.8796	-2.1551	-0.8849
70	O	2.7183	-2.6196	-0.1133
71	O	4.5862	-3.1003	-1.7694
72	C	5.0661	-1.3975	0.1899
73	C	4.6749	-1.0362	1.4784
74	C	6.3807	-1.2346	-0.2547
75	C	5.6324	-0.5043	2.3364
76	H	3.6507	-1.1702	1.8033

77	C	7.3137	-0.6746	0.6133
78	H	6.6713	-1.5503	-1.2507
79	C	6.9542	-0.2997	1.9169
80	H	5.3441	-0.2371	3.3467
81	H	8.3379	-0.5354	0.2774
82	C	7.9499	0.3429	2.8485
83	H	7.7815	1.4271	2.8926
84	H	7.8457	-0.0416	3.8696
85	H	8.9807	0.1783	2.5188
86	C	4.1264	-0.4258	-3.0404
87	C	2.9298	-1.2731	-3.245
88	H	3.0913	-2.323	-3.4721
89	H	2.0093	-0.8419	-3.6178
90	H	5.0771	-0.9516	-3.0799
91	C	4.1938	1.0117	-3.4267
92	C	5.4492	1.6079	-3.5978
93	C	3.0402	1.776	-3.6594
94	C	5.5548	2.942	-3.9943
95	H	6.3474	1.025	-3.4111
96	C	3.1455	3.1127	-4.0449
97	H	2.0571	1.3351	-3.5435
98	C	4.402	3.6999	-4.2162
99	H	6.5364	3.3909	-4.1201
100	H	2.2415	3.6908	-4.2169
101	H	4.4824	4.7407	-4.5187
102	C	1.9803	5.1324	4.4402
103	C	1.9888	5.0861	3.0444
104	C	1.2874	4.0574	2.4104
105	C	0.5831	3.0824	3.1194
106	C	0.5898	3.1468	4.5144
107	C	1.2865	4.1647	5.1724
108	H	2.5264	5.92	4.951
109	H	2.5391	5.8236	2.4707
110	H	0.0259	2.3118	2.6044
111	H	0.042	2.4006	5.0834
112	H	1.2876	4.2057	6.258
113	I	1.3522	3.966	0.289
114	N	2.6724	2.4485	-0.072
115	S	4.2755	2.8891	0.0833
116	O	5.0054	1.6941	-0.3799
117	O	4.5997	4.1955	-0.5299
118	C	4.5588	3.0717	1.8478
119	C	3.9429	2.194	2.7446
120	C	5.4348	4.0584	2.3005
121	C	4.2122	2.315	4.1039
122	H	3.2534	1.4375	2.3893
123	C	5.6958	4.1641	3.6688
124	H	5.8963	4.7401	1.5937
125	C	5.0916	3.2966	4.5891
126	H	3.7294	1.635	4.8009
127	H	6.3742	4.9354	4.0243
128	C	5.3473	3.4188	6.0706
129	H	5.6605	2.4589	6.499
130	H	6.1242	4.1586	6.2878
131	H	4.4341	3.7231	6.5989

i6-+

	Atom	X	Y	Z
1	C	3.2291	-3.0418	0.0033
2	C	3.4267	-3.9701	-1.1786
3	C	4.2749	-3.3031	1.0741
4	C	3.3414	-1.6283	-0.541
5	C	3.0757	-1.1936	-1.8266
6	H	2.7801	-1.7092	-2.7265
7	C	2.5013	-4.6548	-1.9336
8	H	1.4426	-4.8213	-1.8202
9	C	4.1194	-3.7968	2.3501
10	H	3.25	-4.0825	2.9201
11	N	5.3801	-3.8754	2.8427
12	N	3.3006	0.1358	-1.7872
13	N	3.2187	-5.1427	-2.9777
14	N	3.7103	-0.5488	0.2054
15	N	3.6853	0.5202	-0.5503
16	N	4.5056	-4.7844	-2.8992
17	N	4.6304	-4.0713	-1.8051
18	N	5.6121	-3.1285	0.8663
19	N	6.284	-3.4794	1.944
20	Cu	6.4122	-3.1292	-0.971
21	O	1.936	-3.354	0.5174
22	C	1.3057	-2.3414	1.3086
23	H	0.9521	-1.5096	0.6876
24	H	0.4495	-2.8289	1.7814
25	H	1.9715	-1.9463	2.0847
26	C	2.7195	-5.7812	-4.2063
27	H	3.4416	-6.5506	-4.4848
28	H	1.7729	-6.2607	-3.9485
29	C	3.236	1.0991	-2.8989
30	H	2.2274	1.0469	-3.3177
31	H	3.378	2.0817	-2.4458
32	C	5.831	-4.4648	4.1151
33	H	6.7034	-3.8867	4.428
34	H	5.029	-4.311	4.8389
35	C	2.5536	-4.746	-5.3
36	C	3.5455	-4.5815	-6.2739

37	C	1.4364	-3.8997	-5.3048
38	C	3.4172	-3.5882	-7.2486
39	H	4.4167	-5.2317	-6.2682
40	C	1.3116	-2.9009	-6.2728
41	H	0.6631	-4.0252	-4.5503
42	C	2.303	-2.7443	-7.2476
43	H	4.1874	-3.4727	-8.0064
44	H	0.4397	-2.2523	-6.2721
45	H	2.2053	-1.9703	-8.0039
46	C	6.1607	-5.9333	3.945
47	C	5.2853	-6.9186	4.4139
48	C	7.3336	-6.315	3.2779
49	C	5.5775	-8.2719	4.2201
50	H	4.3762	-6.6277	4.9349
51	C	7.6229	-7.6651	3.0786
52	H	8.017	-5.5533	2.9129
53	C	6.744	-8.6466	3.5494
54	H	4.8937	-9.0303	4.5915
55	H	8.5345	-7.9508	2.5606
56	H	6.9709	-9.6983	3.3968
57	C	4.2844	0.8117	-3.9531
58	C	4.0486	-0.1511	-4.9458
59	C	5.5157	1.477	-3.9235
60	C	5.0336	-0.4469	-5.8901
61	H	3.0916	-0.6664	-4.987
62	C	6.4996	1.1864	-4.8716
63	H	5.7074	2.2159	-3.1507
64	C	6.2615	0.2221	-5.8544
65	H	4.8394	-1.1976	-6.6501
66	H	7.4525	1.7067	-4.835
67	H	7.0277	-0.0062	-6.5904
68	N	7.7735	-2.9308	-2.4789
69	S	8.7964	-4.3468	-2.6709
70	O	7.9121	-5.5032	-2.4908
71	O	9.5828	-4.2074	-3.9056
72	C	9.8387	-4.115	-1.2488
73	C	9.2917	-4.2945	0.0284
74	C	11.1446	-3.6589	-1.4293
75	C	10.0734	-3.9882	1.1365
76	H	8.2736	-4.6494	0.1559
77	C	11.9155	-3.3747	-0.3017
78	H	11.5466	-3.5252	-2.4278
79	C	11.3931	-3.5255	0.9907
80	H	9.6526	-4.1009	2.1319
81	H	12.9352	-3.0225	-0.4303
82	C	12.2216	-3.2103	2.2099
83	H	11.6627	-2.5804	2.9117
84	H	12.4892	-4.1302	2.746
85	H	13.149	-2.6933	1.9459
86	C	8.4133	-1.6271	-2.9004
87	C	7.3271	-2.2306	-3.7128
88	H	7.6035	-2.682	-4.6616
89	H	6.3117	-1.8558	-3.6458
90	H	9.4059	-1.7614	-3.3235
91	C	8.2951	-0.4687	-1.9761
92	C	9.4599	0.2338	-1.637
93	C	7.0695	-0.0665	-1.426
94	C	9.403	1.3185	-0.7597
95	H	10.4132	-0.0747	-2.059
96	C	7.0131	1.0118	-0.5425
97	H	6.1551	-0.5929	-1.6878
98	C	8.179	1.7089	-0.2108
99	H	10.3131	1.8553	-0.5064
100	H	6.0545	1.3106	-0.1312
101	H	8.1319	2.5523	0.4728

i5-+-t

	Atom	X	Y	Z
1	C	-0.6485	-0.8126	-0.0215
2	C	-0.432	-1.7638	-1.1856
3	C	0.464	-1.0223	0.9953
4	C	-0.6121	0.5912	-0.6208
5	C	-1.6089	1.5282	-0.7766
6	H	-2.6312	1.5742	-0.4376
7	C	-1.1862	-2.7993	-1.6815
8	H	-2.1227	-3.2352	-1.3735
9	C	0.5543	-1.9299	2.0315
10	H	-0.1304	-2.6619	2.43
11	N	1.7889	-1.7258	2.5488
12	N	-1.0423	2.5107	-1.5222
13	N	-0.4918	-3.2289	-2.7686
14	N	0.4787	1.0613	-1.2894
15	N	0.2207	2.2249	-1.8371
16	N	0.6203	-2.513	-2.9581
17	N	0.6538	-1.6297	-1.9939
18	N	1.6431	-0.3465	0.9272
19	N	2.4436	-0.7688	1.8745
20	Cu	2.0792	-0.1441	-1.6194
21	O	-1.9311	-1.1111	0.4872
22	C	-2.3099	-0.4633	1.7124
23	H	-3.4025	-0.4585	1.7209
24	H	-1.9441	-1.0147	2.5847
25	H	-1.9434	0.5673	1.767
26	C	-0.9253	-4.1849	-3.791

27	H	-0.0242	-4.4546	-4.3487
28	H	-1.2918	-5.0782	-3.2804
29	C	-1.684	3.7096	-2.0806
30	H	-2.5541	3.9077	-1.4494
31	H	-0.9808	4.5361	-1.9652
32	C	2.3923	-2.3567	3.7321
33	H	2.6047	-1.5639	4.4537
34	H	1.6116	-2.9953	4.1545
35	C	-1.9892	-3.5886	-4.6962
36	C	-1.9618	-2.2321	-5.0467
37	C	-3.0004	-4.4089	-5.2087
38	C	-2.9298	-1.7056	-5.9036
39	H	-1.1888	-1.5819	-4.6478
40	C	-3.9663	-3.8827	-6.0708
41	H	-3.0336	-5.4601	-4.9324
42	C	-3.9341	-2.5301	-6.4196
43	H	-2.8964	-0.6518	-6.1662
44	H	-4.748	-4.5287	-6.4613
45	H	-4.6887	-2.1206	-7.0857
46	C	3.6431	-3.1523	3.4222
47	C	4.802	-2.9454	4.1785
48	C	3.6461	-4.1225	2.4119
49	C	5.9487	-3.708	3.9385
50	H	4.8086	-2.1856	4.9563
51	C	4.7945	-4.8748	2.1639
52	H	2.7632	-4.2663	1.7982
53	C	5.9471	-4.6732	2.9295
54	H	6.8445	-3.5364	4.5295
55	H	4.7921	-5.6126	1.3662
56	H	6.8426	-5.2556	2.73
57	C	-2.079	3.5222	-3.533
58	C	-2.849	2.4211	-3.932
59	C	-1.6977	4.473	-4.4856
60	C	-3.2358	2.2779	-5.2651
61	H	-3.1496	1.6733	-3.2025
62	C	-2.0917	4.3345	-5.8197
63	H	-1.0924	5.3241	-4.1837
64	C	-2.8608	3.2367	-6.2124
65	H	-3.8375	1.4231	-5.5612
66	H	-1.7904	5.0801	-6.5503
67	H	-3.1659	3.1267	-7.2495
68	N	3.7641	-0.9881	-1.8867
69	S	3.9782	-2.5591	-1.3276
70	O	2.913	-2.8085	-0.3412
71	O	4.1429	-3.5122	-2.4446
72	C	5.5455	-2.4243	-0.4794
73	C	5.6105	-1.6709	0.6988
74	C	6.6785	-3.0344	-1.0156
75	C	6.836	-1.5408	1.342
76	H	4.7149	-1.2183	1.1116
77	C	7.8987	-2.8955	-0.3507
78	H	6.6037	-3.6107	-1.9316
79	C	7.9964	-2.1506	0.8319
80	H	6.8953	-0.9694	2.2645
81	H	8.7853	-3.3742	-0.7583
82	C	9.3047	-2.0231	1.5706
83	H	9.2698	-2.5814	2.5156
84	H	10.142	-2.4113	0.9823
85	H	9.5181	-0.9779	1.8245
86	C	4.1694	0.6984	-3.5874
87	C	4.4946	-0.6934	-3.1467
88	H	4.2468	-1.436	-3.9152
89	H	4.8134	1.4999	-3.2338
90	C	3.0096	1.0505	-4.3251
91	C	2.0711	0.0824	-4.8031
92	C	2.7187	2.4253	-4.5821
93	C	0.9194	0.4764	-5.472
94	H	2.2415	-0.9722	-4.6141
95	C	1.5634	2.8021	-5.2492
96	H	3.4171	3.1805	-4.2292
97	C	0.6507	1.8335	-5.6966
98	H	0.2221	-0.2785	-5.8242
99	H	1.3568	3.8553	-5.4188
100	H	-0.2592	2.1359	-6.2048
101	H	5.5723	-0.7815	-2.9497

i5-+-t-freq

	Atom	X	Y	Z
1	C	-0.4818	-0.4	0.0052
2	C	-0.2653	-1.3511	-1.1589
3	C	0.6307	-0.6096	1.0221
4	C	-0.4454	1.0039	-0.5941
5	C	-1.4422	1.9409	-0.7498
6	H	-2.4645	1.9869	-0.4109
7	C	-1.0195	-2.3866	-1.6547
8	H	-1.956	-2.8225	-1.3468
9	C	0.721	-1.5172	2.0582
10	H	0.0363	-2.2492	2.4567
11	N	1.9556	-1.3131	2.5755
12	N	-0.8756	2.9233	-1.4954
13	N	-0.3251	-2.8162	-2.7419
14	N	0.6454	1.4739	-1.2626
15	N	0.3875	2.6376	-1.8104
16	N	0.787	-2.1003	-2.9313

17	N	0.8205	-1.2171	-1.9672
18	N	1.8099	0.0661	0.954
19	N	2.6104	-0.3561	1.9013
20	Cu	2.2459	0.2686	-1.5926
21	O	-1.7644	-0.6985	0.514
22	C	-2.1432	-0.0507	1.7392
23	H	-3.2357	-0.0458	1.7477
24	H	-1.7773	-0.6021	2.6114
25	H	-1.7767	0.98	1.7938
26	C	-0.7585	-3.7722	-3.7642
27	H	0.1425	-4.0419	-4.322
28	H	-1.125	-4.6655	-3.2536
29	C	-1.5173	4.1223	-2.0538
30	H	-2.3874	4.3204	-1.4226
31	H	-0.8141	4.9488	-1.9384
32	C	2.559	-1.9441	3.7589
33	H	2.7714	-1.1513	4.4804
34	H	1.7783	-2.5827	4.1812
35	C	-1.8225	-3.1759	-4.6694
36	C	-1.7951	-1.8194	-5.0199
37	C	-2.8337	-3.9963	-5.1819
38	C	-2.7631	-1.2929	-5.8768
39	H	-1.022	-1.1692	-4.621
40	C	-3.7996	-3.47	-6.044
41	H	-2.8669	-5.0474	-4.9057
42	C	-3.7674	-2.1174	-6.3928
43	H	-2.7297	-0.2391	-6.1395
44	H	-4.5812	-4.116	-6.4345
45	H	-4.5219	-1.7079	-7.0589
46	C	3.8098	-2.7397	3.4489
47	C	4.9687	-2.5328	4.2052
48	C	3.8128	-3.7099	2.4387
49	C	6.1155	-3.2953	3.9653
50	H	4.9754	-1.7729	4.9831
51	C	4.9612	-4.4621	2.1907
52	H	2.9299	-3.8536	1.825
53	C	6.1138	-4.2606	2.9563
54	H	7.0112	-3.1237	4.5563
55	H	4.9588	-5.2	1.3929
56	H	7.0093	-4.8429	2.7568
57	C	-1.9122	3.9348	-3.5062
58	C	-2.6823	2.8338	-3.9053
59	C	-1.5309	4.8857	-4.4588
60	C	-3.0691	2.6906	-5.2383
61	H	-2.9829	2.086	-3.1757
62	C	-1.925	4.7472	-5.793
63	H	-0.9257	5.7367	-4.1569
64	C	-2.6941	3.6493	-6.1857
65	H	-3.6708	1.8358	-5.5345
66	H	-1.6237	5.4928	-6.5235
67	H	-2.9992	3.5394	-7.2227
68	N	3.9308	-0.5754	-1.8599
69	S	4.1449	-2.1464	-1.3009
70	O	3.0797	-2.3958	-0.3144
71	O	4.3096	-3.0995	-2.4178
72	C	5.7122	-2.0116	-0.4526
73	C	5.7772	-1.2582	0.7256
74	C	6.8453	-2.6217	-0.9889
75	C	7.0027	-1.1281	1.3688
76	H	4.8816	-0.8056	1.1384
77	C	8.0654	-2.4828	-0.3239
78	H	6.7705	-3.1981	-1.9049
79	C	8.1631	-1.738	0.8586
80	H	7.062	-0.5568	2.2912
81	H	8.952	-2.9615	-0.7315
82	C	9.4714	-1.6104	1.5974
83	H	9.4365	-2.1688	2.5424
84	H	10.3088	-1.9987	1.0091
85	H	9.6848	-0.5652	1.8513
86	C	4.3361	1.1111	-3.5607
87	C	4.6613	-0.2807	-3.1199
88	H	4.4136	-1.0233	-3.8884
89	H	4.9801	1.9125	-3.207
90	C	3.1764	1.4632	-4.2983
91	C	2.2378	0.4951	-4.7763
92	C	2.8854	2.838	-4.5554
93	C	1.0861	0.8891	-5.4452
94	H	2.4082	-0.5595	-4.5873
95	C	1.7301	3.2148	-5.2224
96	H	3.5838	3.5932	-4.2024
97	C	0.8174	2.2462	-5.6698
98	H	0.3889	0.1342	-5.7974
99	H	1.5235	4.268	-5.3921
100	H	-0.0925	2.5486	-6.178
101	H	5.739	-0.3688	-2.923

i5-+-s

	Atom	X	Y	Z
1	C	-1.2046	-1.4738	0.4444
2	C	-1.1055	-2.391	-0.7599
3	C	-0.0341	-1.763	1.3728
4	C	-1.1448	-0.0513	-0.1082
5	C	-2.1491	0.8728	-0.3023
6	H	-3.1981	0.8826	-0.0553

7	C	-2.0064	-3.2729	-1.3104
8	H	-2.998	-3.5864	-1.0261
9	C	0.0305	-2.5442	2.5071
10	H	-0.7134	-3.0967	3.0589
11	N	1.3354	-2.4966	2.8747
12	N	-1.5509	1.8986	-0.9566
13	N	-1.3731	-3.7345	-2.4199
14	N	-0.0164	0.466	-0.6761
15	N	-0.2624	1.6508	-1.1844
16	N	-0.1605	-3.1681	-2.5595
17	N	0.002	-2.3554	-1.5485
18	N	1.2236	-1.3184	1.1141
19	N	2.0531	-1.7518	2.0291
20	Cu	1.7211	-0.5921	-0.8589
21	O	-2.4374	-1.7647	1.0629
22	C	-2.7973	-0.9962	2.2231
23	H	-3.4961	-0.1974	1.9547
24	H	-3.2929	-1.683	2.9145
25	H	-1.9269	-0.5569	2.7202
26	C	-1.9013	-4.5938	-3.4774
27	H	-1.0299	-4.9335	-4.0447
28	H	-2.3616	-5.4674	-3.0091
29	C	-2.1838	3.0929	-1.5441
30	H	-3.0577	3.314	-0.928
31	H	-1.467	3.9099	-1.4463
32	C	1.9853	-3.1422	4.0216
33	H	2.3235	-2.3553	4.7015
34	H	1.2	-3.7085	4.5308
35	C	-2.8911	-3.869	-4.3732
36	C	-2.7671	-2.498	-4.632
37	C	-3.9248	-4.5905	-4.9819
38	C	-3.6627	-1.8596	-5.4925
39	H	-1.9694	-1.9271	-4.1676
40	C	-4.8172	-3.9528	-5.8475
41	H	-4.0312	-5.6537	-4.7789
42	C	-4.6889	-2.585	-6.1042
43	H	-3.5575	-0.7946	-5.6812
44	H	-5.6156	-4.5235	-6.3143
45	H	-5.3858	-2.0876	-6.7737
46	C	3.1406	-4.0404	3.6202
47	C	4.1958	-4.2235	4.5227
48	C	3.1663	-4.7076	2.3903
49	C	5.2591	-5.0713	4.2041
50	H	4.1883	-3.6972	5.4745
51	C	4.2362	-5.5445	2.0677
52	H	2.383	-4.5428	1.659
53	C	5.2822	-5.7336	2.9736
54	H	6.0741	-5.2019	4.9111
55	H	4.2565	-6.0334	1.098
56	H	6.1183	-6.3783	2.7167
57	C	-2.5591	2.8462	-2.9903
58	C	-3.7098	2.1105	-3.3024
59	C	-1.7385	3.313	-4.0241
60	C	-4.038	1.8461	-4.633
61	H	-4.3499	1.7457	-2.5026
62	C	-2.0676	3.0513	-5.357
63	H	-0.8448	3.884	-3.7853
64	C	-3.2168	2.3166	-5.6631
65	H	-4.934	1.2775	-4.8659
66	H	-1.4271	3.4198	-6.1534
67	H	-3.4743	2.115	-6.6993
68	N	3.588	-1.0197	-1.1236
69	S	4.0134	-2.6314	-1.1953
70	O	2.9544	-3.4184	-0.5392
71	O	4.4473	-3.0339	-2.5545
72	C	5.4661	-2.6749	-0.1438
73	C	5.3445	-2.2784	1.1937
74	C	6.6742	-3.1527	-0.6483
75	C	6.4535	-2.372	2.0262
76	H	4.3928	-1.9242	1.5736
77	C	7.776	-3.2442	0.2066
78	H	6.7485	-3.4552	-1.6873
79	C	7.6831	-2.8624	1.5507
80	H	6.3625	-2.0788	3.0688
81	H	8.7187	-3.6238	-0.1788
82	C	8.859	-2.9885	2.4866
83	H	8.6326	-3.6901	3.2999
84	H	9.7529	-3.3488	1.9677
85	H	9.1004	-2.0255	2.9536
86	C	2.5672	0.2706	-2.6642
87	C	3.9565	-0.1673	-2.2712
88	H	4.4823	-0.6989	-3.0679
89	H	2.2579	1.2611	-2.3282
90	C	1.805	-0.3	-3.7481
91	C	2.196	-1.4813	-4.4244
92	C	0.6323	0.3739	-4.1752
93	C	1.4523	-1.9473	-5.5014
94	H	3.058	-2.042	-4.081
95	C	-0.099	-0.0958	-5.257
96	H	0.3149	1.2695	-3.6513
97	C	0.3072	-1.2599	-5.9221
98	H	1.7561	-2.8574	-6.0102
99	H	-0.987	0.4387	-5.5794
100	H	-0.276	-1.6363	-6.7575

101	H	4.5703	0.6753	-1.939

i5-+-s-freq

	Atom	X	Y	Z
1	C	0.1909	0.0364	0
2	C	0.2901	-0.8808	-1.2043
3	C	1.3614	-0.2529	0.9284
4	C	0.2508	1.4588	-0.5526
5	C	-0.7536	2.383	-0.7467
6	H	-1.8026	2.3928	-0.4997
7	C	-0.6109	-1.7627	-1.7548
8	H	-1.6024	-2.0762	-1.4705
9	C	1.426	-1.0341	2.0627
10	H	0.6821	-1.5865	2.6145
11	N	2.7309	-0.9865	2.4304
12	N	-0.1554	3.4088	-1.401
13	N	0.0224	-2.2243	-2.8642
14	N	1.3791	1.9762	-1.1204
15	N	1.1331	3.161	-1.6288
16	N	1.235	-1.6579	-3.0039
17	N	1.3976	-0.8452	-1.9928
18	N	2.6192	0.1918	0.6698
19	N	3.4486	-0.2417	1.5847
20	Cu	3.1166	0.9181	-1.3033
21	O	-1.0419	-0.2545	0.6185
22	C	-1.4018	0.514	1.7787
23	H	-2.1006	1.3128	1.5103
24	H	-1.8974	-0.1728	2.4701
25	H	-0.5313	0.9533	2.2758
26	C	-0.5058	-3.0837	-3.9218
27	H	0.3656	-3.4233	-4.4891
28	H	-0.966	-3.9572	-3.4534
29	C	-0.7882	4.6031	-1.9885
30	H	-1.6621	4.8242	-1.3724
31	H	-0.0715	5.4201	-1.8906
32	C	3.3808	-1.6321	3.5773
33	H	3.719	-0.8451	4.2572
34	H	2.5956	-2.1983	4.0864
35	C	-1.4956	-2.3588	-4.8175
36	C	-1.3716	-0.9878	-5.0763
37	C	-2.5293	-3.0803	-5.4262
38	C	-2.2672	-0.3494	-5.9369
39	H	-0.5739	-0.4169	-4.612
40	C	-3.4216	-2.4427	-6.2919
41	H	-2.6356	-4.1435	-5.2233
42	C	-3.2934	-1.0748	-6.5486
43	H	-2.162	0.7155	-6.1256
44	H	-4.2201	-3.0133	-6.7586
45	H	-3.9902	-0.5774	-7.2181
46	C	4.5361	-2.5302	3.1758
47	C	5.5913	-2.7133	4.0783
48	C	4.5618	-3.1974	1.9459
49	C	6.6547	-3.5611	3.7598
50	H	5.5838	-2.187	5.0301
51	C	5.6317	-4.0343	1.6234
52	H	3.7785	-3.0326	1.2146
53	C	6.6777	-4.2234	2.5293
54	H	7.4697	-3.6917	4.4667
55	H	5.652	-4.5233	0.6536
56	H	7.5139	-4.8681	2.2723
57	C	-1.1636	4.3563	-3.4346
58	C	-2.3143	3.6206	-3.7468
59	C	-0.3429	4.8232	-4.4684
60	C	-2.6425	3.3562	-5.0773
61	H	-2.9544	3.2558	-2.947
62	C	-0.6721	4.5615	-5.8013
63	H	0.5507	5.3942	-4.2296
64	C	-1.8212	3.8268	-6.1075
65	H	-3.5384	2.7877	-5.3102
66	H	-0.0316	4.93	-6.5977
67	H	-2.0787	3.6251	-7.1437
68	N	4.9835	0.4905	-1.568
69	S	5.4089	-1.1212	-1.6396
70	O	4.35	-1.9082	-0.9836
71	O	5.8428	-1.5238	-2.9989
72	C	6.8616	-1.1648	-0.5882
73	C	6.7401	-0.7682	0.7493
74	C	8.0697	-1.6426	-1.0926
75	C	7.849	-0.8619	1.5819
76	H	5.7884	-0.4141	1.1292
77	C	9.1715	-1.734	-0.2378
78	H	8.144	-1.945	-2.1316
79	C	9.0786	-1.3522	1.1064
80	H	7.758	-0.5686	2.6244
81	H	10.1143	-2.1136	-0.6232
82	C	10.2546	-1.4784	2.0422
83	H	10.0281	-2.1799	2.8556
84	H	11.1485	-1.8386	1.5233
85	H	10.4959	-0.5153	2.5093
86	C	3.9627	1.7808	-3.1086
87	C	5.352	1.3429	-2.7155
88	H	5.8778	0.8113	-3.5123
89	H	3.6534	2.7713	-2.7726
90	C	3.2005	1.2102	-4.1925

91	C	3.5915	0.0288	-4.8687
92	C	2.0278	1.8841	-4.6196
93	C	2.8479	-0.4371	-5.9458
94	H	4.4536	-0.5318	-4.5254
95	C	1.2965	1.4143	-5.7013
96	H	1.7105	2.7796	-4.0957
97	C	1.7027	0.2503	-6.3665
98	H	3.1516	-1.3472	-6.4546
99	H	0.4086	1.9489	-6.0237
100	H	1.1195	-0.1261	-7.2019
101	H	5.9658	2.1855	-2.3834

i4-+t

	Atom	X	Y	Z
1	C	1.9322	0.6284	-0.0483
2	C	2.3055	-0.1616	-1.2874
3	C	3.0468	0.5347	0.9818
4	C	1.8014	2.0772	-0.4881
5	C	1.6363	2.5689	-1.7666
6	H	1.5155	2.0924	-2.7268
7	C	1.5475	-0.9895	-2.0802
8	H	0.5416	-1.3662	-1.9888
9	C	3.0489	0.1236	2.2945
10	H	2.2762	-0.2548	2.9446
11	N	4.3317	0.2998	2.7049
12	N	1.7319	3.9084	-1.6287
13	N	2.3512	-1.2754	-3.1379
14	N	1.9797	3.1356	0.3575
15	N	1.9485	4.2427	-0.3361
16	N	3.533	-0.6727	-3.0388
17	N	3.5038	0.0077	-1.9154
18	N	4.3184	0.9177	0.6842
19	N	5.103	0.7698	1.7272
20	Cu	5.0342	1.1398	-1.2349
21	O	0.7437	0.0332	0.4476
22	C	-0.0636	0.853	1.303
23	H	-0.5661	1.6466	0.7381
24	H	-0.8119	0.1813	1.7295
25	H	0.5207	1.3069	2.1111
26	C	1.9875	-1.9561	-4.3927
27	H	2.9229	-2.3293	-4.8153
28	H	1.353	-2.8047	-4.1318
29	C	1.9851	4.8741	-2.7092
30	H	1.1627	4.8151	-3.4259
31	H	1.9816	5.8613	-2.2429
32	C	4.926	-0.0372	4.0116
33	H	5.7537	0.6609	4.1519
34	H	4.1637	0.1655	4.7657
35	C	1.283	-0.9933	-5.3275
36	C	1.9638	0.1298	-5.8205
37	C	-0.0533	-1.2011	-5.6831
38	C	1.3108	1.0327	-6.6602
39	H	3.0016	0.2955	-5.5428
40	C	-0.7052	-0.2991	-6.5298
41	H	-0.5839	-2.0691	-5.2994
42	C	-0.0256	0.819	-7.017
43	H	1.8453	1.8972	-7.0447
44	H	-1.7426	-0.4702	-6.8039
45	H	-0.532	1.5209	-7.674
46	C	5.3902	-1.4766	4.0604
47	C	4.6741	-2.4279	4.795
48	C	6.538	-1.867	3.3565
49	C	5.1022	-3.7582	4.8309
50	H	3.7854	-2.1263	5.3441
51	C	6.964	-3.1952	3.3909
52	H	7.0932	-1.13	2.7824
53	C	6.2465	-4.1432	4.1287
54	H	4.5432	-4.4903	5.4074
55	H	7.8592	-3.4891	2.8497
56	H	6.5814	-5.1765	4.1581
57	C	3.3173	4.534	-3.3482
58	C	3.3713	3.9091	-4.5993
59	C	4.5017	4.7306	-2.6242
60	C	4.597	3.484	-5.1236
61	H	2.453	3.7412	-5.1571
62	C	5.7253	4.3086	-3.1482
63	H	4.4615	5.2018	-1.6451
64	C	5.7737	3.6784	-4.3979
65	H	4.6296	2.9979	-6.095
66	H	6.6398	4.4682	-2.5862
67	H	6.7257	3.3348	-4.7896
68	N	6.6216	2.2846	-0.8638
69	S	7.5392	1.5066	-1.9978
70	O	6.4826	0.731	-2.7798
71	O	8.4566	2.3467	-2.7751
72	C	8.4264	0.3677	-0.9717
73	C	7.7929	-0.8104	-0.5465
74	C	9.7089	0.7122	-0.5278
75	C	8.4692	-1.6535	0.3259
76	H	6.8019	-1.0685	-0.9063
77	C	10.3637	-0.1464	0.3495
78	H	10.1834	1.6248	-0.873
79	C	9.7554	-1.3319	0.7961
80	H	7.9928	-2.5724	0.6528

81	H	11.3622	0.1055	0.695
82	C	10.4484	-2.2317	1.7844
83	H	10.2659	-3.2878	1.5578
84	H	11.5287	-2.0584	1.7977
85	H	10.0698	-2.0461	2.7986

i4-+t-freq

	Atom	X	Y	Z
1	C	-1.0778	-1.0145	0.0141
2	C	-0.7045	-1.8045	-1.2249
3	C	0.0368	-1.1082	1.0442
4	C	-1.2086	0.4343	-0.4256
5	C	-1.3737	0.926	-1.7042
6	H	-1.4945	0.4496	-2.6644
7	C	-1.4625	-2.6324	-2.0178
8	H	-2.4684	-3.0091	-1.9263
9	C	0.0389	-1.5193	2.357
10	H	-0.7338	-1.8976	3.0071
11	N	1.3217	-1.3431	2.7673
12	N	-1.2781	2.2655	-1.5662
13	N	-0.6588	-2.9183	-3.0754
14	N	-1.0302	1.4927	0.4199
15	N	-1.0615	2.5998	-0.2736
16	N	0.523	-2.3156	-2.9764
17	N	0.4939	-1.6352	-1.8529
18	N	1.3084	-0.7252	0.7466
19	N	2.093	-0.8731	1.7897
20	Cu	2.0242	-0.5031	-1.1725
21	O	-2.2663	-1.6097	0.5101
22	C	-3.0736	-0.7899	1.3655
23	H	-3.5761	0.0037	0.8005
24	H	-3.8219	-1.4616	1.7919
25	H	-2.4893	-0.336	2.1736
26	C	-1.0224	-3.599	-4.3302
27	H	-0.0871	-3.9722	-4.7529
28	H	-1.657	-4.4476	-4.0694
29	C	-1.0249	3.2313	-2.6468
30	H	-1.8473	3.1722	-3.3634
31	H	-1.0284	4.2184	-2.1805
32	C	1.916	-1.6801	4.0741
33	H	2.7437	-0.982	4.2143
34	H	1.1537	-1.4774	4.8281
35	C	-1.727	-2.6362	-5.2651
36	C	-1.0462	-1.5131	-5.7581
37	C	-3.0633	-2.8439	-5.6207
38	C	-1.6992	-0.6102	-6.5978
39	H	-0.0084	-1.3474	-5.4803
40	C	-3.7152	-1.942	-6.4674
41	H	-3.5939	-3.7119	-5.237
42	C	-3.0356	-0.8239	-6.9546
43	H	-1.1647	0.2543	-6.9823
44	H	-4.7526	-2.1131	-6.7414
45	H	-3.5419	-0.122	-7.6116
46	C	2.3802	-3.1195	4.1228
47	C	1.6641	-4.0708	4.8574
48	C	3.5281	-3.5099	3.419
49	C	2.0922	-5.4011	4.8933
50	H	0.7754	-3.7692	5.4066
51	C	3.9541	-4.8381	3.4533
52	H	4.0832	-2.7729	2.8448
53	C	3.2365	-5.7861	4.1912
54	H	1.5333	-6.1332	5.4698
55	H	4.8492	-5.132	2.9122
56	H	3.5715	-6.8194	4.2206
57	C	0.3073	2.8911	-3.2858
58	C	0.3613	2.2662	-4.5368
59	C	1.4918	3.0878	-2.5617
60	C	1.587	1.8411	-5.0611
61	H	-0.557	2.0983	-5.0946
62	C	2.7153	2.6657	-3.0858
63	H	1.4515	3.559	-1.5826
64	C	2.7637	2.0356	-4.3355
65	H	1.6196	1.3551	-6.0325
66	H	3.6299	2.8253	-2.5238
67	H	3.7157	1.6919	-4.7271
68	N	3.6116	0.6417	-0.8013
69	S	4.5292	-0.1363	-1.9354
70	O	3.4726	-0.9119	-2.7173
71	O	5.4466	0.7038	-2.7127
72	C	5.4164	-1.2752	-0.9093
73	C	4.7829	-2.4533	-0.4841
74	C	6.6989	-0.9307	-0.4654
75	C	5.4592	-3.2963	0.3883
76	H	3.7919	-2.7114	-0.8439
77	C	7.3537	-1.7893	0.4119
78	H	7.1734	-0.0181	-0.8105
79	C	6.7454	-2.9748	0.8586
80	H	4.9828	-4.2153	0.7152
81	H	8.3522	-1.5374	0.7574
82	C	7.4384	-3.8746	1.8469
83	H	7.2559	-4.9307	1.6203
84	H	8.5188	-3.7013	1.8602
85	H	7.0599	-3.689	2.861

i4-+s

	Atom	X	Y	Z
1	C	1.8163	0.5602	0.0953
2	C	2.396	-0.2654	-1.0342
3	C	2.6916	0.4102	1.3274
4	C	1.855	2.0064	-0.3683
5	C	1.7999	2.5007	-1.6571
6	H	1.6971	2.029	-2.622
7	C	1.789	-1.022	-2.0064
8	H	0.765	-1.3194	-2.1649
9	C	2.4021	0.0242	2.6147
10	H	1.4852	-0.2714	3.0988
11	N	3.5917	0.0862	3.2682
12	N	1.9705	3.8316	-1.5108
13	N	2.796	-1.3471	-2.8604
14	N	2.0487	3.0528	0.4857
15	N	2.1251	4.1577	-0.2083
16	N	3.9605	-0.8355	-2.4716
17	N	3.7146	-0.1816	-1.3613
18	N	4.0281	0.6658	1.2937
19	N	4.5795	0.465	2.4668
20	Cu	5.0757	0.9054	-0.3495
21	O	0.5211	0.0398	0.3435
22	C	-0.4107	0.9345	0.9666
23	H	-0.7318	1.721	0.2741
24	H	-1.2699	0.3169	1.2374
25	H	0.0023	1.3997	1.8692
26	C	2.6811	-1.9894	-4.1806
27	H	3.7056	-2.2085	-4.4904
28	H	2.1429	-2.9301	-4.0493
29	C	2.1342	4.8409	-2.563
30	H	1.1771	4.9706	-3.0751
31	H	2.3718	5.7704	-2.0383
32	C	3.8939	-0.3354	4.6511
33	H	4.7083	0.3069	4.9919
34	H	3.0026	-0.1219	5.2435
35	C	1.9709	-1.066	-5.1497
36	C	2.4763	0.2203	-5.3939
37	C	0.7942	-1.4769	-5.7833
38	C	1.8067	1.0825	-6.2621
39	H	3.3891	0.5485	-4.9012
40	C	0.1293	-0.615	-6.6613
41	H	0.3962	-2.4701	-5.5902
42	C	0.632	0.6655	-6.8985
43	H	2.2034	2.0775	-6.4403
44	H	-0.7831	-0.9436	-7.1515
45	H	0.1126	1.3382	-7.5757
46	C	4.2666	-1.8013	4.6991
47	C	3.3461	-2.7519	5.154
48	C	5.5292	-2.2183	4.2548
49	C	3.6838	-4.1079	5.1689
50	H	2.3669	-2.4298	5.5005
51	C	5.8646	-3.5727	4.2654
52	H	6.2458	-1.4803	3.9026
53	C	4.9419	-4.5198	4.7228
54	H	2.9655	-4.8394	5.5289
55	H	6.8465	-3.8889	3.9236
56	H	5.2052	-5.574	4.7345
57	C	3.2256	4.4453	-3.5381
58	C	2.9978	4.5239	-4.9158
59	C	4.4701	3.9957	-3.0717
60	C	4.0019	4.1625	-5.82
61	H	2.0332	4.8658	-5.2832
62	C	5.4681	3.624	-3.9729
63	H	4.6597	3.9365	-2.0022
64	C	5.2353	3.7059	-5.3502
65	H	3.8143	4.2297	-6.8883
66	H	6.4234	3.2756	-3.5952
67	H	6.0131	3.4167	-6.0517
68	N	6.4184	1.9851	0.4309
69	S	7.3489	1.9695	-0.8598
70	O	6.3199	1.1809	-1.7586
71	O	7.809	3.2382	-1.435
72	C	8.7506	0.8967	-0.6824
73	C	8.638	-0.229	0.1457
74	C	9.9248	1.1692	-1.3899
75	C	9.7205	-1.094	0.249
76	H	7.7257	-0.4085	0.7069
77	C	10.9975	0.2886	-1.2693
78	H	9.9967	2.0518	-2.0161
79	C	10.914	-0.8514	-0.4554
80	H	9.6476	-1.9671	0.8922
81	H	11.9156	0.4921	-1.8132
82	C	12.0716	-1.8082	-0.3382
83	H	11.8377	-2.7596	-0.8338
84	H	12.9775	-1.4027	-0.7987
85	H	12.289	-2.0383	0.7114

i4-+s-freq

	Atom	X	Y	Z
1	C	-0.9691	0.8333	0.0127
2	C	-0.3894	0.0078	-1.1168
3	C	-0.0938	0.6833	1.2448
4	C	-0.9304	2.2796	-0.4508

5	C	-0.9854	2.7739	-1.7396
6	H	-1.0883	2.3022	-2.7046
7	C	-0.9964	-0.7489	-2.089
8	H	-2.0204	-1.0463	-2.2475
9	C	-0.3833	0.2973	2.5321
10	H	-1.3002	0.0018	3.0162
11	N	0.8063	0.3594	3.1856
12	N	-0.8149	4.1048	-1.5934
13	N	0.0106	-1.074	-2.9429
14	N	-0.7367	3.326	0.4032
15	N	-0.6603	4.4308	-0.2909
16	N	1.1751	-0.5623	-2.5542
17	N	0.9292	0.0915	-1.4439
18	N	1.2427	0.9389	1.2111
19	N	1.7941	0.7381	2.3842
20	Cu	2.2903	1.1785	-0.432
21	O	-2.2643	0.3129	0.2609
22	C	-3.1961	1.2076	0.884
23	H	-3.5172	1.9941	0.1916
24	H	-4.0553	0.59	1.1548
25	H	-2.7831	1.6729	1.7866
26	C	-0.1043	-1.7163	-4.2632
27	H	0.9202	-1.9353	-4.573
28	H	-0.6425	-2.6569	-4.1318
29	C	-0.6512	5.114	-2.6456
30	H	-1.6083	5.2437	-3.1577
31	H	-0.4136	6.0435	-2.1209
32	C	1.1085	-0.0623	4.5686
33	H	1.9229	0.5801	4.9094
34	H	0.2172	0.1512	5.1609
35	C	-0.8145	-0.7928	-5.2323
36	C	-0.3091	0.4934	-5.4764
37	C	-1.9912	-1.2037	-5.8659
38	C	-0.9787	1.3557	-6.3447
39	H	0.6037	0.8217	-4.9838
40	C	-2.6561	-0.3419	-6.7438
41	H	-2.3892	-2.1969	-5.6727
42	C	-2.1534	0.9387	-6.9811
43	H	-0.582	2.3506	-6.5228
44	H	-3.5685	-0.6705	-7.234
45	H	-2.6728	1.6114	-7.6583
46	C	1.4812	-1.5281	4.6165
47	C	0.5607	-2.4787	5.0714
48	C	2.7438	-1.9452	4.1722
49	C	0.8985	-3.8348	5.0863
50	H	-0.4185	-2.1566	5.4179
51	C	3.0792	-3.2996	4.1829
52	H	3.4604	-1.2071	3.82
53	C	2.1565	-4.2466	4.6402
54	H	0.1801	-4.5662	5.4463
55	H	4.0611	-3.6158	3.8411
56	H	2.4198	-5.3008	4.6519
57	C	0.4402	4.7184	-3.6206
58	C	0.2124	4.7971	-4.9984
59	C	1.6847	4.2688	-3.1543
60	C	1.2165	4.4356	-5.9026
61	H	-0.7522	5.139	-5.3658
62	C	2.6827	3.8971	-4.0555
63	H	1.8743	4.2096	-2.0848
64	C	2.4499	3.9791	-5.4328
65	H	1.0289	4.5028	-6.9708
66	H	3.638	3.5488	-3.6778
67	H	3.2277	3.6898	-6.1342
68	N	3.633	2.2582	0.3484
69	S	4.5635	2.2427	-0.9424
70	O	3.5345	1.4541	-1.8412
71	O	5.0236	3.5113	-1.5176
72	C	5.9652	1.1699	-0.765
73	C	5.8526	0.0442	0.0631
74	C	7.1394	1.4423	-1.4725
75	C	6.9351	-0.8209	0.1665
76	H	4.9403	-0.1353	0.6244
77	C	8.2121	0.5617	-1.3519
78	H	7.2113	2.3249	-2.0987
79	C	8.1286	-0.5782	-0.538
80	H	6.8622	-1.6939	0.8096
81	H	9.1302	0.7653	-1.8958
82	C	9.2862	-1.535	-0.4208
83	H	9.0523	-2.4864	-0.9164
84	H	10.1921	-1.1295	-0.8812
85	H	9.5036	-1.7652	0.6288

i4-+-oss

	Atom	X	Y	Z
1	C	-1.08	-1.013	0.0332
2	C	-0.7027	-1.8047	-1.2034
3	C	0.037	-1.0998	1.0614
4	C	-1.2174	0.4334	-0.4119
5	C	-1.3856	0.9192	-1.6923
6	H	-1.504	0.4385	-2.6507
7	C	-1.4562	-2.6336	-1.9993
8	H	-2.4621	-3.0113	-1.9117
9	C	0.0457	-1.5075	2.3751
10	H	-0.7236	-1.8849	3.0298

11	N	1.3309	-1.3304	2.7783
12	N	-1.2962	2.2596	-1.5598
13	N	-0.6477	-2.9194	-3.0535
14	N	-1.0434	1.4959	0.4294
15	N	-1.0804	2.6001	-0.2687
16	N	0.5327	-2.3162	-2.9505
17	N	0.4975	-1.6351	-1.8277
18	N	1.3071	-0.7178	0.7571
19	N	2.0973	-0.8632	1.7959
20	Cu	2.0199	-0.5032	-1.1647
21	O	-2.265	-1.612	0.5314
22	C	-3.0749	-0.7934	1.3858
23	H	-3.5798	-0.0023	0.8197
24	H	-3.8209	-1.4669	1.8133
25	H	-2.4915	-0.3366	2.193
26	C	-1.0053	-3.5984	-4.3114
27	H	-0.0701	-3.9844	-4.7225
28	H	-1.654	-4.4377	-4.0556
29	C	-1.0485	3.222	-2.6447
30	H	-1.8737	3.1597	-3.3579
31	H	-1.0513	4.2107	-2.1816
32	C	1.9331	-1.6686	4.0813
33	H	2.7644	-0.9737	4.2152
34	H	1.1769	-1.4627	4.8406
35	C	-1.6865	-2.6272	-5.255
36	C	-0.9805	-1.5211	-5.7506
37	C	-3.026	-2.8081	-5.6135
38	C	-1.6115	-0.6079	-6.5959
39	H	0.0596	-1.3763	-5.4704
40	C	-3.6558	-1.8962	-6.4663
41	H	-3.5762	-3.6628	-5.2275
42	C	-2.951	-0.7948	-6.956
43	H	-1.0572	0.2436	-6.9814
44	H	-4.6958	-2.0462	-6.743
45	H	-3.4403	-0.0851	-7.6175
46	C	2.3914	-3.1101	4.1263
47	C	1.6759	-4.0584	4.8653
48	C	3.5318	-3.5056	3.4132
49	C	2.0974	-5.391	4.8966
50	H	0.7928	-3.753	5.4211
51	C	3.951	-4.8361	3.4429
52	H	4.0865	-2.771	2.8355
53	C	3.2343	-5.7811	4.1853
54	H	1.5391	-6.1207	5.4765
55	H	4.8404	-5.1337	2.8945
56	H	3.5641	-6.8161	4.211
57	C	0.2815	2.881	-3.2877
58	C	0.3319	2.257	-4.5394
59	C	1.468	3.0758	-2.5663
60	C	1.556	1.8315	-5.067
61	H	-0.5877	2.0894	-5.0951
62	C	2.6899	2.653	-3.0936
63	H	1.4305	3.5461	-1.5867
64	C	2.7347	2.0239	-4.3441
65	H	1.5856	1.3463	-6.039
66	H	3.6059	2.8117	-2.5336
67	H	3.6854	1.679	-4.7377
68	N	3.6235	0.6321	-0.8224
69	S	4.5042	-0.1524	-1.9693
70	O	3.4349	-0.9293	-2.7382
71	O	5.421	0.6669	-2.7708
72	C	5.3925	-1.2927	-0.9431
73	C	4.7619	-2.4728	-0.519
74	C	6.6706	-0.9416	-0.4908
75	C	5.4358	-3.3109	0.3602
76	H	3.7746	-2.7356	-0.8856
77	C	7.3235	-1.7951	0.3925
78	H	7.143	-0.0274	-0.8346
79	C	6.7172	-2.9822	0.8386
80	H	4.9612	-4.2311	0.6865
81	H	8.3186	-1.5383	0.7442
82	C	7.4073	-3.8754	1.8347
83	H	7.2283	-4.9331	1.6129
84	H	8.4873	-3.6996	1.8521
85	H	7.0238	-3.6853	2.8461

i3-+

	Atom	X	Y	Z
1	C	-1.3262	-0.0057	0.0075
2	C	-0.2462	-0.4031	-0.9807
3	C	-0.7734	-0.0919	1.4212
4	C	-1.7271	1.4211	-0.3178
5	C	-1.5876	2.0931	-1.5166
6	H	-1.1895	1.804	-2.4759
7	C	-0.2627	-1.3019	-2.0218
8	H	-1.0101	-1.9903	-2.3814
9	C	-1.011	-1.013	2.4187
10	H	-1.6785	-1.8567	2.493
11	N	-0.1849	-0.6379	3.4276
12	N	-2.0365	3.3391	-1.2587
13	N	0.9573	-1.1645	-2.6043
14	N	-2.2591	2.2834	0.5955
15	N	-2.4442	3.4466	0.0255
16	N	1.6938	-0.2371	-1.9882

17	N	0.9622	0.2291	-1.0028
18	N	0.1837	0.7676	1.87
19	N	0.5417	0.4344	3.0905
20	Cu	1.6345	1.5102	0.4112
21	O	-2.3645	-0.964	-0.173
22	C	-3.6428	-0.6154	0.3677
23	H	-4.1177	0.1874	-0.2086
24	H	-4.2495	-1.5211	0.2936
25	H	-3.5763	-0.3075	1.4178
26	C	1.4651	-1.7773	-3.8336
27	H	2.5502	-1.8416	-3.7109
28	H	1.0679	-2.7931	-3.8815
29	C	-2.0695	4.4958	-2.1634
30	H	-2.946	4.4066	-2.8119
31	H	-2.2077	5.3679	-1.5186
32	C	0.0567	-1.2997	4.7231
33	H	-0.0419	-0.5364	5.4982
34	H	-0.7403	-2.0348	4.8494
35	C	1.0956	-0.9827	-5.0741
36	C	1.1348	0.4192	-5.0839
37	C	0.7505	-1.6661	-6.2459
38	C	0.8333	1.1229	-6.2515
39	H	1.396	0.9661	-4.1824
40	C	0.4567	-0.9609	-7.416
41	H	0.7125	-2.7529	-6.2435
42	C	0.4965	0.4353	-7.4211
43	H	0.8616	2.2085	-6.2421
44	H	0.1908	-1.5037	-8.3191
45	H	0.2636	0.985	-8.3291
46	C	1.4266	-1.9425	4.7442
47	C	1.6041	-3.2491	4.2715
48	C	2.5418	-1.2058	5.1649
49	C	2.8817	-3.8114	4.2155
50	H	0.7412	-3.8255	3.9458
51	C	3.8194	-1.7677	5.1093
52	H	2.408	-0.19	5.5265
53	C	3.9914	-3.0702	4.6314
54	H	3.0101	-4.8256	3.8477
55	H	4.6781	-1.1905	5.4415
56	H	4.9852	-3.5072	4.5868
57	C	-0.7992	4.5938	-2.9818
58	C	-0.8647	4.6292	-4.3785
59	C	0.4538	4.6407	-2.351
60	C	0.3051	4.7179	-5.1405
61	H	-1.8323	4.5876	-4.8726
62	C	1.6208	4.7227	-3.1109
63	H	0.5254	4.6068	-1.2667
64	C	1.5496	4.7591	-4.5081
65	H	0.2408	4.7503	-6.2249
66	H	2.5785	4.7613	-2.6036
67	H	2.4603	4.8227	-5.0978
68	N	3.1682	2.4832	1.2689
69	I	4.3906	1.1634	2.2799
70	S	4.0035	3.6135	0.3445
71	C	4.0953	-0.5507	1.0696
72	O	2.9054	4.4673	-0.1566
73	O	5.105	4.228	1.1087
74	C	4.7349	2.7653	-1.0533
75	C	4.8372	-0.7028	-0.1044
76	C	3.1058	-1.457	1.4617
77	C	3.9012	2.2119	-2.0307
78	C	6.1213	2.6259	-1.129
79	C	4.564	-1.8085	-0.9127
80	H	5.5941	0.0182	-0.3906
81	C	2.8526	-2.5546	0.6369
82	H	2.5475	-1.3132	2.3778
83	C	4.4687	1.5098	-3.0886
84	H	2.8258	2.326	-1.9618
85	C	6.6742	1.9183	-2.1985
86	H	6.7539	3.0659	-0.3658
87	C	3.5766	-2.7279	-0.5459
88	H	5.1256	-1.9456	-1.8321
89	H	2.0888	-3.2696	0.9272
90	C	5.861	1.3506	-3.1904
91	H	3.8205	1.0754	-3.8448
92	H	7.7536	1.8086	-2.2623
93	H	3.372	-3.5834	-1.1833
94	C	6.4557	0.5946	-4.3521
95	H	6.3945	1.1871	-5.2744
96	H	7.5092	0.3535	-4.1799
97	H	5.9129	-0.3402	-4.536

i1-+-s

	Atom	X	Y	Z
1	C	1.22	-1.7307	-0.1775
2	C	2.2015	-2.4286	-1.0993
3	C	1.5261	-2.0857	1.2665
4	C	1.3696	-0.2414	-0.4276
5	C	1.52	0.3821	-1.6516
6	H	1.587	0.0028	-2.6585
7	C	1.9791	-3.1987	-2.2163
8	H	1.0796	-3.5702	-2.6787
9	C	0.7468	-2.7054	2.2169
10	H	-0.2663	-3.0738	2.2072

11	N	1.5492	-2.7982	3.3067
12	N	1.6246	1.6923	-1.35
13	N	3.2198	-3.4424	-2.7108
14	N	1.3765	0.7135	0.544
15	N	1.5354	1.8874	-0.0161
16	N	4.1676	-2.8671	-1.9671
17	N	3.5477	-2.2502	-0.9849
18	N	2.7464	-1.8588	1.8265
19	N	2.7625	-2.2936	3.067
20	Cu	4.4139	-1.3788	0.6893
21	O	-0.0624	-2.2377	-0.5372
22	C	-1.1848	-1.4258	-0.1749
23	H	-1.2565	-0.5336	-0.8082
24	H	-2.0647	-2.0532	-0.3352
25	H	-1.1496	-1.1133	0.8753
26	C	3.5761	-4.102	-3.9774
27	H	4.5001	-4.6549	-3.8006
28	H	2.7753	-4.8156	-4.1854
29	C	1.8172	2.8301	-2.2595
30	H	0.8336	3.1883	-2.5771
31	H	2.2855	3.6118	-1.6549
32	C	1.2702	-3.469	4.5858
33	H	1.9597	-3.0277	5.3091
34	H	0.2495	-3.2106	4.8738
35	C	3.7377	-3.0968	-5.0994
36	C	4.9882	-2.8986	-5.6935
37	C	2.6372	-2.351	-5.5468
38	C	5.1376	-1.9753	-6.7329
39	H	5.8447	-3.4708	-5.3457
40	C	2.7881	-1.4212	-6.575
41	H	1.6612	-2.4997	-5.0905
42	C	4.0389	-1.2356	-7.1739
43	H	6.1119	-1.8333	-7.1927
44	H	1.9326	-0.8417	-6.9106
45	H	4.1537	-0.5145	-7.9779
46	C	1.4551	-4.9686	4.4743
47	C	0.3672	-5.8331	4.6338
48	C	2.7233	-5.4993	4.1984
49	C	0.5431	-7.2158	4.5231
50	H	-0.6178	-5.4249	4.8473
51	C	2.898	-6.8783	4.0825
52	H	3.5702	-4.8289	4.0741
53	C	1.8074	-7.7398	4.2458
54	H	-0.3071	-7.88	4.6519
55	H	3.8844	-7.2819	3.8703
56	H	1.945	-8.8141	4.1585
57	C	2.6713	2.4721	-3.4572
58	C	2.2739	2.8714	-4.7381
59	C	3.8755	1.7693	-3.301
60	C	3.0725	2.5838	-5.8488
61	H	1.3379	3.4096	-4.8673
62	C	4.6651	1.47	-4.4106
63	H	4.1918	1.4524	-2.3124
64	C	4.2689	1.8823	-5.6868
65	H	2.7544	2.9007	-6.8384
66	H	5.591	0.9175	-4.2773
67	H	4.8847	1.6475	-6.5499
68	C	5.9717	-0.6386	1.8759
69	H	5.5093	0.038	2.5917
70	C	6.2773	-0.2414	0.5933
71	H	6.9458	-0.8666	0.0013
72	H	6.4008	-1.5539	2.2807
73	C	5.9711	1.0893	0.0256
74	C	4.9051	1.8796	0.4961
75	C	6.7956	1.6056	-0.9898
76	C	4.7002	3.1642	-0.004
77	H	4.2261	1.4792	1.2435
78	C	6.5943	2.8952	-1.4829
79	H	7.6109	0.9982	-1.375
80	C	5.551	3.6816	-0.9867
81	H	3.8664	3.753	0.3659
82	H	7.247	3.2824	-2.2603
83	H	5.3893	4.6825	-1.377

add-+-t

	Atom	X	Y	Z
1	C	-2.2533	0.3431	-0.0386
2	C	-1.5343	-0.4735	-1.0909
3	C	-1.6937	-0.0236	1.3259
4	C	-1.9903	1.8095	-0.3304
5	C	-1.7128	2.4172	-1.5409
6	H	-1.5816	2.0356	-2.5418
7	C	-2.0088	-1.1378	-2.1964
8	H	-3.0031	-1.2989	-2.5798
9	C	-2.2935	-0.555	2.4441
10	H	-3.3118	-0.8302	2.6677
11	N	-1.2825	-0.6986	3.3381
12	N	-1.5782	3.7252	-1.2343
13	N	-0.8908	-1.6046	-2.8124
14	N	-2.005	2.7705	0.6378
15	N	-1.7527	3.9313	0.089
16	N	0.2152	-1.256	-2.1582
17	N	-0.1749	-0.5666	-1.116
18	N	-0.368	0.1029	1.6069

19	N	-0.1158	-0.3101	2.8284
20	Cu	1.0347	0.5781	0.1916
21	O	-3.6211	-0.0327	-0.1276
22	C	-4.5738	0.8837	0.4257
23	H	-4.6739	1.7816	-0.195
24	H	-5.5229	0.3431	0.437
25	H	-4.315	1.1843	1.4475
26	C	-0.769	-2.1841	-4.1609
27	H	0.1268	-2.8088	-4.1445
28	H	-1.6416	-2.8187	-4.3239
29	C	-1.2514	4.8436	-2.131
30	H	-2.0541	4.9308	-2.8677
31	H	-1.264	5.7332	-1.4968
32	C	-1.3299	-1.3148	4.6752
33	H	-0.4599	-0.9262	5.2094
34	H	-2.234	-0.951	5.1666
35	C	-0.6748	-1.0753	-5.1919
36	C	0.4162	-0.1929	-5.1697
37	C	-1.6893	-0.8981	-6.1386
38	C	0.4813	0.8589	-6.084
39	H	1.2061	-0.3232	-4.4342
40	C	-1.6173	0.1521	-7.0594
41	H	-2.5364	-1.5801	-6.1552
42	C	-0.5347	1.0341	-7.0304
43	H	1.3243	1.5432	-6.0543
44	H	-2.4081	0.2815	-7.7934
45	H	-0.4807	1.8538	-7.742
46	C	-1.3109	-2.826	4.5848
47	C	-2.4131	-3.5761	5.0075
48	C	-0.1871	-3.482	4.0623
49	C	-2.3931	-4.971	4.9128
50	H	-3.2856	-3.0701	5.4139
51	C	-0.1701	-4.8729	3.9614
52	H	0.6708	-2.9004	3.7334
53	C	-1.274	-5.6201	4.3874
54	H	-3.2522	-5.547	5.2459
55	H	0.7042	-5.3741	3.5551
56	H	-1.2589	-6.704	4.311
57	C	0.0901	4.6501	-2.8026
58	C	0.1704	4.4375	-4.1827
59	C	1.2654	4.669	-2.0379
60	C	1.4141	4.2588	-4.7964
61	H	-0.7389	4.4149	-4.7785
62	C	2.505	4.4762	-2.6466
63	H	1.2009	4.8328	-0.965
64	C	2.5809	4.2773	-4.0298
65	H	1.4682	4.1035	-5.8703
66	H	3.4109	4.476	-2.0473
67	H	3.5476	4.1353	-4.5037
68	N	2.2922	1.1905	-1.1177
69	S	3.4917	0.1612	-1.5913
70	O	3.8649	-0.7712	-0.5026
71	O	3.0768	-0.4057	-2.894
72	C	4.8764	1.2449	-1.8863
73	C	5.8468	1.421	-0.8979
74	C	4.9561	1.9172	-3.1102
75	C	6.9107	2.2851	-1.1471
76	H	5.7708	0.8916	0.0452
77	C	6.0261	2.778	-3.3392
78	H	4.1961	1.762	-3.8679
79	C	7.0154	2.9795	-2.3621
80	H	7.6697	2.4251	-0.3825
81	H	6.0987	3.2997	-4.29
82	C	8.1508	3.9437	-2.596
83	H	9.0908	3.5614	-2.1828
84	H	8.2976	4.1436	-3.6622
85	H	7.9478	4.9045	-2.1038
86	C	1.4168	2.6812	1.4144
87	H	1.8192	3.3424	0.6534
88	C	2.2247	2.0034	2.2726
89	H	1.7582	1.3348	2.9943
90	H	0.339	2.6871	1.5513
91	C	3.6855	2.0787	2.3289
92	C	4.3966	3.1856	1.8248
93	C	4.4017	1.0364	2.9481
94	C	5.7818	3.2484	1.9478
95	H	3.8591	4.0102	1.3666
96	C	5.7911	1.0898	3.0468
97	H	3.8597	0.1824	3.347
98	C	6.4836	2.2007	2.5545
99	H	6.3169	4.1139	1.5685
100	H	6.3317	0.2743	3.5188
101	H	7.5649	2.2526	2.6476

Substrate

	Atom	X	Y	Z
1	C	-2.1009	-0.0405	0.1413
2	C	-0.7229	-0.1316	0.3489
3	C	0.0853	0.9943	0.1827
4	C	-0.4615	2.2346	-0.1955
5	C	-1.854	2.3103	-0.3976
6	C	-2.6613	1.1871	-0.2318
7	H	-2.7341	-0.9142	0.2705
8	H	-0.2756	-1.0783	0.6408

9	H	1.1578	0.9165	0.347
10	H	-2.3098	3.2542	-0.6825
11	H	-3.7333	1.268	-0.393
12	C	0.4414	3.3879	-0.3597
13	H	1.4849	3.1826	-0.1188
14	C	0.1177	4.6233	-0.767
15	H	0.8762	5.3976	-0.8481
16	H	-0.8962	4.9119	-1.0341

Product

	Atom	X	Y	Z
1	C	0.9181	-2.7731	0.3089
2	C	2.2844	-2.9954	0.1217
3	C	3.1064	-1.9608	-0.3315
4	C	2.5723	-0.6928	-0.5955
5	C	1.1995	-0.4759	-0.4079
6	C	0.3784	-1.5106	0.0407
7	H	0.2765	-3.5781	0.6574
8	H	2.7117	-3.9743	0.3224
9	H	4.1689	-2.1367	-0.4828
10	H	0.7763	0.4998	-0.6294
11	H	-0.6849	-1.3324	0.1781
12	C	3.48	0.3945	-1.0615
13	C	3.4478	1.794	-0.5414
14	H	4.3923	2.3336	-0.5134
15	H	2.7101	2.0801	0.2036
16	H	4.4627	0.0641	-1.394
17	S	3.7226	2.0174	-3.2403
18	O	5.1799	1.933	-3.0238
19	O	3.1304	3.3109	-3.6095
20	C	3.2293	0.7615	-4.4025
21	C	4.1464	-0.2133	-4.7939
22	C	3.7322	-1.2084	-5.6806
23	C	2.4199	-1.241	-6.1715
24	C	1.5209	-0.2408	-5.7598
25	C	1.9142	0.762	-4.8803
26	H	5.163	-0.1909	-4.4164
27	H	4.4408	-1.9704	-5.9935
28	H	0.5009	-0.2507	-6.1355
29	H	1.2156	1.5319	-4.5693
30	C	1.9648	-2.325	-7.1163
31	H	1.537	-1.8964	-8.0307
32	H	2.7898	-2.9851	-7.4015
33	H	1.1825	-2.9403	-6.6538
34	N	2.8637	1.4868	-1.8589

PhI

	Atom	X	Y	Z
1	I	0.1618	-3.5938	-1.8415
2	C	0.341	-5.5337	-2.7015
3	C	1.5748	-5.9457	-3.212
4	C	-0.7772	-6.3706	-2.7414
5	H	2.4368	-5.2882	-3.1768
6	H	-1.7306	-6.0414	-2.3423
7	C	1.6836	-7.2222	-3.7725
8	C	-0.6507	-7.6437	-3.3058
9	H	2.6408	-7.5481	-4.1703
10	H	-1.5173	-8.2986	-3.339
11	C	0.5756	-8.0719	-3.82
12	H	0.667	-9.0627	-4.2561

PhINTs-SP

	Atom	X	Y	Z
1	C	-0.0458	1.3365	-0.035
2	C	0.5262	2.1265	-1.0365
3	C	0.3426	1.7434	-2.3648
4	C	-0.3776	0.6029	-2.7254
5	C	-0.9328	-0.1773	-1.7092
6	C	-0.7708	0.1902	-0.3696
7	H	0.0924	1.614	1.006
8	H	1.1101	3.0039	-0.7818
9	H	-0.4909	0.3208	-3.767
10	H	-1.4932	-1.0712	-1.9679
11	H	-1.2062	-0.4216	0.4153
12	I	1.2406	2.9023	-3.9078
13	N	2.4672	1.5105	-4.7476
14	S	3.9673	1.4313	-4.041
15	O	4.6342	0.379	-4.8382
16	O	4.6558	2.7345	-3.8976
17	C	3.7205	0.8012	-2.3761
18	C	3.0432	-0.4109	-2.2027
19	C	4.1464	1.543	-1.2758
20	C	2.7924	-0.872	-0.9149
21	H	2.6995	-0.9731	-3.0652
22	C	3.8878	1.066	0.0117
23	H	4.6636	2.4842	-1.4281
24	C	3.2052	-0.1402	0.2123
25	H	2.2535	-1.806	-0.7771
26	H	4.2126	1.6474	0.8711
27	C	2.9103	-0.6527	1.6001
28	H	3.5591	-1.5022	1.8515
29	H	3.0679	0.1219	2.3576
30	H	1.8749	-1.0046	1.6794

6- References.

(1) Ozkal, E.; Llanes, P.; Bravo, F.; Ferrali, A.; Pericàs, M. A. Fine-Tunable Tris(triazolyl)methane Ligands for Copper(I)- Catalyzed Azide–Alkyne Cycloaddition Reactions. *Adv. Synth. Catal.* **2014**, *356*, 857−869.

(2) Rodríguez, M. R.; Molina, F.; Etayo, P.; Pericàs, M. A.; Pérez, P. J.; Díaz-Requejo, M. M. Heterogeneous Olefin Aziridination Reactions Catalyzed by Polymer-Bound Tris(triazolyl)methane Copper Complexes. *Eur. J. Inorg. Chem.* **2021**, *36*, 3727–3730.

(3) Yamada, Y.; Yamamoto, T.; Okawara, M. Synthesis and Reaction of New Type I–N Ylide, N-tosyliminoiodinane. *Chem. Lett.* **1975**, 361–362.

(4) Matsuzawa, K.; Nagasawa, Y.; Yamaguchi, E.; Tada, N.; Itoh, A. An Efficient Aziridination of Styrenes Promoted by Visible Light. *Synthesis* **2016***, 48*, 2845−2850.

(5) Maestre, L.; Sameera, W. M. C.; Díaz-Requejo, M. M.; Maseras, F.; Pérez, P. J. A General Mechanism for the Copper- and Silver-Catalyzed Olefin Aziridination Reactions: Concomitant Involvement of the Singlet and Triplet Pathways. *J. Am. Chem. Soc.* **2013**, *135*, 1338−1348.

(6) Saikia, I.; Kashyap, B.; Phukan, P. A. Facile Noncatalytic Pathway for Nitrene Transfer Process: Expeditious Access to Aziridines *Chem. Commun.* **2011**, *47*, 2967– 2969.

(7) Au, S.-M.; Huang, J.-S.; Yu, W.-Y.; Fung, W.- H.; Che, C.-M. Aziridination of Alkenes and Amidation of Alkanes by Bis(tosylimido)ruthenium(VI) Porphyrins. A Mechanistic Study. *J. Am. Chem. Soc.* **1999**, *121*, 9120-9132.

(8) Jiang, X.; Ji, G. A. Self-Consistent and Cross-Checked Scale of Spin-Delocalization Substituent Constants, the $\sigma^{\cdot}_{JJ}$ Scale *J. Org. Chem.* **1992**, *57*, 6051−6056.

(9) Dinctürk, S.; Jackson, R. A. Free Radical Reactions in Solution. Part 7. Substituent Effects on Free Radical Reactions: Comparison of the $\sigma^{\cdot}$ Scale with Other Measures of Radical Stabilization *J. Chem. Soc., Perkin Trans. 2* **1981**, 1127−1131.

(10) Fisher, T. H.; Meierhoefer, A. W. Substituent Effects in Free-Radical Reactions. A Study of 4-Substituted 3-Cyanobenzyl Free Radicals *J. Org. Chem.* **1978**, *43*, 224−228.
